# Supplementary material for: An auto-initializing algorithm to determine electronic ground states from random Slater determinants
Source: Nat Commun. 2026 Sep 29;17:11225. doi: 10.1038/s41467-026-77968-1 (PMC13720135; doi:10.1038/s41467-026-77968-1)
Supplement: Supplementary file 1 [file 41467_2026_77968_MOESM1_ESM.pdf]

Supplementary Information:
An auto-initializing algorithm to determine electronic ground states from random Slater determinants

CONTENTS

**Suppl. Note 1**	**Randomly Generated Strong-Correlation Benchmark**		**2**
	1.1	Motivation	3
	1.2	Experimental Design	3
	1.3	Results	6
	1.4	Discussion	7
**Suppl. Note 2**	**Robustness: Multi-Run Variance and Seed Convergence**		**9**
	2.1	Setup	9
	2.2	Results	9
**Suppl. Note 3**	**Ablation Study: Understanding the Sources of Compactness**		**10**
	3.1	Experimental Design	11
	3.2	TRIM is the dominant driver of compactness	11
	3.3	The TRIM advantage persists at large scale	12
	3.4	Hyperparameter insensitivity	13
**Suppl. Note 4**	**Physical Validation: Wavefunction Analysis**		**15**
	4.1	SHCI basin escape	15
	4.2	Leading determinant analysis	15
	4.3	Orbital occupation and 3d bond density	17
	4.4	Convergence comparison	18
**Suppl. Note 5**	**Physical Validation: Hubbard Model**		**19**
	5.1	Energy and AFM order parameter	19
**Suppl. Note 6**	**Cr_2 cc-pVDZ-DK: Large-Basis Benchmark**		**20**
	6.1	Hamiltonian construction	21

6.2 TrimCI workflow 21
6.3 Results and comparison with published SHCI 22

Suppl. Note 7 Recovering from Random Orbitals 23
7.1 TrimCI rotates random orbitals back to a basis supporting FCI extrapolation 24
7.2 Visualizing the recovered basis 27
7.3 Comparison with SHCI orbital optimization 28
7.4 Comparison with DMRG-CASSCF 32
7.5 Why this matters 34

Suppl. Note 8 Practical Scale and Scaling Path 34
8.1 TrimCI at 2.56×10^9 determinants on a single GPU node 34
8.2 Performance on workstation-class GPUs 35
8.3 Empirical scaling law 36
8.4 Scaling to trillion determinants 37

Supplementary References 38

Suppl. Note 1. RANDOMLY GENERATED STRONG-CORRELATION BENCHMARK

In practice, researchers routinely encounter strongly correlated systems for which reliable physical or chemical intuition is unavailable. Examples include scanning phase diagrams of strongly correlated lattice models, sweeping bond-distance curves of multi-reference molecules, and exploring families of transition-metal complexes whose ground-state character is not known a priori. Across such studies, hand-crafting an initialization for each problem quickly becomes impractical, and what is needed is a method whose accuracy does not depend on problem-specific explicit knowledge.

This raises a natural question: do performance differences between quantum many-body algorithms reflect genuine algorithmic advantages, or simply the quality of human-supplied initialization? To disentangle these factors, we design a *randomly generated strong-correlation benchmark* where no method has access to explicit knowledge about the system. The rationale is that a method performing well on unknown, randomly transformed prob-

lems is more likely to generalize reliably to genuinely unfamiliar systems encountered in future applications.

1.1. Motivation

For well-studied molecules, decades of physical and chemical intuition provide initialization strategies—localized orbitals, broken-symmetry determinants, physical spin configurations—that are *themselves* explicit knowledge. When a selected CI method is given the right starting point for the molecular system (e.g., a correct ground-state broken-symmetry determinant on localized orbitals for FeS clusters), it performs well precisely because the initialization encodes the answer. This makes it impossible to separate algorithmic merit from the quality of human-supplied heuristics.

We therefore construct a test bed of unknown systems that preserves the computational difficulty of [4Fe-4S] (CAS(36o, 54e), strong correlation, large Hilbert space) while removing all avenues for explicit-knowledge-based initialization:

- No method can exploit a known orbital ordering, because the rotation strategy scrambles the orbital basis and the noise strategy perturbs the Hamiltonian.
- No method can "happen to" start near the ground state, because both strategies break any system-specific structure that an initialization heuristic could exploit.
- The difficulty level remains realistic: these are not toy models but 36-orbital, 54-electron systems with strong multireference character.

This provides a level playing field where the only variable is the algorithm itself.

1.2. Experimental Design

Starting from the [4Fe-4S] FCIDUMP (CAS(36o, 54e), localized orbitals from Ref. [1]), we generate 10 unknown systems via two complementary strategies:

- **Orbital rotation** (rot_01–05): A random unitary matrix $U \in O(36)$ is applied to the

one-electron integrals h_{pq} and two-electron integrals g_{pqrs}:

$$h'_{pq} = \sum_{rs} U_{pr}\, U_{qs}\, h_{rs}, \tag{1}$$

$$g'_{pqrs} = \sum_{abcd} U_{pa}\, U_{qb}\, U_{rc}\, U_{sd}\, g_{abcd}. \tag{2}$$

This preserves the exact eigenvalues but completely destroys the localized orbital structure that physical initialization strategies rely on.

- **Noise perturbation** (noise_01–05): Gaussian noise is added to all integrals:

$$h'_{pq} = h_{pq} + \delta h_{pq}, \quad \delta h_{pq} \sim \mathcal{N}(0,\, 0.05\,\sigma_h), \tag{3}$$

$$g'_{pqrs} = g_{pqrs} + \delta g_{pqrs}, \quad \delta g_{pqrs} \sim \mathcal{N}(0,\, 0.05\,\sigma_g), \tag{4}$$

where σ_h and σ_g are the standard deviations of the original integrals. The noise level (5% of the standard deviation) creates genuinely different Hamiltonians while preserving the sparsity structure. Hermitian symmetry of the one-electron integrals ($h'_{pq} = h'_{qp}$) and full 8-fold symmetry of the two-electron integrals are enforced after perturbation.

For each of the 10 systems, three calculations are performed:

1. **SHCI (HF init)**: Standard SHCI starting from the Hartree–Fock determinant.

2. **SHCI (open-shell init)**: SHCI starting from an open-shell determinant where α electrons occupy the leftmost orbitals and β electrons occupy the rightmost, creating a maximally broken-symmetry initial state.

3. **TrimCI**: Standard TrimCI with random initialization.

All three calculations use the same FCIDUMP and the same computational resources (48 CPU cores, up to 1 TB RAM). Crucially, all TrimCI hyperparameters are fixed across the 10 systems—no system-specific tuning is performed. The complete hyperparameter set is listed in Suppl. Table 1.

Within each problem, all three methods (SHCI with HF init, SHCI with open-shell init, TrimCI) consume the same scrambled FCIDUMP, so the orbital basis is identical across methods within a problem. Any energy difference within a problem therefore reflects the algorithm, not the input.

SHCI calculations are performed using the Arrow code [2–4], the original implementation of the semistochastic heat-bath CI algorithm. Variational iterations use $\varepsilon_1 \in \{10^{-3}, 5 \times 10^{-4}, 2 \times 10^{-4}\}$, followed by semistochastic Epstein–Nesbet PT2. TrimCI expands from 100 determinants (Phase A) to 10^7 determinants (Phase B) with PT2 at each expansion step.

Suppl. Table 1. TrimCI hyperparameters used for all 10 benchmark systems. The same parameter set is used without modification across all systems.

Parameter	Value	Description
Phase A: Search (→ 100 determinants)		
`num_runs`	64	Independent TrimCI runs
`initial_dets`	1 HF + 10 000 random	Starting determinants per run
`first_cycle_keep_size`	10	Determinants kept after first trim
`max_rounds`	4	Trim–expand iterations
`max_final_dets`	100	Target size after Phase A
`pool_core_ratio`	40	Pool-to-core size ratio
`num_groups`	20	Number of local trimming groups
`local_trim_keep_ratio`	0.1	Fraction kept per group relative to core size
`core_set_ratio`	[1, 1.01]	Core set growth schedule (repeated)
`threshold`	10^{-2}	Initial heat-bath screening threshold
`pool_build_strategy`	heat_bath	Candidate generation method
`orbital_optimization`	False	No orbital rotation
Phase B: Expansion (100 → 10^7 determinants)		
`growth_factor`	2.0	Determinant count doubles each round
`max_n_dets`	10^7	Final target size
`orbital_optimization`	False	No orbital rotation
`pt2_correction`	True	Compute PT2 at each step

1.3. Results

Suppl. Fig. 1 shows the energy convergence for all 10 systems. In every case, TrimCI (orange) achieves lower variational energy and lower var+PT2 energy than both SHCI initializations at comparable or smaller determinant counts.

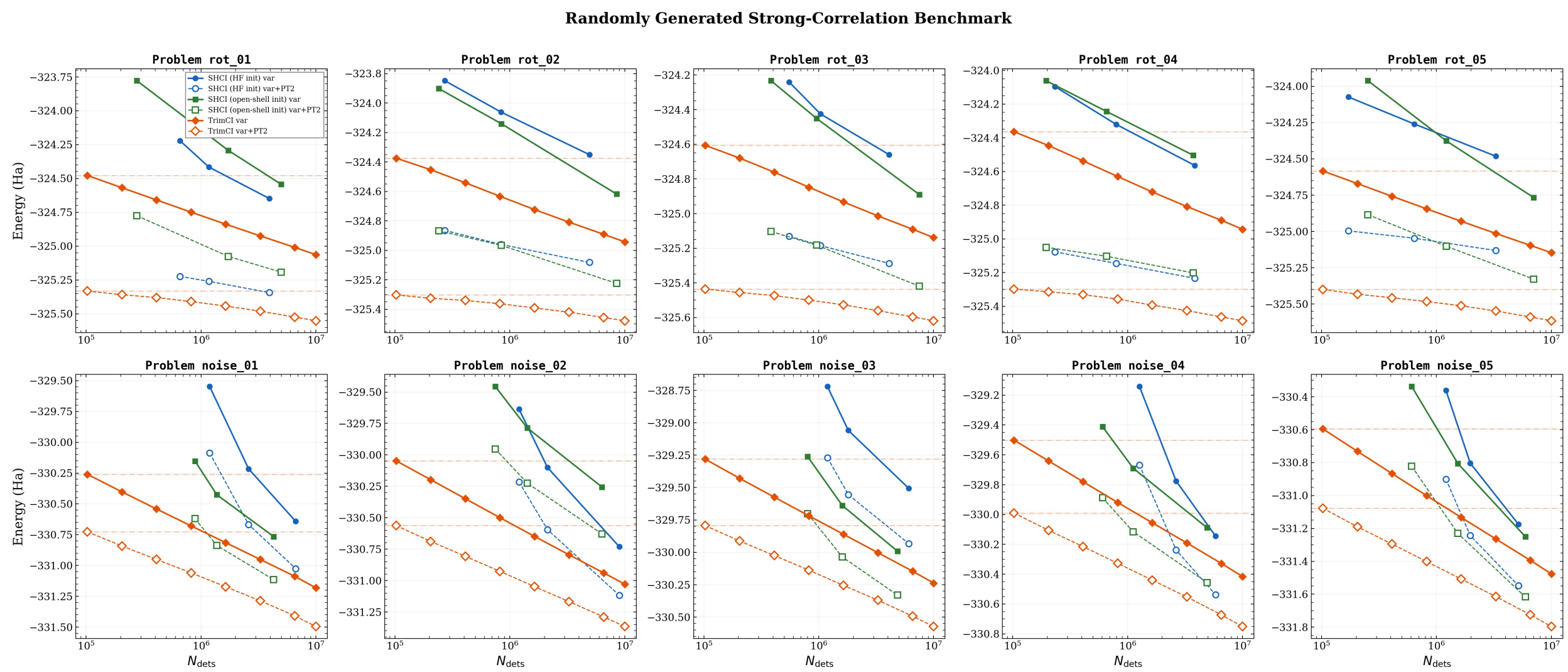

Suppl. Fig. 1. Energy vs. number of determinants for 10 benchmark systems derived from [4Fe-4S]. Top row: scrambled orbitals. Bottom row: noise perturbation (5% of integral std, different Hamiltonians). Solid lines: variational energy E_{var}. Dashed lines with open markers: $E_{\text{var}}+\Delta E_{\text{PT2}}$. Blue: SHCI with HF initialization. Green: SHCI with open-shell initialization. Orange: TrimCI. TrimCI achieves lower energy in all 10 systems at both variational and PT2-corrected levels. Within each panel the orbital basis is held fixed across methods (all three methods see the same scrambled FCIDUMP). Source data are provided as a Source Data file.

Suppl. Fig. 2 quantifies the efficiency advantage as the ratio of SHCI to TrimCI determinant counts required to reach the same energy.

Suppl. Table 2 quantifies the efficiency advantage: for each system, we report the ratio of SHCI determinants to TrimCI determinants required to reach the same energy. TrimCI energies are taken at $N_{\text{dets}} = 10^5$; SHCI determinant counts are obtained by interpolation or extrapolation (*) from the available ε_1 schedule. Both SHCI initializations (HF and open-shell) are shown separately.

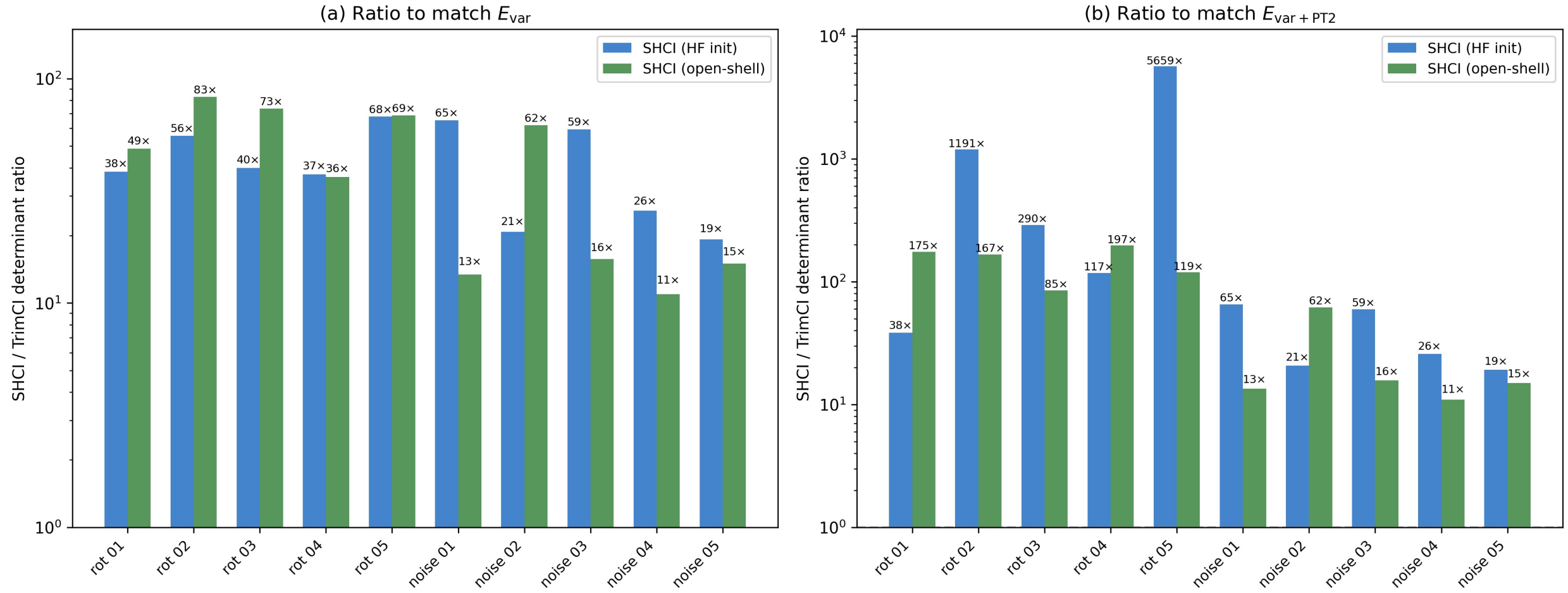

Suppl. Fig. 2. Efficiency ratio: number of SHCI determinants divided by TrimCI determinants (10^5) required to reach the same energy. (a) Variational energy match. (b) Var+PT2 energy match. Blue: SHCI with HF initialization. Green: SHCI with open-shell initialization. All ratios > 1, indicating TrimCI is uniformly more efficient. Note logarithmic scale; the largest ratios ($> 1000\times$ for rot_02 HF in panel b) reflect SHCI being trapped in a poor basin where PT2 fails accordingly. Source data are provided as a Source Data file.

1.4. Discussion

Four key conclusions follow from the benchmark:

1. TrimCI wins uniformly without problem-specific explicit knowledge. Across all 10 systems—spanning two qualitatively different perturbation types—TrimCI achieves lower energy than SHCI with either generic initialization strategy at both the variational and var+PT2 levels (Suppl. Fig. 2).

2. The efficiency advantage is large and consistent. To match TrimCI's variational energy at 10^5 determinants, SHCI requires on average 39× (HF init) or 33× (open-shell init) more determinants (geometric mean over 10 systems; Suppl. Table 2). At the var+PT2 level, the gap widens further: SHCI requires 113× (HF) or 51× (open-shell) more determinants on average. The larger PT2 ratios reflect the fact that when SHCI is trapped in a poor variational basin, the PT2 correction is also degraded—a compounding disadvantage that TrimCI avoids through its self-correcting trim mechanism.

3. SHCI performance is initialization-dependent; TrimCI is not. For a given system, the two SHCI initializations can differ by up to 0.15 Ha in variational energy, reflecting

Suppl. Table 2. Efficiency ratio: SHCI N_{dets} / TrimCI N_{dets} to match TrimCI at 10^5 determinants. Higher ratio = larger TrimCI advantage. *Extrapolated from the two largest SHCI calculations.

System	TrimCI @ 10^5 dets		Ratio to match E_{var}		Ratio to match $E_{\text{var+PT2}}$	
	E_{var} (Ha)	$E_{\text{var+PT2}}$ (Ha)	SHCI (HF)	SHCI (OS)	SHCI (HF)	SHCI (OS)
rot_01	−324.477	−325.332	38×	49×	38×	175×*
rot_02	−324.374	−325.302	56×*	83×	1191×*	167×*
rot_03	−324.606	−325.436	40×	73×	290×*	85×*
rot_04	−324.364	−325.298	37×	36×	117×*	197×*
rot_05	−324.584	−325.400	68×*	69×	5659×*	119×*
noise_01	−330.260	−330.727	65×	13×	65×	13×
noise_02	−330.047	−330.563	21×	62×	21×	62×
noise_03	−329.280	−329.793	59×	16×	59×	16×
noise_04	−329.501	−329.990	26×	11×	26×	11×
noise_05	−330.595	−331.077	19×	15×	19×	15×
		Geometric mean:	39×	33×	113×	51×

sensitivity to the (arbitrary) choice of starting determinant. In the worst cases (rot_02, rot_05), SHCI with HF initialization requires over 1,000× more determinants than TrimCI at the var+PT2 level. In contrast, TrimCI's random initialization + trim strategy is self-correcting: it explores the Hilbert space broadly in Phase A and selects the most important determinants through iterative trimming, regardless of orbital structure.

4. Implications for practical applications. What unites these scenarios is that expert initialization is either unavailable or impractical to supply for every system. The 30–100× efficiency advantage demonstrated here suggests that TrimCI can serve as a reliable, automated solver in such settings, without sacrificing accuracy.

We emphasize that the subject of study in this section and the sections below is *algorithmic behavior*, which we probe by controlled experiments on scrambled orbital bases. The absolute energies reported here should therefore not be confused with TrimCI's production accuracy: in the production setting, where orbital optimization is enabled, the residual energy is much smaller and lies in the milli-Hartree range relevant for chemical and physical

prediction (see SI S7 and SI S8).

Suppl. Note 2. ROBUSTNESS: MULTI-RUN VARIANCE AND SEED CONVERGENCE

A key concern for any stochastic method is whether reported results are robust or cherry-picked. TrimCI's Phase A runs multiple independent searches from different random initial determinants and selects the best. Here we characterize the variance across runs and determine how many runs are sufficient.

2.1. Setup

We use the same system as in S1 (Problem `rot_01`, randomized [4Fe-4S], CAS(36o, 54e)). Phase A is run 64 times independently, each starting from 1 reference + 10,000 random determinants and trimming to 100 determinants. All hyperparameters are identical across runs (Suppl. Table 1); the only source of variation is the random initial determinant set.

For reference, we also run SHCI on the same system with two generic initializations (default and open-shell) at $\varepsilon_1 = 0.05$, yielding 123 and 127 determinants, respectively—comparable in size to TrimCI's 100 determinants.

2.2. Results

Suppl. Fig. 3 summarizes the results.

Three observations:

1. TrimCI is uniformly superior across all runs. The 64 runs span an energy range of 0.42 Ha (best: −323.562 Ha, worst: −323.138 Ha, mean: -323.394 ± 0.089 Ha). *Every single run*—including the worst—produces a lower energy than SHCI with either initialization at comparable determinant count (−322.253 Ha for default init, −322.336 Ha for open-shell init). The advantage is robust: any randomly chosen run outperforms SHCI.

2. Modest number of runs suffices. Suppl. Fig. 3(b) shows that the best-of-k energy converges rapidly: ∼20 runs reach within 0.01 Ha of the 64-run optimum. This is far from the exponential scaling with system size that would be required if the method merely shifted

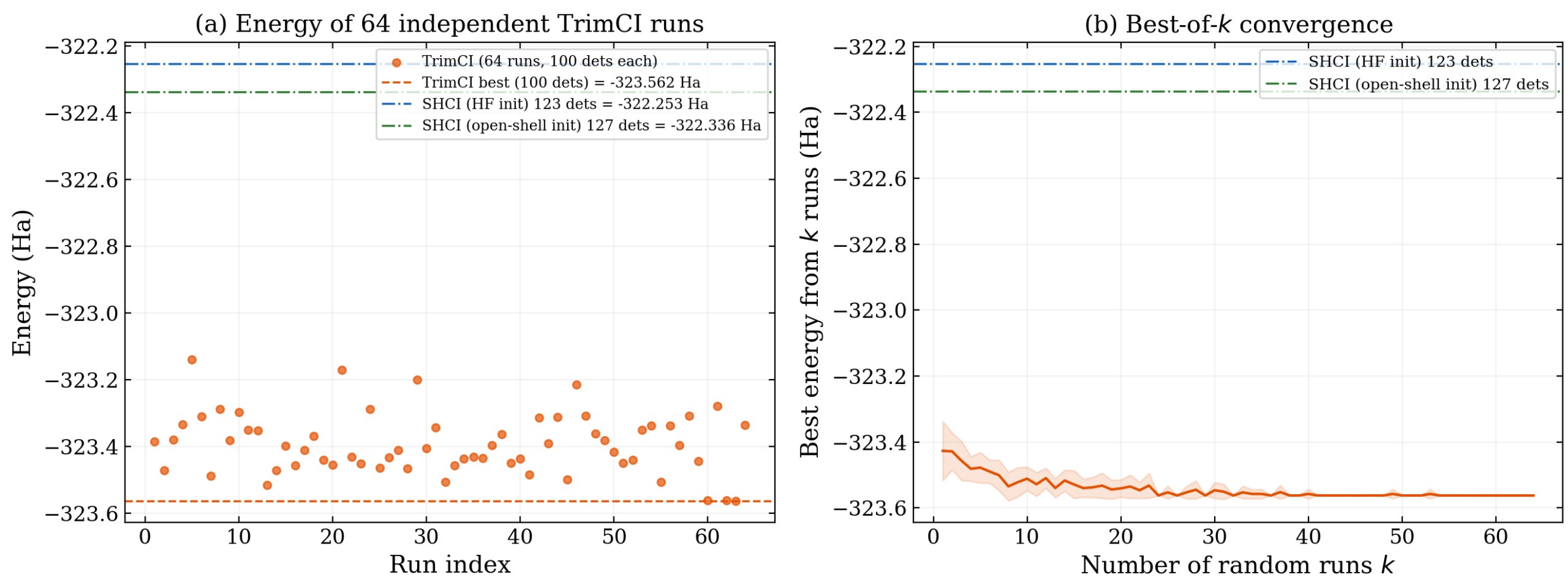

Suppl. Fig. 3. Robustness of TrimCI across 64 independent runs on Problem `rot_01` (100 determinants each). (a) Energy of each run (orange dots), with the best TrimCI energy (dashed orange), SHCI with default initialization (123 dets, blue dash-dot), and SHCI with open-shell initialization (127 dets, green dash-dot) shown as reference lines. Every TrimCI run—including the worst—achieves lower energy than either SHCI initialization at comparable determinant count. (b) Best-of-k energy as a function of the number of randomly selected runs (averaged over 10 random subsamples), showing rapid convergence: ∼20 runs suffice to reach near-optimal energy. Source data are provided as a Source Data file.

complexity to the initialization stage.

3. SHCI from default initialization is deterministic. In contrast, SHCI's heat-bath expansion from a single determinant is fully deterministic—repeated runs produce identical results (as confirmed in S3, where the no-trim baseline shows $\sigma = 0$). This means SHCI has no mechanism to escape a poor initialization. TrimCI's stochastic exploration, combined with iterative trimming, provides a self-correcting search that consistently finds lower-energy determinant spaces.

Suppl. Note 3. ABLATION STUDY: UNDERSTANDING THE SOURCES OF COMPACTNESS

A natural question is what fundamentally drives TrimCI's more compact expansions compared to SHCI. Two candidate mechanisms are: (i) the TRIM step (local-then-global determinant selection within the iterative workflow), and (ii) the random initialization (ensemble

of independent searches from random starting points). Here we isolate their contributions through controlled experiments on Problem `rot_01`.

3.1. Experimental Design

We compare TrimCI with TRIM enabled (the full method) against TrimCI with the TRIM procedure disabled—i.e., all trimming steps are skipped, reducing the expansion to a pure heat-bath selection equivalent to the SHCI expansion strategy. Both use the same FCIDUMP, the same target of 100 determinants, and 64 independent runs with different random seeds.

Five initialization conditions are tested: 1 HF determinant only, 1 HF + 1 random, 1 HF + 10 random, 1 random (no HF), and 10 random (no HF). For reference, we also show SHCI results at comparable determinant count ($\varepsilon_1 = 0.05$, yielding 123–127 determinants).

3.2. TRIM is the dominant driver of compactness

Suppl. Fig. 4 shows the effect of TRIM at 100 determinants.

With TRIM enabled, all five initializations reach best energies in the range -323.5 to -323.6 Ha—well below both SHCI references. This is the case regardless of initialization, including the purely random starts (1 R and 10 R) that contain no HF determinant. With TRIM disabled, the energies are significantly higher: -322.4 Ha for 1 HF (a gap of 1.14 Ha), -323.0 Ha for 1 HF + 1 random (0.51 Ha), -323.0 Ha for 1 HF + 10 random (0.60 Ha), -323.1 Ha for 1 R (0.47 Ha), and -323.2 Ha for 10 R (0.40 Ha).

Three conclusions emerge. First, **TRIM is the dominant mechanism**: it improves the energy by 0.4–1.1 Ha at 100 determinants, dwarfing the effect of initialization choice. Second, without TRIM, expansion from a single HF determinant is fully deterministic ($\sigma = 0$ across 64 runs), confirming that SHCI-style expansion has no stochastic exploration mechanism. Adding random determinants introduces variance but does not recover the TRIM advantage. Third, **TrimCI escapes the HF basin**: even when initialized from the single HF determinant—which produces a deterministic, trapped expansion under SHCI—TRIM-on reaches similar low energy as the random-seeded runs, demonstrating that the trim mechanism actively corrects a poor starting point.

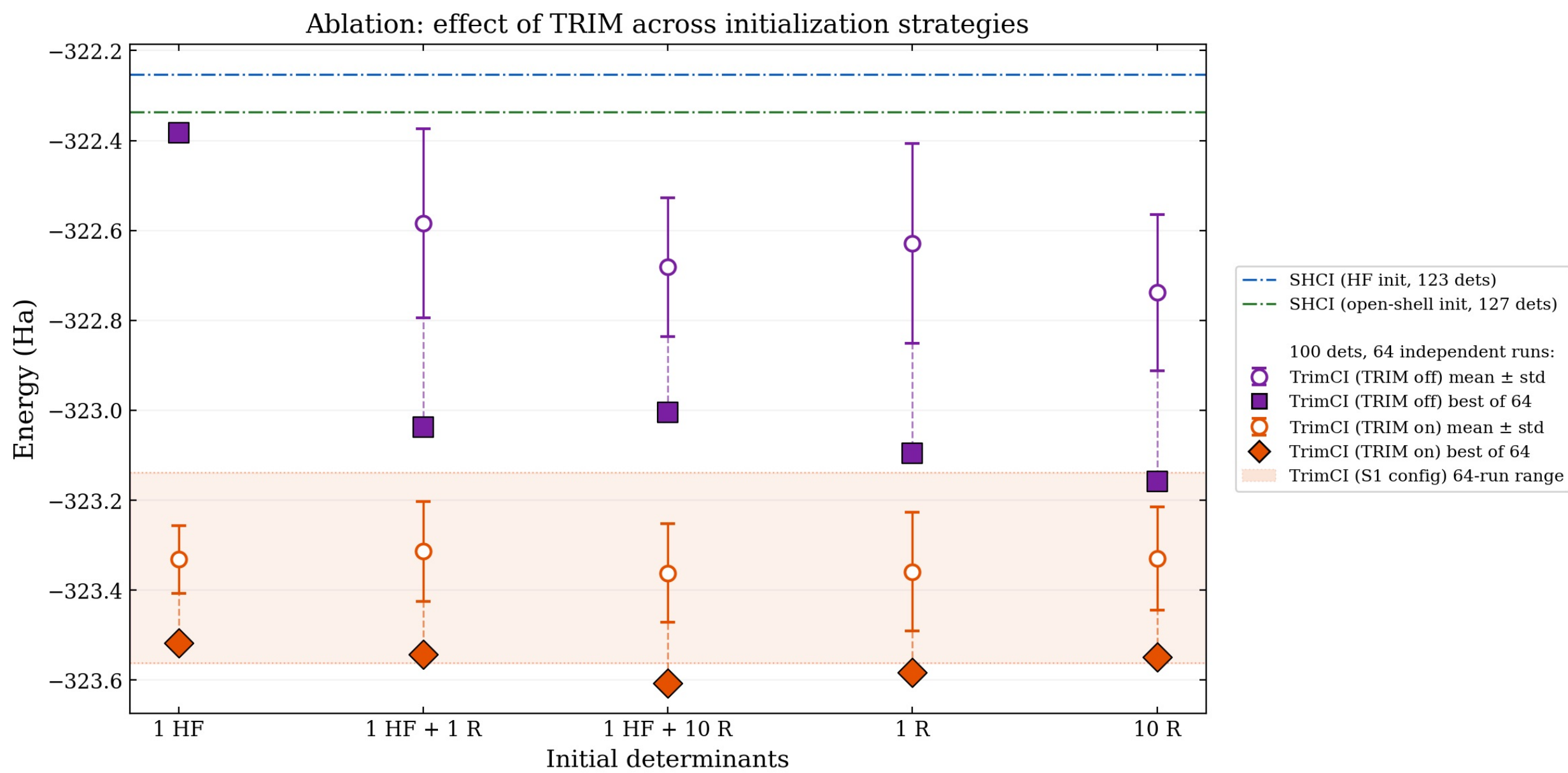

Suppl. Fig. 4. Ablation: effect of TRIM across initialization strategies. Filled markers: best of 64 runs (diamonds = TRIM on, squares = TRIM off). Open circles with error bars: mean ± std of 64 runs. Dashed lines connect the mean to the best. Shaded orange band: full range of 64 TrimCI runs with the S1 default configuration. Dashed horizontal lines: SHCI at comparable size. Five initializations are tested, including two purely random (no HF determinant). TRIM lowers the energy by 0.5–1.1 Ha regardless of initialization strategy. Source data are provided as a Source Data file.

3.3. The TRIM advantage persists at large scale

To verify that the advantage at 100 determinants is not an artifact of the small space, we expand both the TRIM-on and TRIM-off results (1 HF init, 100 dets) to 10^7 determinants using the same Phase B expansion protocol. For comparison, we also include TRIM-off with random initializations (1 HF+1R, 1 HF+10R), the full TrimCI from S1 (TRIM-on, 1 HF+10K R), and SHCI with both HF and open-shell initialization.

Suppl. Fig. 5 shows that TrimCI (TRIM on) maintains a consistent advantage over the TRIM-off expansion at every scale. Two TRIM-on curves are shown: the full method (1 HF + 10k R) and a HF-only initialization, both reaching similar energies at 10^7 determinants. Multiple TRIM-off curves (1 HF, 1 HF+1R, 1 HF+10R) are also shown, all with higher energy throughout. At 10^7 determinants, the gap between TRIM-on and TRIM-off is 0.44 Ha

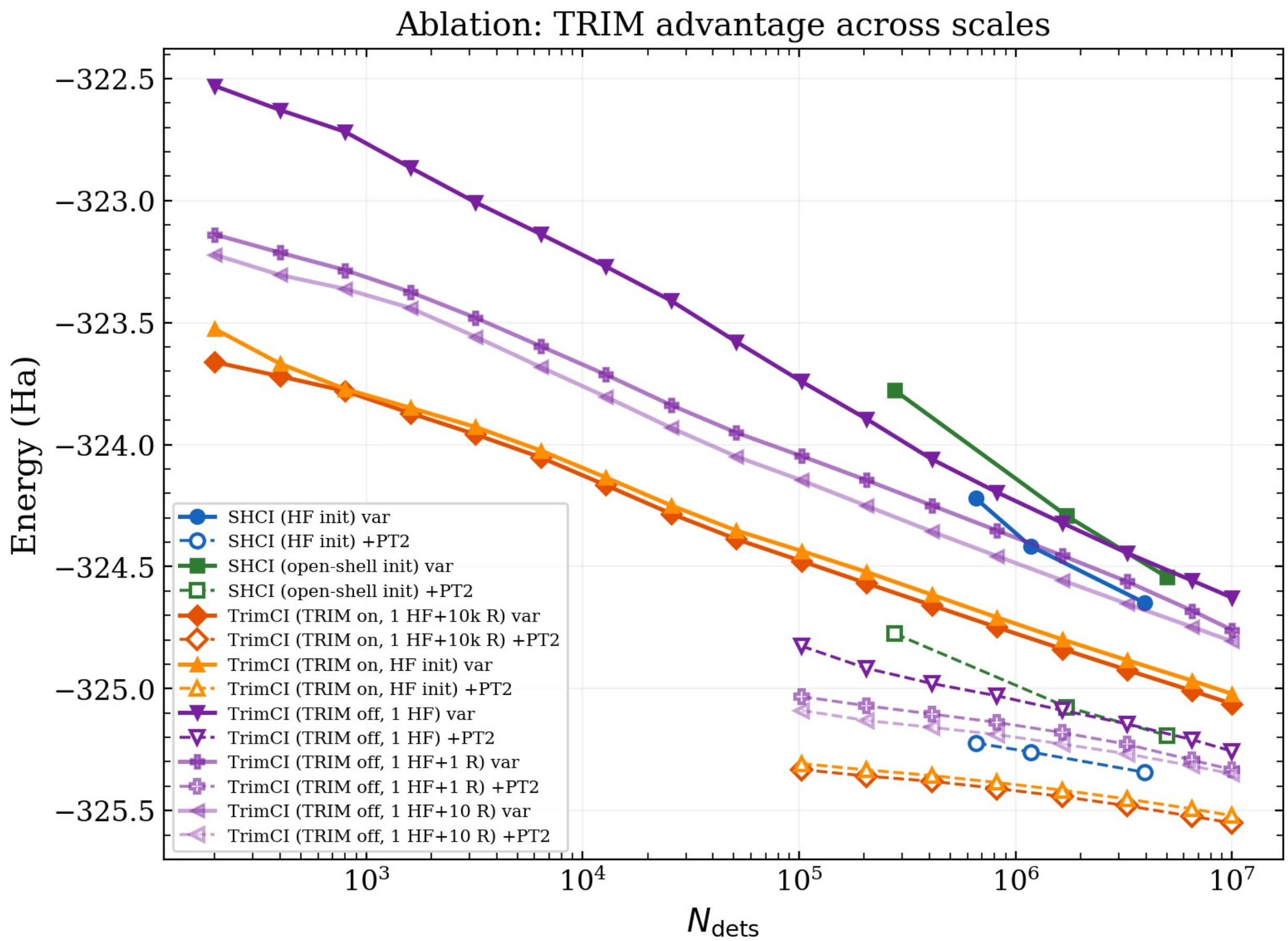

Suppl. Fig. 5. Ablation: TRIM advantage across scales. Orange diamonds: TrimCI (TRIM on, 1 HF+10k R). Amber triangles: TrimCI (TRIM on, HF init only). Purple: TrimCI (TRIM off) with various initializations. Blue/green: SHCI with two initializations. Solid lines: variational energy; dashed lines (shown for $N_{\text{dets}} \geq 10^5$): var+PT2. The TRIM advantage narrows from 1.1 Ha at 100 dets to 0.29 Ha at 10^7 dets (PT2-corrected), but never closes. Source data are provided as a Source Data file.

in variational energy and 0.29 Ha after PT2 correction. Including PT2, TrimCI (TRIM-on) remains lower in energy than all other curves—TRIM-off, SHCI (HF), and SHCI (open-shell)—and is insensitive to the choice of initialization.

3.4. Hyperparameter insensitivity

Finally, we verify that TrimCI's performance is not sensitive to the choice of hyperparameters. We vary two key parameters independently:

- **Initial determinants** (with local trim keep ratio lkr = 0.1 fixed): 1 HF, 1 HF+1R, 1 HF+10R, 1 HF+100R, 1 HF+1000R, 1 HF+10000R, 1 R (no HF), 10 R (no HF).

- **Local trim keep ratio** (with 1 HF+10000R init fixed): lkr = 0.05, 0.10, 0.20, 0.50, 1.00.

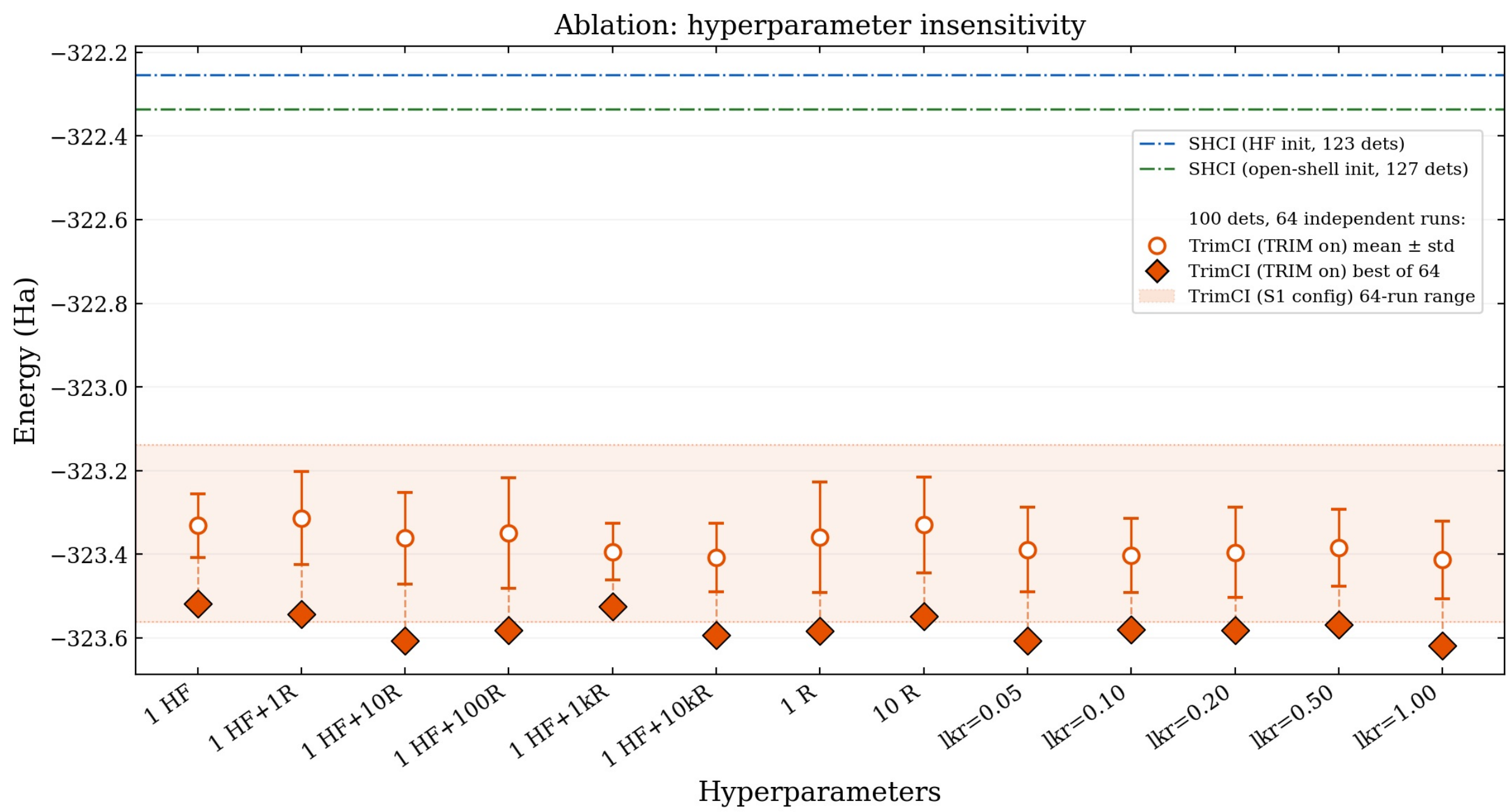

Suppl. Fig. 6. Ablation: hyperparameter insensitivity. Best energy (filled diamonds) and mean ± std (open circles with error bars) from 64 runs for each of 13 configurations. Left group: varying initial determinants (including two purely random). Right group: varying local trim keep ratio (lkr). All configurations produce similar results, and all outperform SHCI (dashed lines) by a wide margin. Source data are provided as a Source Data file.

Suppl. Fig. 6 shows that all 13 configurations achieve best energies in the range −323.5 to −323.6 Ha, with mean energies around −323.2 to −323.4 Ha. Neither the number of random initial determinants (including purely random, no-HF configurations), nor the aggressiveness of local trimming has a significant effect on the outcome. This insensitivity is a desirable property: it means that TrimCI does not require careful hyperparameter tuning for each new system, consistent with its design goal of requiring no problem-specific configuration.

Suppl. Note 4. PHYSICAL VALIDATION: WAVEFUNCTION ANALYSIS

A central question is whether TrimCI converges to the correct ground state or becomes trapped in a compact excited state. We address this through a detailed wavefunction analysis of Cr_2 at $R = 2.5\,\text{Å}$ (STO-3G basis, 48 electrons in 36 orbitals, HF canonical orbitals), where both TrimCI and SHCI are run to large determinant counts and their wavefunctions compared.

4.1. SHCI basin escape

SHCI is initialized from the Hartree–Fock determinant in HF canonical orbitals. Suppl. Fig. 7 shows the variational energy as a function of N_{dets} for both methods. SHCI exhibits a pronounced basin escape phenomenon:

- For $\varepsilon_1 \geq 0.001$ ($N_{\text{dets}} \leq 68{,}454$), SHCI remains in the **HF basin**: the HF determinant dominates with $|c_{\text{HF}}|^2 = 0.25$, and the other leading determinants are double excitations from HF ($|c| \sim 0.30$) and single excitations ($|c| \sim 0.23$).
- At $\varepsilon_1 = 9 \times 10^{-4}$ ($N_{\text{dets}} = 293{,}364$), SHCI undergoes a sudden transition: the energy drops by 0.29 Ha, the HF determinant disappears from the top-20, and the leading determinants become quadruple excitations with coefficients ~ 0.12.

In contrast, TrimCI immediately finds the multi-reference region at the 100-determinant scale. Its leading determinants are 4–6-fold excitations relative to HF, and the HF determinant does not appear among the leading determinants.

4.2. Leading determinant analysis

Suppl. Table 3 compares the leading determinants in three cases: SHCI before the basin escape ($\varepsilon_1 = 0.001$, 68K dets), SHCI after the escape ($\varepsilon_1 = 9 \times 10^{-4}$, 293K dets), and TrimCI (100 dets).

The physical content is the same: both methods, once past the HF basin, find wavefunctions dominated by excitations from the highest occupied MOs (19–23) into higher-lying virtual MOs. Suppl. Table 4 lists the relevant orbitals and their dominant atomic orbital

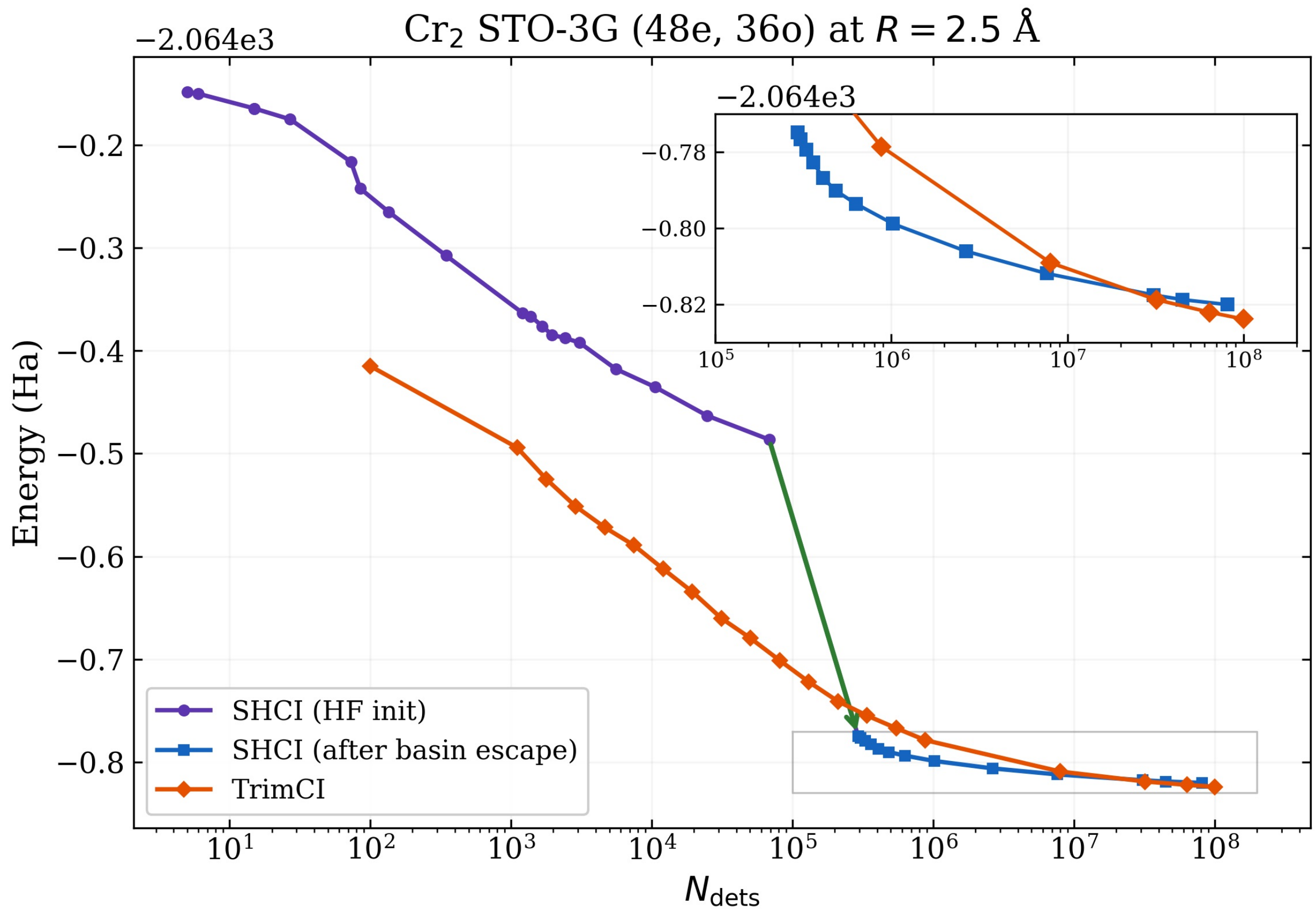

Suppl. Fig. 7. Energy convergence for Cr_2 STO-3G (48e, 36o) at $R = 2.5$ Å. SHCI (purple/blue) starts from HF and remains trapped in the HF basin until $\varepsilon_1 = 9 \times 10^{-4}$ (green arrow), when it undergoes a sudden 0.29 Ha energy drop. TrimCI (orange) starts from random determinants and converges smoothly from the multi-reference region. Inset: at N_{dets} = 30,920,784, TrimCI (−2064.8186 Ha) is 1.0 mHa below SHCI (−2064.8176 Ha). At 10^8 determinants, TrimCI reaches −2064.8239 Ha. Source data are provided as a Source Data file.

Suppl. Table 3. Leading determinants of Cr_2 in three cases. "Exc" denotes excitation level relative to HF. SHCI before escape is dominated by HF; after escape and TrimCI share the same 4-fold excitation pattern involving 3d bonding orbitals.

Stage	$\lvert c_{max} \rvert$	Exc	Dominant excitations
SHCI ($\varepsilon_1 = 0.001$)	0.500 (HF)	0–2	$19 \to 24$, $21 \to 25$
SHCI ($\varepsilon_1 = 9 \times 10^{-4}$)	0.123	4	$\{19, 20, 21, 22\} \to \{30, 31, 32, 33\}$
TrimCI (100 dets)	0.377	4–6	$\{19–23\} \to \{25, 26, 30–35\}$

(AO) character. The excitation pattern reflects the partial breaking of the Cr–Cr multiple bond at $R = 2.5\,\text{Å}$.

Suppl. Table 4. Molecular orbitals involved in the dominant excitations of Cr_2 STO-3G at $R = 2.5\,\text{Å}$. Cr_a and Cr_b denote the two Cr atoms; coefficients are the largest MO expansion weights on each atom.

MO	Energy (Ha)	Cr_a	Cr_b	Role
Occupied (electrons excited out of)				
19	−0.214	−0.69 (4s)	+0.70 (4s)	4s antibonding
20	−0.203	+0.63 ($3d_{z^2}$)	+0.61 ($3d_{z^2}$)	3d σ bonding
21	−0.119	+0.71 ($3d_{x^2\text{-}y^2}$)	+0.70 ($3d_{x^2\text{-}y^2}$)	3d δ bonding
22	−0.060	+0.50 ($4p_y$), +0.47 ($3d_{yz}$)	+0.50 ($4p_y$), −0.47 ($3d_{yz}$)	3d–4p hybrid
23	−0.060	+0.50 ($4p_x$), +0.47 ($3d_{xz}$)	+0.50 ($4p_x$), −0.47 ($3d_{xz}$)	3d–4p hybrid
Virtual (electrons excited into)				
24	+0.088	−0.70 ($3d_{x^2\text{-}y^2}$)	+0.71 ($3d_{x^2\text{-}y^2}$)	3d δ antibonding
25	+0.151	+0.55 ($3d_{z^2}$), −0.36 ($4p_z$)	−0.55 ($3d_{z^2}$), −0.37 ($4p_z$)	3d σ antibonding
26	+0.262	+0.70 ($3d_{yz}$)	+0.71 ($3d_{yz}$)	3d π
27	+0.262	+0.71 ($3d_{xz}$)	+0.71 ($3d_{xz}$)	3d π
30	+0.294	+0.53 ($3d_{xz}$), −0.48 ($4p_x$)	−0.52 ($3d_{xz}$), −0.48 ($4p_x$)	3d–4p hybrid
31	+0.329	+0.83 ($4p_x$)	−0.83 ($4p_x$)	4p
32	+0.329	+0.83 ($4p_y$)	−0.83 ($4p_y$)	4p
33	+0.351	+0.61 ($3d_{xy}$)	+0.78 ($3d_{xy}$)	3d δ

4.3. Orbital occupation and 3d bond density

We compute the one-particle reduced density matrix (1-RDM) and visualize the 3d electron density in Suppl. Fig. 8.

The five MOs with dominant 3d character (MO 20, 21, 25, 26, 33; see Suppl. Table 4 for AO composition) show a clear transition:

- **HF / SHCI (HF basin)**: Only MO 20 and 21 are occupied (4 electrons in 2 bonding

MOs), producing density connecting the two Cr atoms.

- **SHCI (after escape) / TrimCI**: Electrons redistribute across all five 3d MOs, producing a qualitatively different density pattern reflecting the partial bond breaking.

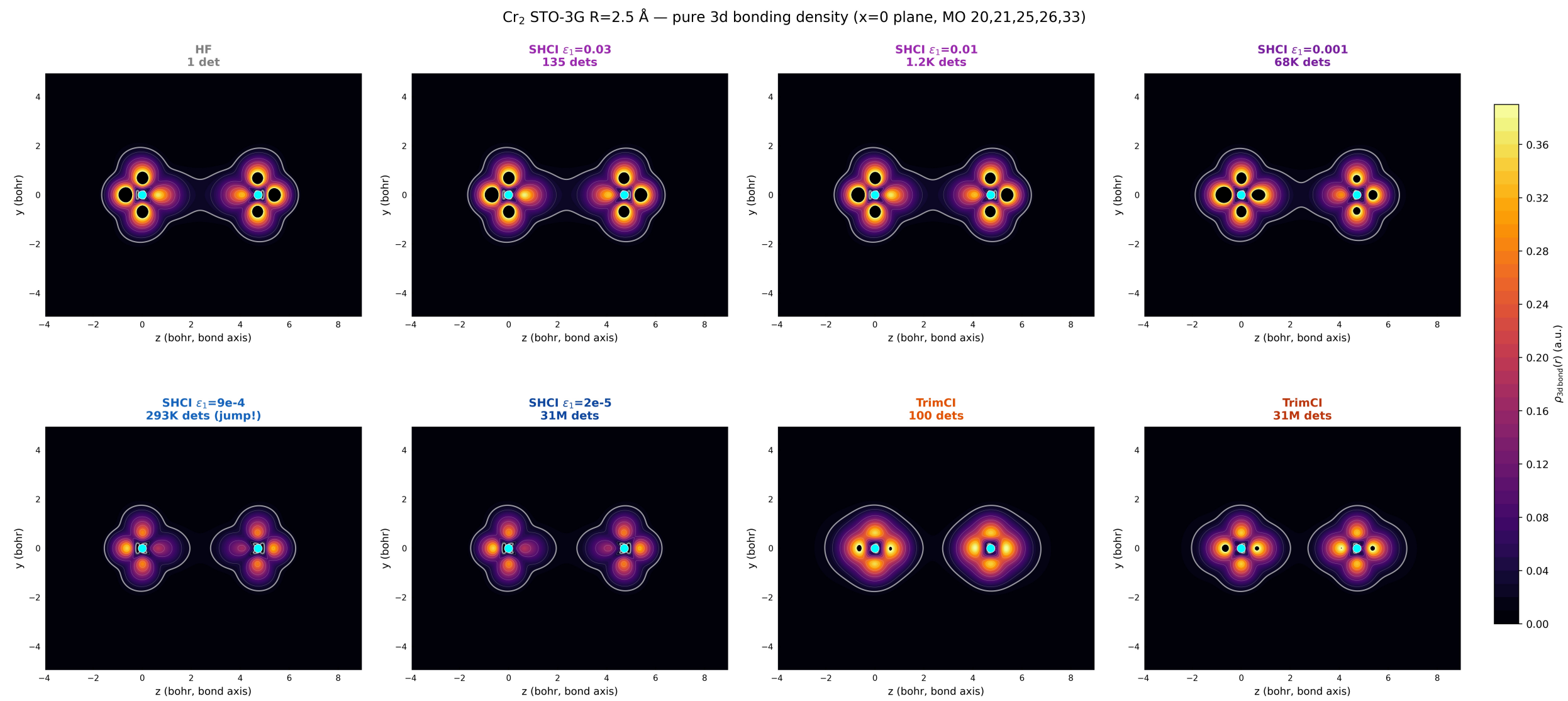

Suppl. Fig. 8. 3d electron density for Cr_2 in eight cases (MO 20, 21, 25, 26, 33; $x = 0$ cross-section). Top row: HF → SHCI with decreasing ε_1 (135, 1.2K, 68K dets), all within the HF basin. Bottom row: SHCI after basin escape (293K dets) → SHCI at 31M dets → TrimCI (100 dets) → TrimCI (31M dets). The transition from bonding density connecting atoms (top row) to redistributed density (bottom left) occurs at the SHCI basin escape; TrimCI captures this physics from the start at 100 dets.

4.4. Convergence comparison

At exactly $N_{\text{dets}} = 30{,}920{,}784$ (matching SHCI's $\varepsilon_1 = 2 \times 10^{-5}$), TrimCI achieves $E_{\text{var}} = -2064.8186$ Ha vs. SHCI's -2064.8176 Ha—a 1.0 mHa advantage. SHCI requires 45.1×10^6 determinants ($\varepsilon_1 = 1.5 \times 10^{-5}$) to reach the same energy (-2064.8187 Ha), representing 46% more determinants.

The convergence advantage is not due to warm-start bias in Davidson: a control experiment with `no_warm_start=True` (random initial guess at each expansion round) produces identical energies (difference < 0.01 mHa at all sizes).

These results directly address the concern about excited-state trapping. SHCI is initially trapped in the HF basin and only escapes at $\sim 3 \times 10^5$ determinants, after which it finds a wavefunction with the same leading determinant structure and 3d orbital occupation as TrimCI. TrimCI reaches this multi-reference region immediately after the initial core search. At 3×10^7 determinants, TrimCI remains 1.0 mHa lower in energy than SHCI.

Suppl. Note 5. PHYSICAL VALIDATION: HUBBARD MODEL

We further validate TrimCI on the half-filled Hubbard model on a 4×4 square lattice with periodic boundary conditions ($U/t = 2$, 16 electrons in 16 orbitals), where the exact FCI solution is available ($E_{\mathrm{FCI}} = -18.0176\, t$, where t is the hopping amplitude). Both SHCI and TrimCI use HF canonical orbitals (the standard choice for Hubbard model calculations in the determinant basis), and SHCI is initialized from the HF determinant—a fair comparison. This system provides a stringent test because (i) it is strongly correlated, (ii) the FCI ground state has significant antiferromagnetic (AFM) order, and (iii) observables can be computed exactly for comparison.

5.1. Energy and AFM order parameter

Suppl. Fig. 9 compares the relative error in energy and in the AFM spin structure factor $S_{zz}(\pi, \pi)/N = \frac{1}{N} \sum_{ij} (-1)^{x_i+y_i+x_j+y_j} \langle S_z^i S_z^j \rangle$ as a function of N_{dets}. The AFM order parameter is computed from the two-particle reduced density matrix (2-RDM), transformed from MO to site basis using the orbital coefficient matrix.

Two features stand out:

1. **SHCI is trapped in the HF basin for observables.** While SHCI's energy error decreases slowly from 5.5% to 4.6% over 4,592 to 1,203,472 determinants, its AFM error remains essentially flat at $\sim$ 28% throughout. This means that even at 1.2×10^6 determinants, the SHCI wavefunction (initialized from HF) does not capture the correct antiferromagnetic correlations—a direct physical consequence of being trapped in the HF basin.

2. **TrimCI captures the correct physics.** TrimCI's energy error drops to 0.05% and its AFM error to 2.3% at 1.8×10^5 determinants. While the AFM error is relatively

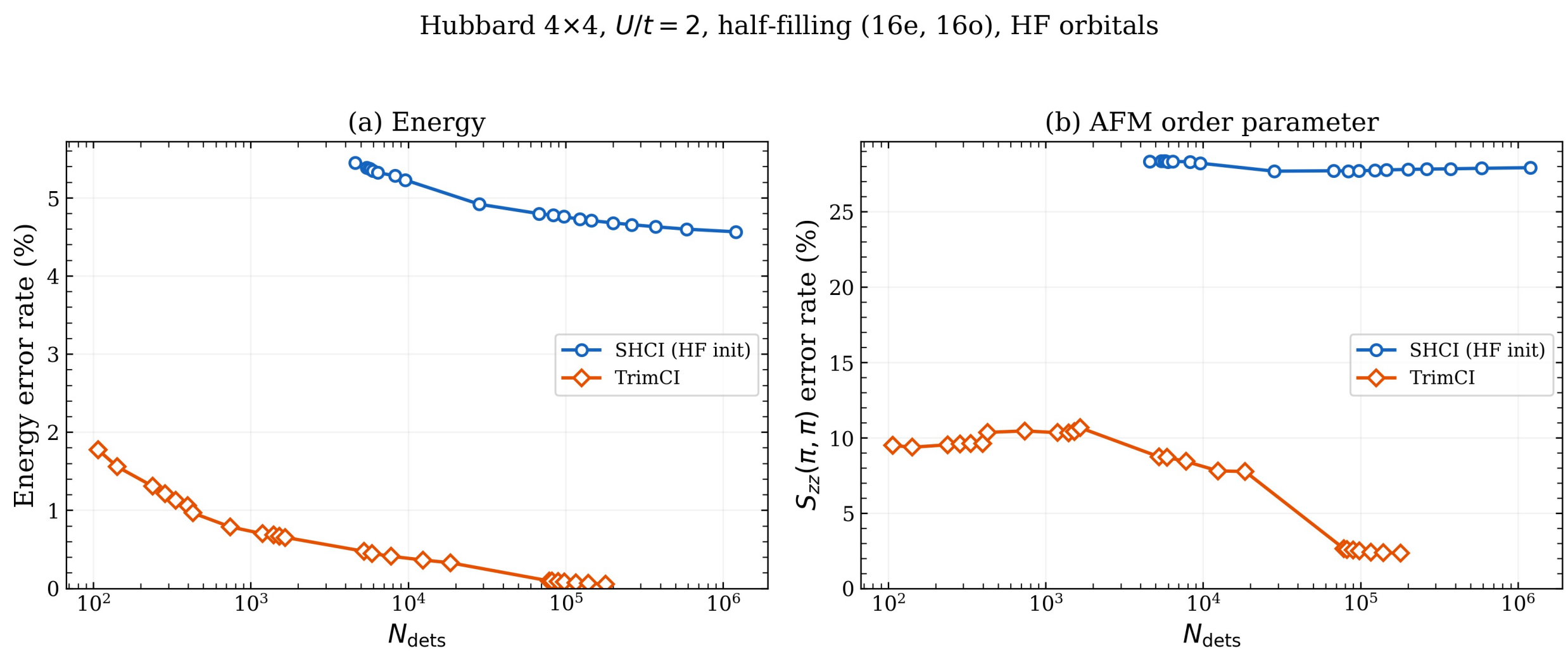

Suppl. Fig. 9. Hubbard 4×4, $U/t = 2$, half-filling, HF orbitals. Relative error vs. FCI in (a) variational energy and (b) AFM order parameter $S_{zz}(\pi, \pi)/N$. SHCI (blue) initialized from HF shows energy error $\sim 5\%$ and AFM error $\sim 28\%$, neither of which improves appreciably with N_{dets}. TrimCI (orange) converges to $< 0.1\%$ energy error and $\sim 2\%$ AFM error at 1.8×10^5 determinants. Source data are provided as a Source Data file.

larger than the energy error—reflecting the greater sensitivity of spin correlations to wavefunction fidelity—it remains small and correctly reproduces the antiferromagnetic order.

This result complements the Cr_2 analysis (S4): SHCI's HF basin trapping affects not only the energy but also physical observables. TrimCI, through its trimming mechanism, converges to the correct ground-state physics, both in the energy and in the spin correlations.

Suppl. Note 6. CR$_2$ CC-PVDZ-DK: LARGE-BASIS BENCHMARK

We test TrimCI on the notoriously difficult chromium dimer Cr_2 at $R = 2.50\,\text{Å}$ (the "shelf" region of the potential energy curve) using the cc-pVDZ-DK basis. The active space CAS(28e, 76o) correlates 28 electrons in 76 orbitals. This is the same system studied by Larsson et al. [5] with SHCI and DMRG.

6.1. Hamiltonian construction

We follow the same electronic structure setup as Larsson et al. [5]: the spin-free exact two-component (SFX2C1e) scalar relativistic Hamiltonian with cc-pVDZ-DK basis functions. Each Cr atom contributes 43 spatial AOs (86 total for the dimer). Two neon cores (10 spatial orbitals, 20 electrons) are frozen, yielding an active space of 28 electrons in 76 orbitals.

The FCIDUMP is generated via PySCF as follows:

1. X2C-RHF reference: `scf.RHF(mol).sfx2c1e()` with `conv_tol=`10^{-10}.

2. CASSCF(12,12) orbital optimization: a complete active space of 12 electrons in the 3d+4s shell (12 orbitals) is used to generate orbitals that capture the dominant strong correlation. The CASSCF optimizes *all* orbitals (core, active, virtual), producing a set of molecular orbitals that improve convergence of the subsequent large-scale CI.

3. Frozen-core integral transformation: one- and two-electron integrals are transformed to the CASSCF MO basis, then an effective active-space Hamiltonian is constructed by folding the frozen-core potential into the one-electron integrals and absorbing the frozen-core energy into the nuclear repulsion constant ($E_{\text{core}} = E_{\text{nuc}} + E_{\text{frozen}}$).

This procedure produces a standard FCIDUMP file with NORB = 76, NELEC = 28, and MS2 = 0.

6.2. TrimCI workflow

The TrimCI calculation proceeds in three phases:

1. **Phase 0** (core search with orbital optimization, 10K dets): 64 independent runs, each starting from 1 HF + 10,000 random determinants. `first_cycle_keep_size` = 10, `max_rounds` = 4, `max_final_dets` = 10,000, `pool_core_ratio` = 40, `num_groups` = 20, `local_trim_keep_ratio` = 4, `core_set_ratio` = [1, 1.1], `threshold` = 10^{-2}. BFGS orbital optimization (10 cycles) is enabled. Final energy: -2099.618 Ha at 10,512 dets.

2. **Phase 1** (expansion to 1M): Growth factor 1.1, orbital optimization enabled, 47 expansion rounds.

3. **Phase 2** (expansion to 100M + PT2): Growth factor 2.0, 7 rounds from 2M to 100M dets with Epstein–Nesbet PT2 at each step.

All computations were performed on a single node with two AMD EPYC 9254 processors (48 physical cores, 96 threads) and 1 TB RAM. Total wall time for the complete workflow: 40.2 hours.

6.3. Results and comparison with published SHCI

We compare TrimCI with the best published SHCI results from Larsson et al. [5]. Suppl. Fig. 10 shows the energy convergence. TrimCI's variational energy crosses the SHCI best E_{var} at $\sim 4 \times 10^6$ determinants, and the PT2-corrected energy crosses both the SHCI best E_{var}+PT2 and the DMRG ($D = 18{,}000$) at $\sim 2 \times 10^6$ determinants.

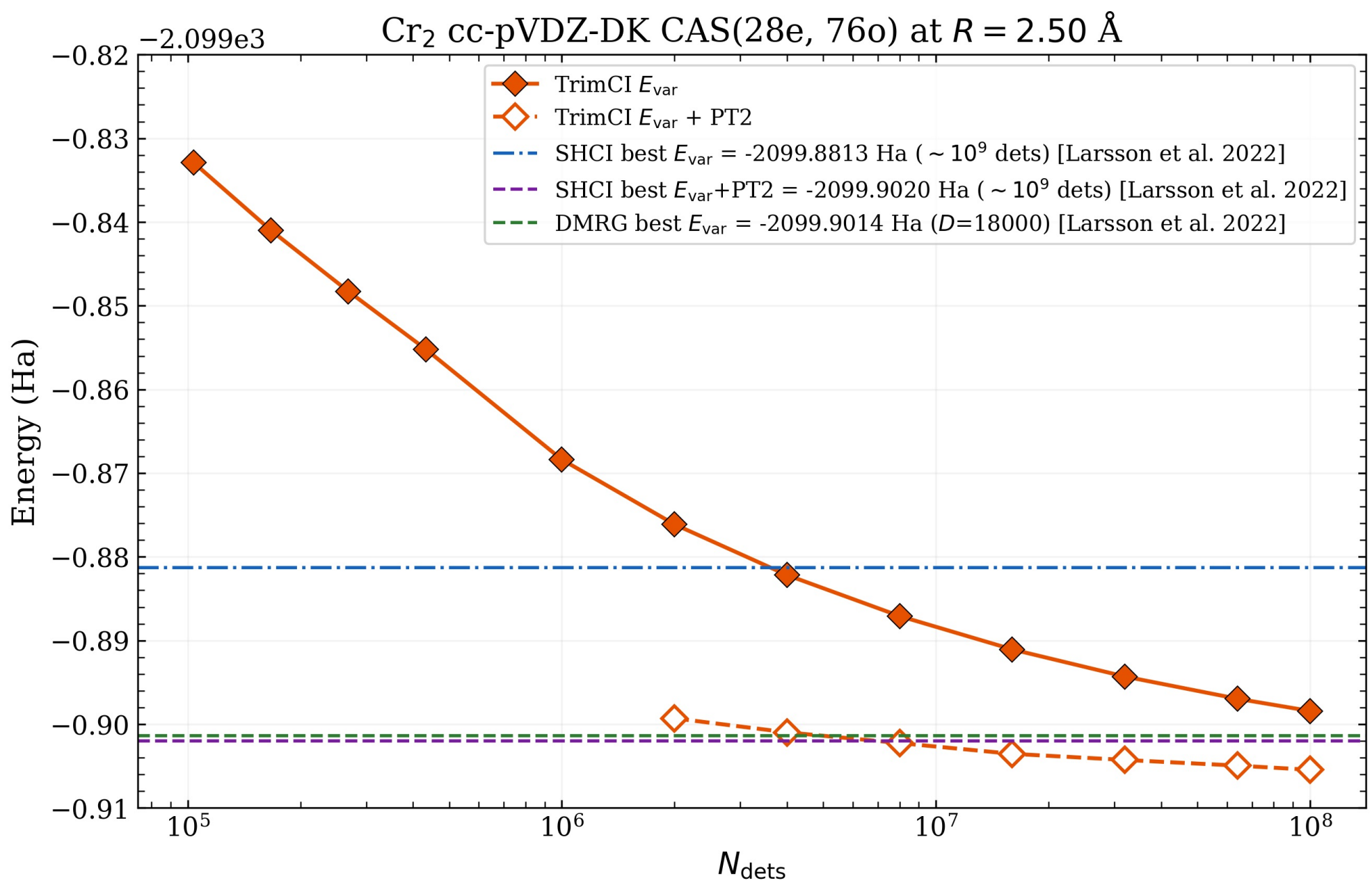

Suppl. Fig. 10. Energy convergence for Cr_2 cc-pVDZ-DK CAS(28e, 76o) at $R = 2.50$ Å. TrimCI E_{var} (filled diamonds) and E_{var}+PT2 (open diamonds) compared with the best published SHCI and DMRG results from Larsson et al. [5]. Source data are provided as a Source Data file.

At 10^8 determinants, TrimCI achieves $E_{\mathrm{var}} = -2099.8984$ Ha and E_{var}+PT2 $= -2099.9054$ Ha. Linear PT2 extrapolation and R^2-scan power-law extrapolation both yield $E_{\mathrm{extrap}} \approx -2099.9081$ Ha, within 1.5 mHa of the SHCI/DMRG extrapolated reference (-2099.9096 Ha).

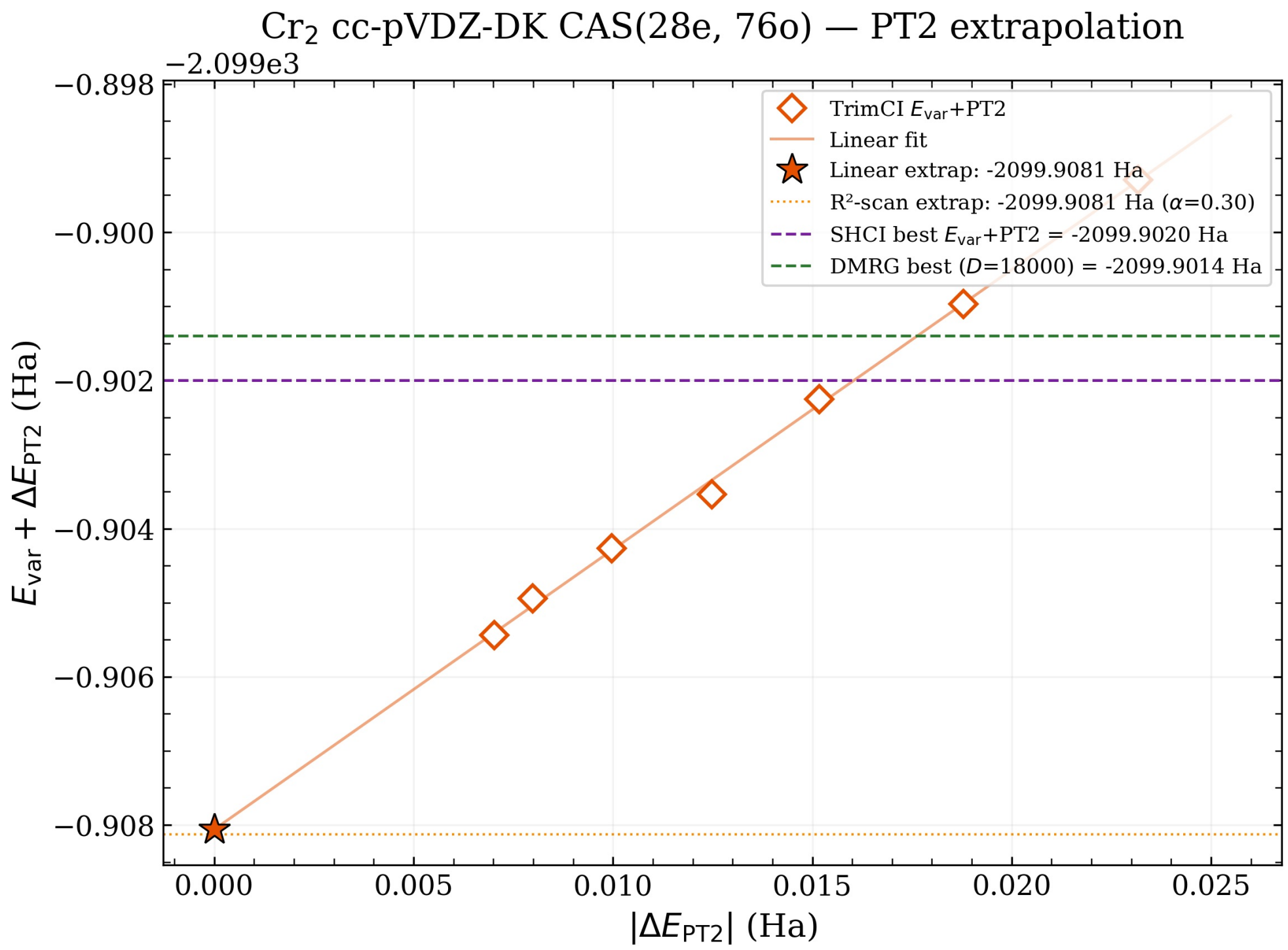

Suppl. Fig. 11. PT2 extrapolation for Cr_2 cc-pVDZ-DK CAS(28e, 76o). $E_{\mathrm{var}} + \Delta E_{\mathrm{PT2}}$ vs. N_{dets} with R^2-scan power-law fit ($\alpha = 0.30$, $\mathrm{R}^2 = 0.999$) and linear PT2 extrapolation. Both methods give $E_{\mathrm{extrap}} \approx -2099.9081$ Ha. Source data are provided as a Source Data file.

In summary, TrimCI achieves E_{var} **17 mHa lower**, E_{var}+PT2 **3.4 mHa lower**, and E_{extrap} within **1.5 mHa** of the SHCI/DMRG reference. Suppl. Table 5 provides hardware and timing details: based on the timing information available from the literature, TrimCI uses $\sim 6\times$ **fewer core-hours** on a single commodity node.

These results demonstrate that TrimCI achieves state-of-the-art accuracy for strongly correlated systems in large basis sets, with far fewer computational resources on a single commodity node.

Suppl. Note 7. RECOVERING FROM RANDOM ORBITALS

The main text and preceding sections have shown that TrimCI can find a compact wavefunction across diverse strongly correlated systems (molecular systems and the Hubbard model), with orders of magnitude fewer determinants than SHCI. The wavefunction analyses in Suppl. Note 4 and Suppl. Note 5 further established that this compact wavefunc-

Suppl. Table 5. Hardware and timing comparison for Cr_2 cc-pVDZ-DK CAS(28e, 76o) at $R = 2.50$ Å. Energies in Ha (shifted by 2099). SHCI data from Larsson et al. [5]. SHCI timing is reported for $R = 2.25$ Å (the most computationally demanding bond length); $R = 2.50$ Å may require somewhat less time.

Method	E_{var}	E_{var}+PT2	E_{extrap}	Hardware	Wall time	Core-hours
SHCI[a]	−0.881	−0.902	−0.910	2 nodes, 160 cores (Xeon E7-8880)	∼75 h[b] var 60 h + PT2 15 h	∼ 12,000[b]
**TrimCI**	**−0.898**	**−0.905**	**−0.908**	**1 node, 48 cores (2×EPYC 9254)**	**40.2 h**	**1,930**

[a] Larsson et al., JACS 2022. [b] Timing for $R = 2.25$ Å: var 60 h on 2×80 cores + PT2 15 h on 80 cores.

tion captures the correct ground-state physics: bond-breaking patterns, leading-determinant character, occupation profiles, and order parameters all match independent references. Here we turn to a question on the dependence of the TrimCI results on the orbital basis.

Starting from scrambled orbitals, can TrimCI find a good orbital basis on its own, one in which the convergence behavior permits extrapolation to FCI? In Suppl. Note 1, we already constructed five scrambled FCIDUMP files by applying independent random orthogonal rotations $U^{(r)} \in O(36)$ to the one- and two-electron integrals of the $[Fe_4S_4]$ CAS(36o, 54e) Hamiltonian (`rot_01`–`rot_05`); the eigenvalues of each rotated Hamiltonian are preserved exactly, but the orbital structure is randomized, leaving no localized or physically meaningful orbitals and making the variational problem strictly harder. Here we use those same five rotated Hamiltonians as a stress test. If the compact TrimCI wavefunction reflects genuine ground-state physics, then the orbital gradient it carries should be sufficient to rotate the random basis back to one in which the variational expansion can be carried to a near-FCI limit.

7.1. TrimCI rotates random orbitals back to a basis supporting FCI extrapolation

The complete TrimCI workflow we use for production-level ground-state calculations runs in three phases. The blackbox benchmark in Suppl. Note Suppl. Note 1 and the comparison in the main text deliberately disabled the orbital optimization to isolate the

direct comparison against SHCI: given the same orbital basis, can TrimCI find a more compact subspace than SHCI does? The present section instead restores the full workflow with orbital optimization on each of the five scrambled FCIDUMPs. *Phase 0* is a global search stage: an ensemble of 64 independent searches each starts from a random determinant pool, iteratively expands and trims it to a 100-determinant core wavefunction, computes the orbital gradient from the resulting 1- and 2-RDM, and applies a BFGS procedure to rotate the orbital basis; ten such cycles run before the workflow hands off to the expansion stage. *Phases 1 and 2* together form the expansion stage that grows the wavefunction from ∼100 to 10^7–10^8 determinants or more. Phase 1 (∼100 to ∼10^6 determinants) continues to refine the orbitals at every expansion; Phase 2 freezes the orbital basis, expands rapidly to 10^7–10^8 determinants, and optionally adds an E_{PT2} correction at each expansion.

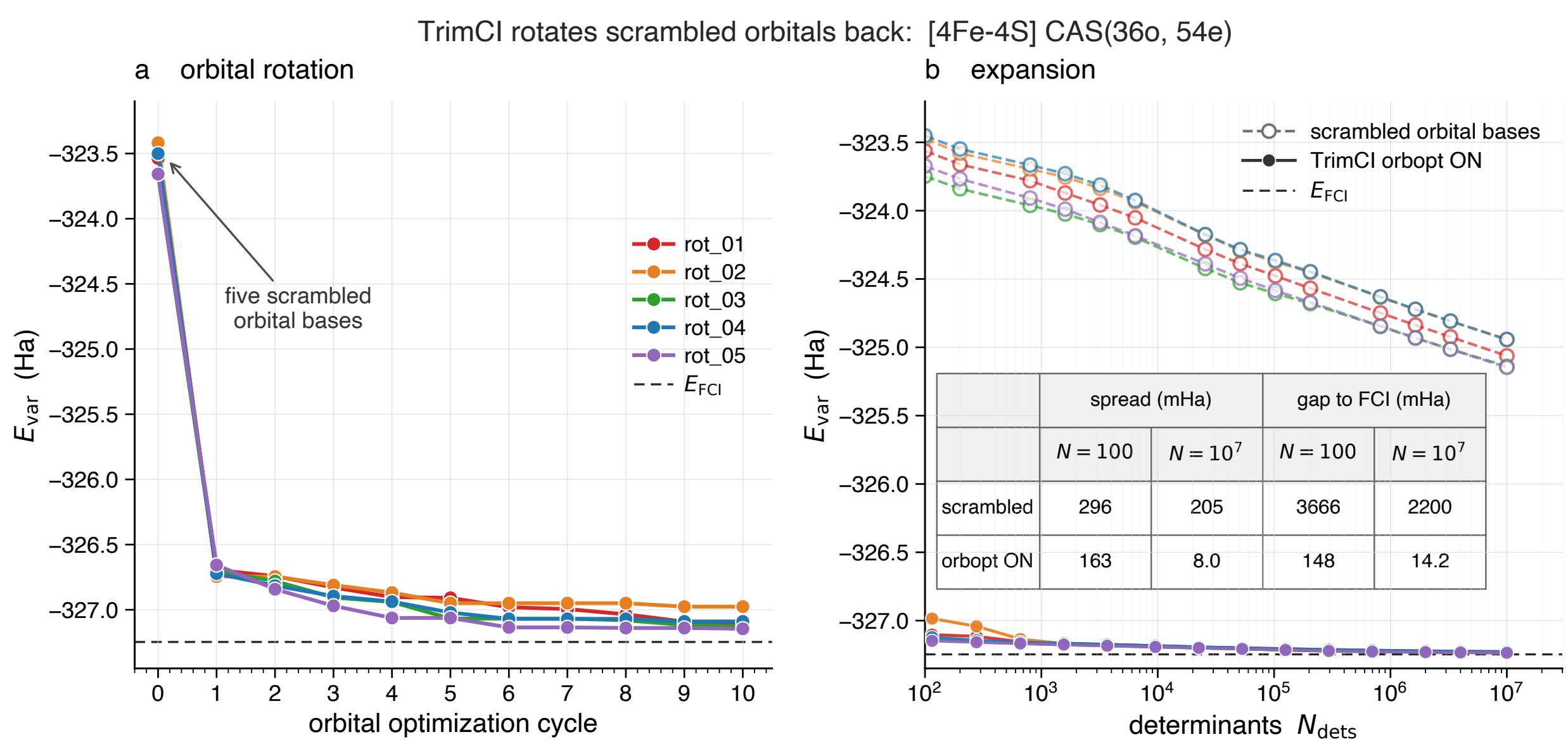

Suppl. Fig. 12. TrimCI rotates scrambled [Fe_4S_4] orbitals back. **(a)** Cumulative-best E_{var} over the 10 Phase 0 TrimCI orbital-optimization cycles (100 determinants per cycle) for each of the five random orbitals. **(b)** Phase 1+2 expansion: filled markers, optimized basis; hollow markers with dashed lines, unrotated scrambled basis (orbital optimization disabled). The inset table reports, at $N_{\mathrm{dets}} = 100$ and 10^7, the *spread* (max−min of E_{var} across the five rotations) and the *gap to the broken-symmetry FCI manifold* (mean of the five E_{var} values minus the UDMRG broken-symmetry FCI range −327.244 to −327.250 Ha [6]). Source data are provided as a Source Data file.

Suppl. Fig. 12 reports the results on the scrambled orbitals. Panel (a) shows that the

cycle-0 core wavefunction on the scrambled basis sits near −323.5 Ha across all five scrambled orbitals, and within ten TrimCI orbital-optimization cycles drops to the range −326.97 to −327.15 Ha, a ∼3.5 Ha reduction obtained from 100-determinant TrimCI wavefunctions. Despite starting from scrambled orbitals, TrimCI finds, on each of the five Hamiltonians, a compact wavefunction that carries a useful orbital gradient, allowing the basis to be rotated back to one well suited for the ground state. Panel (b) shows the subsequent Phase 1+2 expansion. Starting from the orbital basis chosen by Phase 0, all five trajectories converge to within 8 mHa of one another at 10^7 determinants: the variational energies span −327.228 to −327.236 Ha and the PT2-corrected energies span −327.232 to −327.239 Ha. Linear and quadratic $E_{\mathrm{var}} + E_{\mathrm{PT2}}$ vs $|E_{\mathrm{PT2}}|$ extrapolations of the five trajectories place the TrimCI ground-state estimate in the range −327.240 to −327.249 Ha. For comparison, UDMRG [6] reports the three broken-symmetry FCI states of $[Fe_4S_4]$ in the range −327.244 to −327.250 Ha; the two ranges overlap closely, indicating that TrimCI from the scrambled basis converges to the manifold of broken-symmetry FCI states. The dashed curves in panel (b) show the expansion run on the original scrambled basis (the workflow used in the blackbox benchmark of Suppl. Note Suppl. Note 1, with orbital optimization disabled). In production runs, then, TrimCI starts from a randomly scrambled orbital basis and converges to an orbital basis on which the variational expansion supports an FCI extrapolation. The key observation is that, although the five scrambled orbital bases are very different from each other, the variational energies that TrimCI converges to differ by only ∼ 8 mHa, illustrating the stability of the production workflow.

Suppl. Table 6 reports the mean and standard deviation of E_{var} across the five scrambled orbitals: the mean descends from −323.556 to −327.233 Ha and the standard deviation across the five scrambled problems shrinks from 105.5 to 4.4 mHa as N_{det} grows from 100 (before orbital optimization) to 10^7. The final ∼ 5 mHa spread reflects the spread of the three broken-symmetry FCI states of $[Fe_4S_4]$ [6].

Suppl. Table 6. Mean and standard deviation of E_{var} across the five scrambled orbitals of $[Fe_4S_4]$ as N_{det} grows. The first two rows are the 100-determinant core at the start (cycle 0) and end (cycle 10) of Phase 0 orbital optimization; the remaining rows are the Phase 1+2 expansion on the optimized basis.

N_{det}	mean E_{var} (Ha)	std (mHa)
100 (before orbopt)	−323.556	105.5
100 (after orbopt)	−327.069	87.5
120	−327.099	65.8
150	−327.103	65.3
200	−327.113	55.1
500	−327.145	29.0
1000	−327.164	7.4
10^7	−327.233	4.4

In fact, the original LMO basis itself is not optimal for the ground state. In the main text the comparison is carried out in a fixed LMO basis (the same FCIDUMP across methods), so the quantity reported there is how compact the TrimCI wavefunction is in that basis relative to other methods. Here, by turning on orbital optimization, the same workflow reaches a substantially lower energy. Pushing the same three-phase workflow another two orders of magnitude in N_{dets} would tighten the extrapolation range further; we do not intend to extend each of the five rotated cases that far due to resource considerations. We did, however, run a similar workflow on BS1 of $[Fe_4S_4]$, reaching $N_{\text{det}} = 5.12 \times 10^9$ determinants. The extrapolated energy is $E = -327.2441$ Ha, only ~ 0.2 mHa from the UDMRG extrapolated energy of -327.2443 Ha [6]. We report the details of the 2.56×10^9-determinant single-node calculation and its full convergence trajectory in Suppl. Note 8.1. Other details about TrimCI on the iron-sulfur series are reported in Ref. [7].

7.2. Visualizing the recovered basis

To visualize the recovery, we expand each rotated basis in terms of the 36 original $[Fe_4S_4]$ localized orbitals. For rotation r ($r = 1, \ldots, 5$) and orbital i in the current basis, we

plot $|\psi_i(j)|^2 = |C^{(r)}_{ji}|^2$, where $C^{(r)}$ is the cumulative rotation matrix taking LMO indices to the current basis. At cycle 0 the basis is the scrambled one and $C^{(r)} = V^{(r)}$. The orbital-optimization update $U^{(r)}_{\text{orbopt}}$ then further rotates the basis within itself, giving $C^{(r)} = V^{(r)} \cdot U^{(r)}_{\text{orbopt}}$, with the convention $|i\rangle_{\text{new}} = \sum_j |j\rangle_{\text{LMO}}\, C^{(r)}_{ji}$.

Suppl. Fig. 13 shows this evolution under a coarse three-tier colormap that emphasizes structural change rather than per-element magnitude: white when $|C_{ji}|^2 < 10^{-2}$ (the orbital essentially does not live on LMO j), green when $10^{-2} \leq |C_{ji}|^2 < 10^{-1}$ (moderate weight), gold when $|C_{ji}|^2 \geq 10^{-1}$ (dominant weight). Within each panel, the orbitals are sorted by the mean LMO index of their density (the index j averaged with weight $|C_{ji}|^2$), so orbitals concentrated on neighboring LMOs sit next to each other.

Across all five rotations the pattern is the same. Cycle 0 fills the panel with a uniformly random green background, because every random orbital has roughly equal $\sim 1/36$ weight on every LMO; a small fraction of cells already sits above the 10^{-1} threshold but at uncorrelated positions across orbitals. By cycle 2 the green band thins out as its weight flows into the gold cells, which become heavier. By cycle 10 the panel is dominated by white background with sharp gold landmarks: each recovered orbital concentrates onto a small handful of original LMOs. Quantitatively, the random basis delocalizes each orbital uniformly: 67% of its density sits in the moderate-weight green band ($|C_{ji}|^2 \in [10^{-2}, 10^{-1}]$) and 28% in the dominant gold cells. TrimCI's orbital gradient relocalizes the orbital — at cycle 10, density has flowed from green into gold resulting in 23% and 73% weight respectively. The number of gold cells stays near the same throughout, but their relative positions migrate during optimization, reflecting the entanglement pattern of $[Fe_4S_4]$. Despite starting from random orbitals, TrimCI recovers meaningful orbitals with intricate sparse structure.

7.3. Comparison with SHCI orbital optimization

A natural question is whether other orbital optimization methods recover the same way on the same stress test. We compare against SHCI's orbital optimization, run on the same five rotated FCIDUMPs with a typical orbopt config of 10 orbital-optimization cycles at $\varepsilon_{\text{var}} = 10^{-3}$ (inner-CI size $\sim$ 600,000 determinants per cycle).

Suppl. Fig. 14 shows the side-by-side orbital comparisons. The leftmost column is the scrambled basis ($V^{(r)}$), the middle column the SHCI-optimized basis ($V^{(r)} \cdot U^{(r)}_{\text{SHCI}}$), and

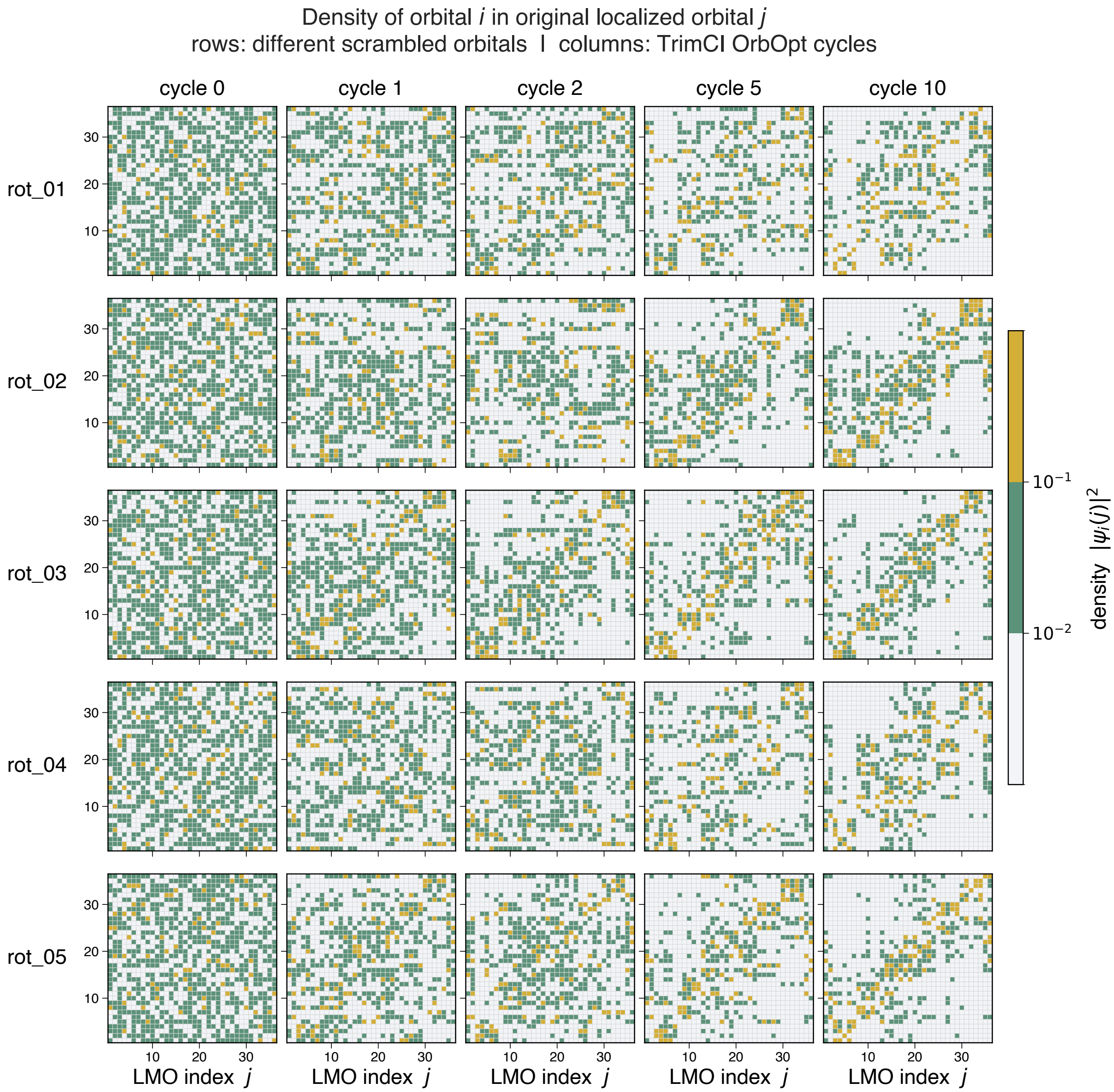

Suppl. Fig. 13. Density of orbital i in original localized orbital j as a function of TrimCI Phase 0 cycle, for each of the five random rotations (rows: `rot_01`–`rot_05`; columns: cycle 0, 1, 2, 5, 10). Each cell is colored by its raw matrix element $|C_{ji}|^2$ in three tiers: white ($< 10^{-2}$, the orbital essentially does not live there), green (10^{-2}–10^{-1}, moderate weight), gold ($\geq 10^{-1}$, dominant weight). Cycle 0 shows the random scrambled basis: every orbital is a uniform mixture and most cells are green. As cycles progress, the green band depletes as density flows into the gold cells, sharpening each orbital's localization on its principal LMOs. The same pattern holds across all five scrambled orbitals.

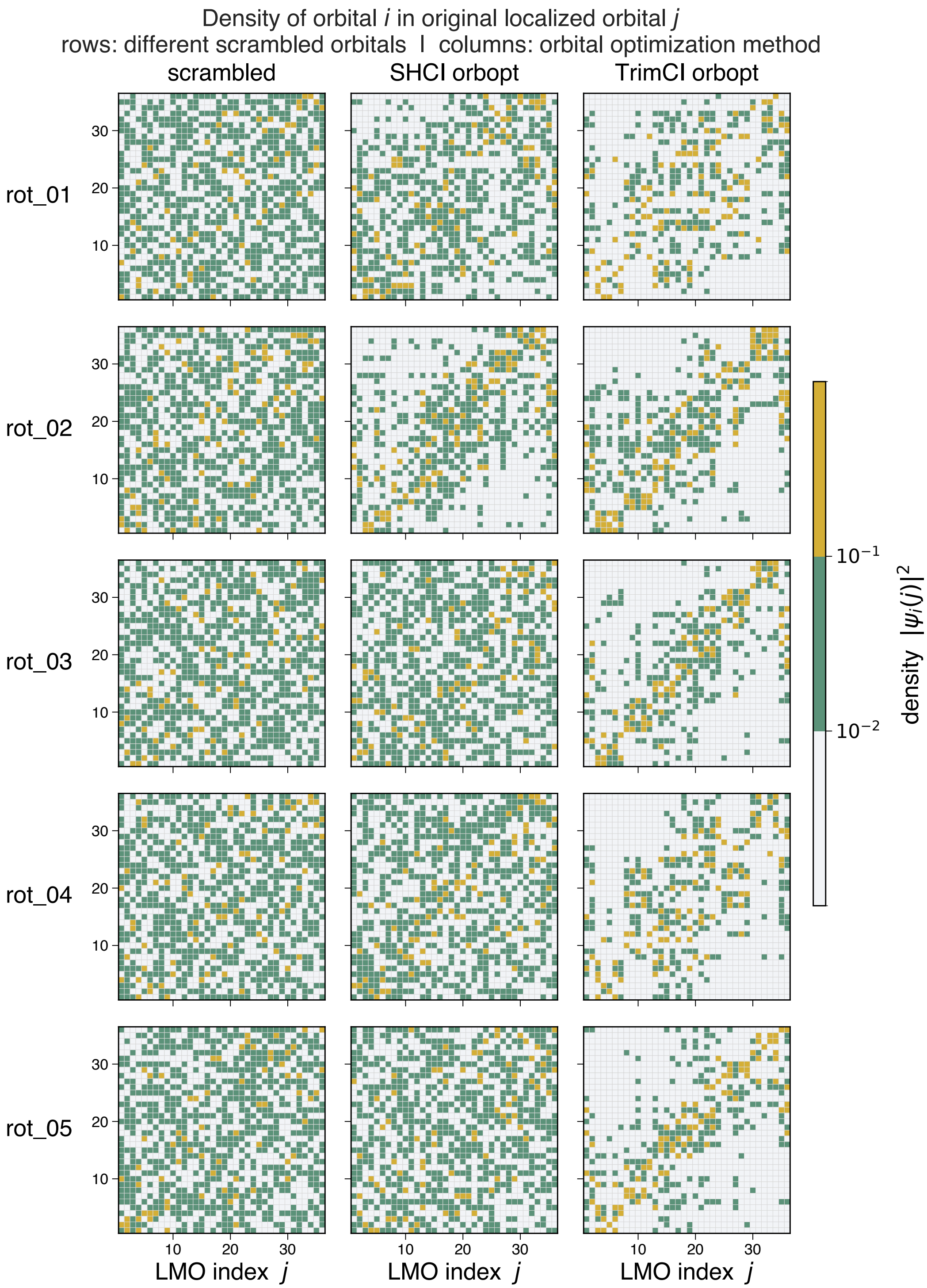

Suppl. Fig. 14. Recovered orbital basis after orbital optimization, for SHCI vs. TrimCI on the same five random orbitals. Each row is a problem; columns are (left to right): the original scrambled basis, the basis after SHCI orbital optimization, and the basis after TrimCI orbital optimization. Color coding as in Suppl. Fig. 13. TrimCI consistently produces a sparser, more concentrated basis on every problem; SHCI's recovery is weaker and problem-dependent (`rot_03`–`rot_05` are essentially unchanged from scrambled).

the rightmost the TrimCI-optimized basis ($V^{(r)} \cdot U^{(r)}_{\text{TrimCI}}$). Across all five orbitals, TrimCI produces visibly sparser distributions with large fractions of white space and clearly-defined gold landmarks. SHCI's recovery is partial and problem-dependent: `rot_01` and `rot_02` show some concentration, while `rot_03`, `rot_04`, and `rot_05` remain close to the scrambled column. The gold-cell weight confirms this quantitatively. For problems rot_01/02/03/04/05, the gold weight is 71/70/73/74/76% for TrimCI (right column), 46/60/36/36/32% for SHCI (middle column), and 26/29/27/30/27% for the scrambled basis (left column). TrimCI achieves substantial orbital concentration on every problem, far surpassing SHCI.

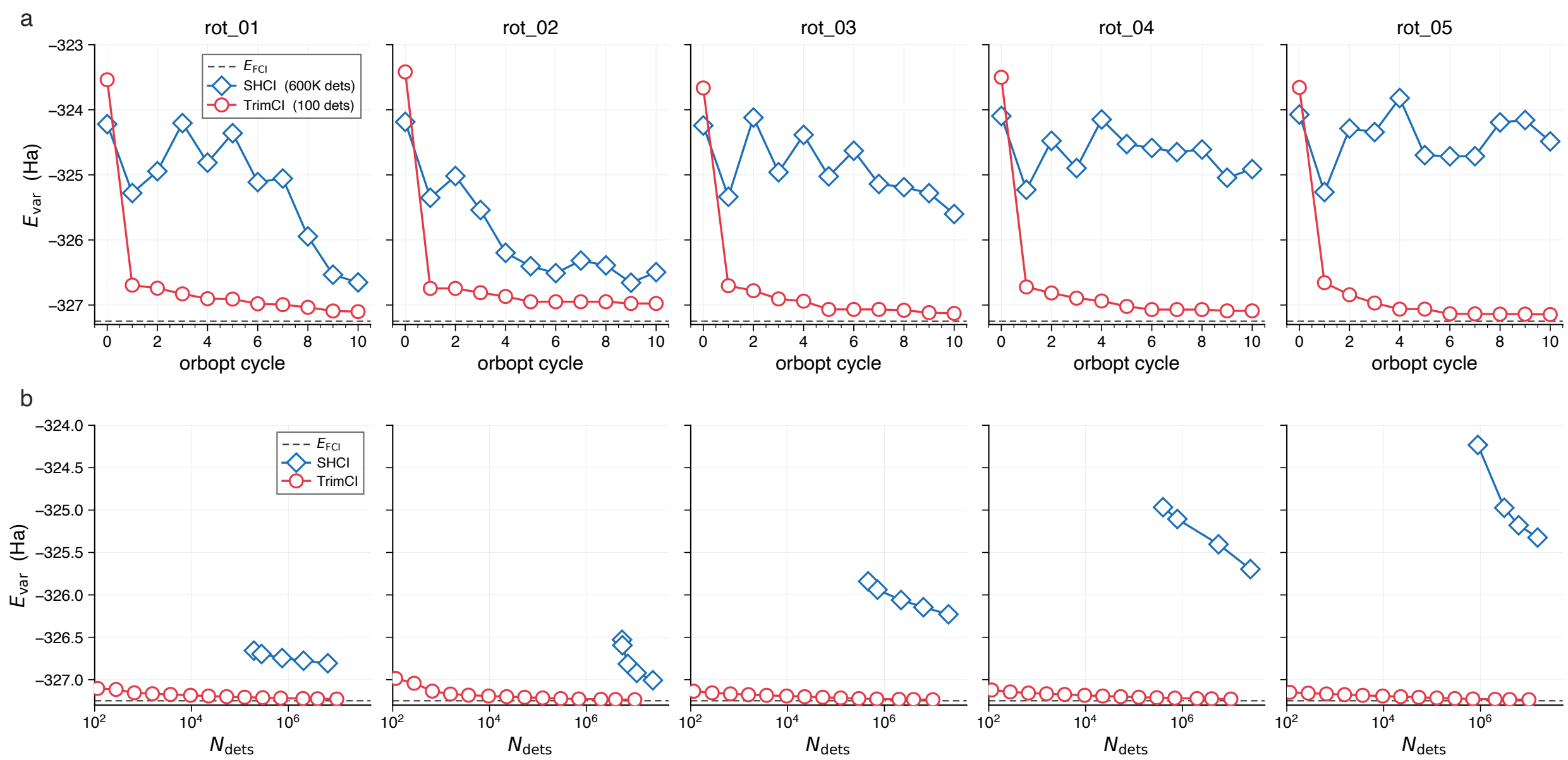

Suppl. Fig. 15. Energy trajectories during **(a)** orbital optimization and **(b)** subsequent expansion, for the same five random orbitals, comparing SHCI orbital optimization (blue, $\sim$ 600,000 determinants per inner CI) to TrimCI orbital optimization (red, 100 determinants). **(a)** SHCI orbital optimization oscillates up and down between adjacent cycles, and `rot_03`–`rot_05` show almost no improvement over 10 cycles; TrimCI's orbital optimization descends fast and monotonically. **(b)** After orbital optimization, each method expands on its optimized basis. TrimCI's expansion approaches E_{FCI} across all five problems; SHCI's converges much more slowly, stopping at -325 to -327 Ha at the largest determinant counts and missing E_{FCI} by hundreds to thousands of mHa. Source data are provided as a Source Data file.

Suppl. Fig. 15 compares the energy trajectories. Panel (a) shows the per-cycle inner-CI variational energy during orbital optimization. SHCI's steps oscillate up and down through-

out the 10 cycles, while TrimCI's trajectory is monotonic and quickly reaches a good orbital basis. Across all five problems, SHCI's orbital optimization fails to reach TrimCI's level; in particular, `rot_03`–`rot_05` show almost no descent from the initial energy, consistent with the visual comparison in Suppl. Fig. 14. Panel (b) follows each method into the expansion stage that uses the optimized basis. TrimCI's Phase 1+2 reaches E_{var} within 13–21 mHa of E_{FCI} across all five problems at $N_{\mathrm{dets}} = 10^7$. SHCI's expansion, despite reaching $N_{\mathrm{dets}} = 7$–30 million, lands at −325 to −327 Ha with 0.4–1.9 Ha residual error from E_{FCI}.

The cost comparison is informative. Per inner CI, TrimCI uses 100 determinants × 64-run ensemble and completes in roughly 3 min on 48 CPU cores. SHCI uses roughly 600,000 determinants per inner CI and completes in roughly 16 min on the same hardware. TrimCI's inner-CI wavefunction is therefore 6000× more compact, the cycle runs about 5× faster, and the resulting orbital basis is much better and supports an FCI extrapolation.

7.4. Comparison with DMRG-CASSCF

A second natural comparison is DMRG-CASSCF, a standard workflow combining a DMRG inner solver with a CASSCF outer loop. We applied PySCF's 2-step CASSCF (`mc2step`) to the same scrambled `rot_01` FCIDUMP, using Block2 SU(2)-DMRG (bond dimension $M = 500$) as the inner solver, with intra-active orbital rotation explicitly enabled (`internal_rotation = True`), and a Fiedler reorder of the orbitals as a DMRG-standard preprocessing step. We ran twelve macro iterations.

Suppl. Fig. 16 reports the result. The DMRG-CASSCF trajectory does not converge. Over twelve macros the energy oscillates in the band [−325.80, −325.52] Ha without monotonic descent. The solver is, however, actively trying: $\|U - I\|_F$ grows from 0.73 after macro 1 to 1.99 after macro 12, so the outer step is taking substantial rotations in orbital space, but those rotations neither aggregate into a descent direction nor improve over the initial DMRG energy at $U = I$ (−325.83 Ha). The mechanism mirrors the one we saw for SHCI in Suppl. Note 7.3: on a scrambled basis the $M = 500$ DMRG inner solver does not capture the $[Fe_4S_4]$ correlation accurately enough for the orbital gradient computed from its 1- and 2-body reduced density matrices to point in a consistently useful direction. On the final basis we further ran a DMRG sweep up to $M = 4000$ (see Suppl. Fig. 16c); E_{var} has only descended to −326.363 Ha, still ~ 0.9 Ha above the FCI limit, which shows that

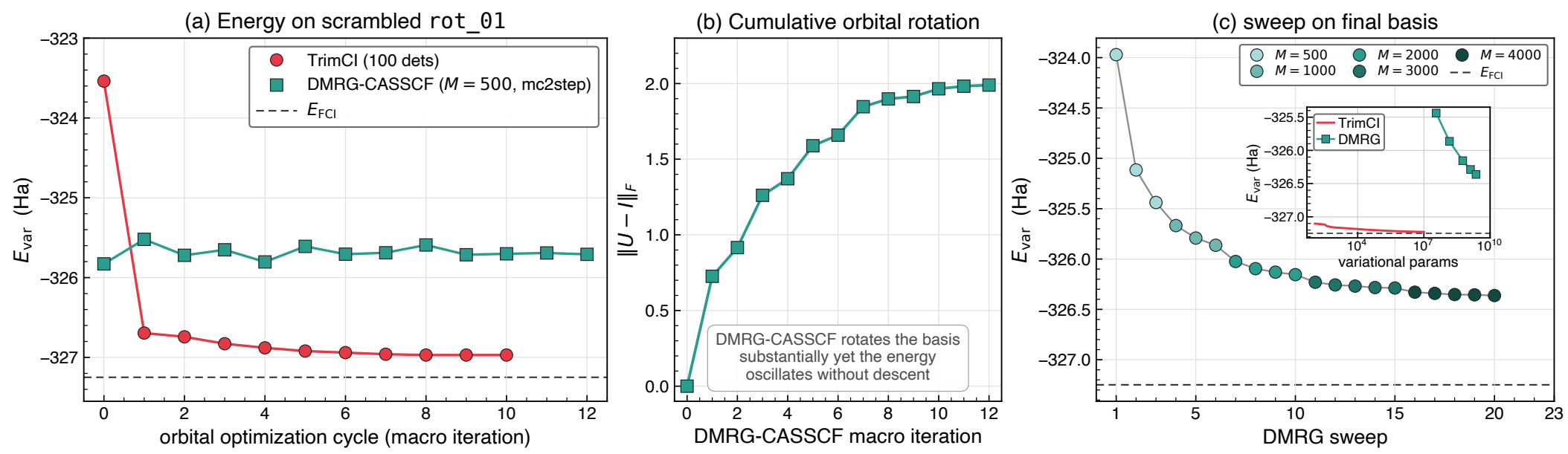

Suppl. Fig. 16. Orbital-optimization trajectories on the scrambled `rot_01` [Fe_4S_4] Hamiltonian. **(a)** Energy vs. iteration. TrimCI (red, 100 determinants per cycle) descends by ~ 3.3 Ha over the first three cycles. DMRG-CASSCF ($M = 500$, green, twelve macro iterations) rotates the orbital basis substantially but the resulting energy oscillates without descent in the band $[-325.80, -325.52]$ Ha, ending ~ 1.5 Ha above the FCI reference. The dashed line marks $E_{\mathrm{FCI}} = -327.249$ Ha [6]. **(b)** $\|U - I\|_F$ for the DMRG-CASSCF trajectory grows from 0.73 at macro 1 to 1.99 at macro 12, so the failure is not stagnation but a misdirected gradient. **(c)** DMRG sweep on the final orbital basis with M grown from 500 to 4000 over 20 sweeps. Even at $M = 4000$, E_{var} remains ~ 0.9 Ha above the FCI limit. Inset: E_{var} vs. variational parameters (one CI coefficient per determinant for TrimCI; $4\,N_{\mathrm{site}}\,M^2$ for DMRG). Source data are provided as a Source Data file.

DMRG-CASSCF cannot rotate the scrambled orbitals back to a basis that supports an efficient variational expansion. TrimCI, by contrast, reaches $E_{\mathrm{var}} = -326.828$ Ha by cycle 3 from a 100-determinant core wavefunction, and the orbital basis it converges to supports a variational expansion that extrapolates to the FCI limit (as established in Section 7.1).

We note a caveat on the interpretation of this comparison. A compact CI wavefunction can in principle be represented by an MPS of modest bond dimension, so the difficulty observed here is not one of expressive power. Moreover, we did not explore alternative MPS initializations for the inner DMRG solver. This comparison should therefore be read as characterizing this particular workflow within the current implementation of Block2, rather than the intrinsic capability of DMRG.

7.5. Why this matters

The above tests and comparisons reveal a property of TrimCI that is independent of the single-particle orbital basis. From a 100-determinant compact TrimCI wavefunction on a randomly rotated basis, the maximally uninformative one available, the 1- and 2-body reduced density matrices already contain enough information to drive the orbital basis toward one that supports an FCI extrapolation.

Two implications follow. First, the TRIM procedure is effective at identifying a very compact wavefunction that captures the ground-state physics even in the worst case of random orbitals. The compact wavefunction is not centered on an HF-like reference; it lives in a region of determinant space that the random rotation hides, and TRIM finds it anyway. Second, for a general problem where a physically reasonable orbital basis is not known a priori, TrimCI offers a promising route to discovering one. The full three-phase workflow and the orbital optimization feature are implemented in the open-source TrimCI release and ready to use out of the box.

Suppl. Note 8. PRACTICAL SCALE AND SCALING PATH

Most TrimCI calculations reported in the main text and in the preceding sections of this SI were carried out at up to 10^8 determinants on a single commodity CPU node (48 cores, 1 TB RAM). This section documents how the TrimCI calculation extends to billion-determinant scale on a GPU node, characterizes the empirical scaling law observed across several GPU models, and outlines the practical scale for large calculations. The intent is to give the reader a clear picture of where TrimCI sits today (reaching state-of-the-art energy at $\sim$1–10 billion determinants on a single GPU node) and the feasible scaling path for pushing the frontier with more resources (10^{10}–10^{12} determinants).

8.1. TrimCI at 2.56×10^9 determinants on a single GPU node

We report here the largest TrimCI calculation on a single GPU node, performed on the Fe_4S_4 BS1 problem CAS(54e, 36o) with $N_{\mathrm{det}} = 2.56 \times 10^9$ variational determinants. This scale matches the largest published UDMRG calculation for the same problem ($E_{\mathrm{var}} = -327.241697\,\mathrm{Ha}$) [6]. TrimCI was performed on a single GPU node (4×NVIDIA H200 with

150 GB HBM each), running the production TrimCI Davidson engine. The Davidson loop reached the energy-convergence criterion $|\Delta E| < 10^{-5}$ Ha at iteration 4 ($\sim$4.5 hours of wall time) and continued to iteration 14, where $|\Delta E| = 1.5 \times 10^{-6}$ Ha and the final variational energy was $E_{\text{var}} = -327.241618$ Ha. Total wall time to iteration 14 was approximately 16.7 hours. The main cost per iteration is the matvec ($\sim$2,645 seconds, i.e. 44 minutes), with the remaining $\sim$40% of the per-iteration wall time spent on non-matvec Davidson overhead (Krylov operations, residual evaluation, checkpoints). Suppl. Table 7 summarizes the convergence trajectory.

Suppl. Table 7. **Davidson trajectory for the $N_{\text{det}} = 2.56 \times 10^9$ reference calculation** (Fe_4S_4 BS1, 4×H200). Wall-time stamps are relative to the start of the Davidson loop.

Iter	E_{var} (Ha)	$\|\Delta E\|$ (Ha)	$\|r\|$	Cumulative wall
1	−327.2412049	–	2.35×10^{-2}	1 h 34 min
2	−327.2415422	3.4×10^{-4}	5.56×10^{-3}	2 h 29 min
3	−327.2415694	2.7×10^{-5}	3.06×10^{-3}	3 h 25 min
**4**	**−327.2415788**	$\mathbf{9.4 \times 10^{-6}}$	2.67×10^{-3}	**4 h 27 min**
5	−327.2415853	6.5×10^{-6}	2.21×10^{-3}	5 h 29 min
8	−327.2415997	4.1×10^{-6}	1.84×10^{-3}	8 h 51 min
10	−327.2416081	4.2×10^{-6}	1.81×10^{-3}	11 h 17 min
12	−327.2416147	2.9×10^{-6}	1.43×10^{-3}	13 h 54 min
**14**	**−327.2416183**	$\mathbf{1.5 \times 10^{-6}}$	1.03×10^{-3}	**16 h 42 min**

Iteration 4 marks the first crossing of $|\Delta E| < 10^{-5}$ Ha; iteration 14 is the deepest converged point.

8.2. Performance on workstation-class GPUs

To study performance on different GPUs, we ran the 2.56-billion-determinant Davidson calculation on a node with 2×NVIDIA L40S (47.7 GB VRAM each).

The matvec on 2×L40S took $\sim$4,400 seconds per iteration, only $\sim$1.7× slower than the $\sim$2,645 seconds on the 4×H200 reference, despite L40S having an order of magnitude lower peak memory bandwidth than H200. The compensating factor is L40S's larger L2 cache (96 MB per GPU vs 50 MB on H200), which holds more of the sparse working set close to

the compute.

8.3. Empirical scaling law

To characterize how the per-iteration matvec cost grows with N_{det}, we collected ten timing measurements at six different scales between 10^6 and 2.56×10^9 determinants, on six GPU models (P100, A100, H100, L40, L40S, H200) and three multi-GPU configurations (1, 2, and 4 GPUs per node). All measurements use the same Fe_4S_4 BS1 CAS(54e, 36o) Hamiltonian and the production TrimCI Davidson engine. To remove the dependence on GPU count, each timing was normalized to a 4-GPU equivalent via $t_{\text{4GPU}} = t_{\text{actual}} \times n_{\text{GPU}}/4$. Suppl. Fig. 17 plots the normalized matvec time versus N_{det} on log–log axes; Suppl. Table 8 lists the raw data.

Suppl. Table 8. **Raw measurements underlying Suppl. Fig. 17.** All timings are wall-clock matvec time per Davidson iteration on Fe_4S_4 BS1 CAS(54e, 36o). The last column is the 4-GPU equivalent used for the fit ($t_{\text{4GPU}} = t_{\text{actual}} \times n_{\text{GPU}}/4$).

N_{det}	GPU model	n_{GPU}	matvec/iter (s)	4-GPU equiv. (s)
1.0×10^6	P100	1	4.48	1.12
1.0×10^6	H100	1	0.27	0.07
1.6×10^7	A100	1	13.4	3.35
3.2×10^8	L40	4	97	97
6.4×10^8	L40S	2	637	318.5
6.4×10^8	H100	2	765	382.5
6.4×10^8	A100	2	1285	642.5
1.28×10^9	L40	4	1980	1980
2.56×10^9	H200	4	2645	2645
2.56×10^9	L40S	2	4400	2200

Despite spanning six GPU models and three different GPU counts per node, all data collapse onto a single power law

$$t_{\text{matvec}} \propto N_{\text{det}}^{\alpha}, \qquad \alpha \approx 1.2. \tag{5}$$

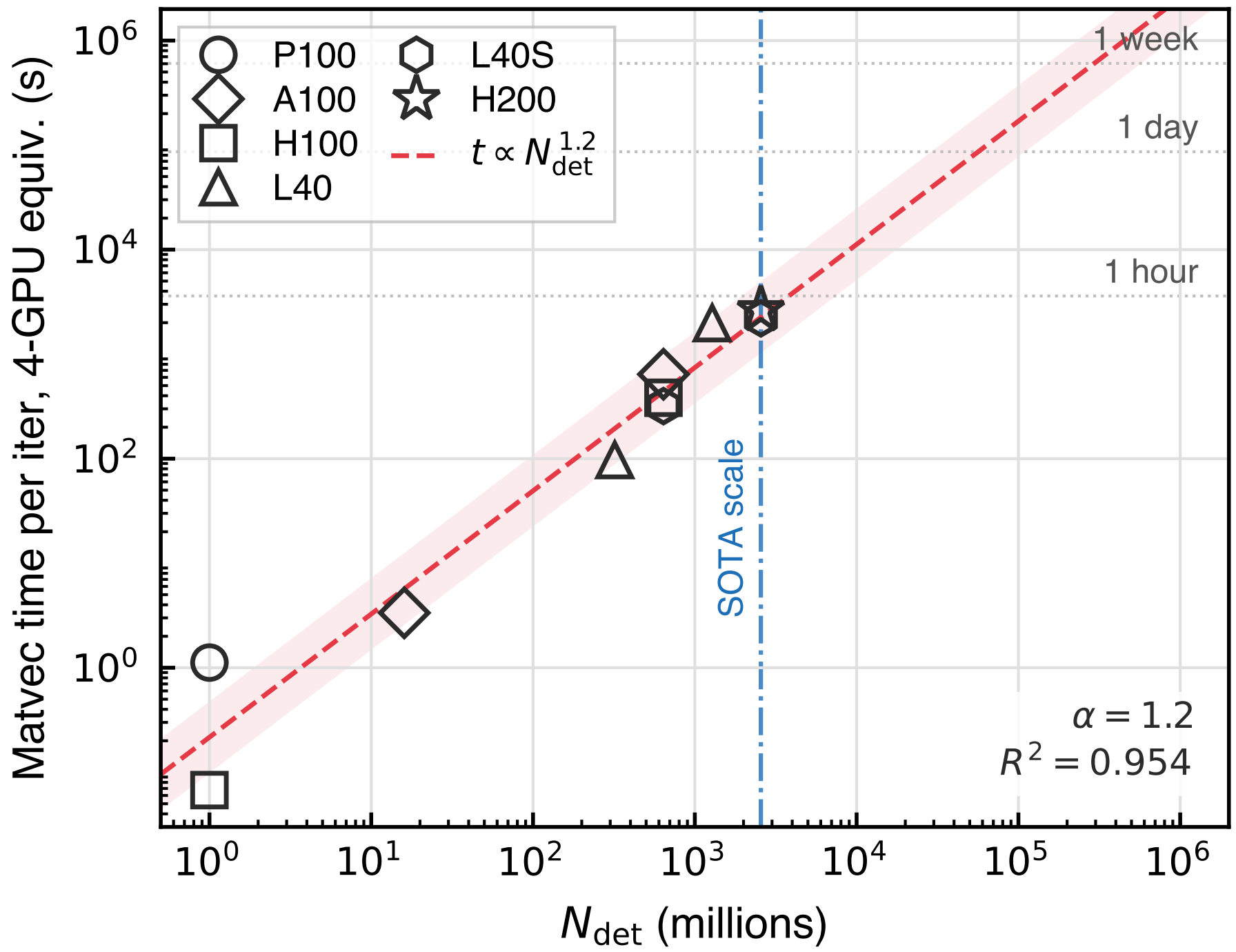

Suppl. Fig. 17. **Empirical scaling of GPU matvec time per Davidson iteration for TrimCI.** Each marker shape encodes a GPU model; all timings are normalized to a 4-GPU equivalent. The single power-law fit across all ten data points gives $t \propto N_{\rm det}^{1.2}$ with $R^2 = 0.954$; the shaded red band shows the $\pm 1\sigma$ envelope in log space (factor $\sim$2.2). The vertical dash-dotted line marks the 2.56-billion-determinant scale, at which TrimCI reaches state-of-the-art accuracy on this Hamiltonian (Suppl. Note 8.1); horizontal dotted lines mark 1 hour, 1 day, and 1 week of wall-clock time. Data sources: see Suppl. Table 8. Source data are provided as a Source Data file.

This near-linear scaling reflects the sparse structure of the Hamiltonian matrix in TrimCI: each determinant has a bounded number of nonzero connections (bounded by the orbital and electron counts), so the matvec is fundamentally $\mathcal{O}(N_{\rm det})$, with the mild super-linearity coming from data-fetch overhead that grows slowly with $N_{\rm det}$. This gentle scaling is what makes very large determinant counts tractable in practice.

8.4. Scaling to trillion determinants

Using Eq. 5 together with the 4-GPU baseline at $N_{\rm det} = 2.56 \times 10^9$ ($t_{\rm matvec} = 2{,}645\,$s) and an 8-iteration Davidson loop (a warm-start Davidson in TrimCI typically converges in 5–10

iterations), we estimate the GPU count required to complete the loop within $\sim$1 day at three target scales. We take the per-iteration wall time to be $\sim 1.4\times$ the matvec time, reflecting the $\sim$40% non-matvec overhead (Krylov operations, residual evaluation, checkpoints) observed in the 4×H200 reference run. In practice, distributing the matvec across multiple GPUs and chunking it to fit VRAM introduce additional overhead; we did not consider these in the estimates below.

10^{10} **determinants on a single 8-H200 node.** The estimated per-matvec wall time on a standard 8-H200 DGX node is $\sim$1.9 hours, and a full Davidson loop would complete in $\sim$21 hours.

10^{11} **determinants on a GPU cluster.** A 1-day Davidson loop requires $\sim$110 GPUs, i.e. $\sim$14 H200 nodes.

10^{12} **determinants on a large cluster.** A 1-day Davidson loop requires $\sim$1,800 GPUs ($\sim$225 H200 nodes), which matches the scale available on large GPU clusters.

These projections are feasible because of the near-linear exponent $\alpha \approx 1.2$. Under quadratic scaling ($\alpha = 2$), reaching 10^{12} from 2.56×10^9 would cost $\sim$160,000× more compute, putting the trillion-determinant regime out of reach. The observed $N^{1.2}$ scaling reduces that factor to $\sim$1,300×, which is within reach in practice. By comparison, DMRG algorithms exhibit a wall-time cost $t \sim N^{1.5}$ in the number of variational parameters N [6]; the smaller-exponent $N^{1.2}$ scaling of TrimCI may therefore offer a favorable wall-time scaling for large-scale calculations.

SUPPLEMENTARY REFERENCES

[1] Z. Li and G. K.-L. Chan, Spin-Projected Matrix Product States: Versatile Tool for Strongly Correlated Systems, Journal of Chemical Theory and Computation **13**, 2681 (2017).

[2] A. A. Holmes, N. M. Tubman, and C. J. Umrigar, Heat-Bath Configuration Interaction: An Efficient Selected Configuration Interaction Algorithm Inspired by Heat-Bath Sampling, Journal of Chemical Theory and Computation **12**, 3674 (2016).

[3] S. Sharma, A. A. Holmes, G. Jeanmairet, A. Alavi, and C. J. Umrigar, Semistochastic Heat-Bath Configuration Interaction Method: Selected Configuration Interaction with Semistochastic Perturbation Theory, Journal of Chemical Theory and Computation **13**, 1595 (2017).

[4] J. Li, M. Otten, A. A. Holmes, S. Sharma, and C. J. Umrigar, Fast semistochastic heat-bath configuration interaction, The Journal of Chemical Physics **149**, 214110 (2018).

[5] H. R. Larsson, H. Zhai, C. J. Umrigar, and G. K.-L. Chan, The Chromium Dimer: Closing a Chapter of Quantum Chemistry, Journal of the American Chemical Society **144**, 15932 (2022).

[6] H. Zhai, C. Li, X. Zhang, Z. Li, S. Lee, and G. K.-L. Chan, Classical computational simulation of the FeMo-cofactor model to chemical accuracy and its implications (2026), arXiv:2601.04621 [physics].

[7] H. Zhang and M. Otten, Absorbing many-body correlations into core-optimized orbitals (2026), arXiv:2605.22977.
