# Supplementary material for: An auto-initializing algorithm to determine electronic ground states from random Slater determinants
Source: Nat Commun. 2026 Sep 29;17:11225. doi: 10.1038/s41467-026-77968-1 (PMC13720135; doi:10.1038/s41467-026-77968-1)
Supplement: Supplementary file 2 [file 41467_2026_77968_MOESM2_ESM.pdf]

nature portfolio

Peer Review File

An auto-initializing algorithm to determine electronic ground states from random Slater determinants

Corresponding Author: Professor Matthew Otten

This file contains all reviewer reports in order by version, followed by all author rebuttals in order by version.

Attachments originally included by the reviewers as part of their assessment can be found at the end of this file.

Version 0:

Reviewer comments:

Reviewer #1

(Remarks to the Author)
The TrimCI method is proposed and benchmarked, detailing an approach to find sparse variational expansions. It is appropriately benchmarked against state of the art approaches in this field, and achieves some impressively compact CI expansions. These compact CI expansions are important in the field of correlated many-electron systems, and how compact the expansion can be (and how to find it) is an important current research question in the field. It is a shame that the paper didn't explore more the topic of excited states, as this is perhaps more of the frontier at the moment, but the ground state questions it asks are still very relevant. Overall, the method is interesting, and certainly adds to the discussions around CI expansions for strongly correlated systems at the forefront of the field. I would be happy to support publication in principle, after a number of further considerations in the manuscript.

My main feeling on reading the paper, is that I would like more understanding of why the method is achieving more compact expansions than other related methods. The TrimCI method itself proceeds along the many selected CI methods, in that it involves sequences of expansion and trimming/pruning steps to ultimately find compact expansions in an iterative fashion. In my view, there are only relatively minor differences in the core methodology from alternatives (e.g. heat-bath SHCI). This is not a criticism, or something that diminished potential impact. However, it begs the question of what is fundamentally driving these more compact expansions. The expansion step seems identical, while the trimming stage is divided into two, first trimming from the CI vector over randomized blocks, and then globally. This local trimming likely allows a larger pool to be initially kept, but ultimately should reduce to SHCI in the limit of all determinants kept in the local trimming stage. Furthermore, if you can afford to do global trimming only (because the pool is small enough), then presumably this is a more effective subsequent trimming strategy, since you include all the coupling between local blocks in determining the importance of different determinants? Therefore, I fail to see how this local trimming is ultimately driving the lower expansion lengths, unless it is a direct consequence of larger pools (and therefore a limitation in the ability to effectively sample in SHCI). This should be able to be tested easily enough by comparison to SHCI in the limits where they should become effectively equivalent. The paper should clarify more clearly the key differences from SHCI, and therefore focus on how this method connects to others like it, and how this leads to the more compact expansions seen (and perhaps a little less 'selling'!).

The other key difference is that the state is initialized from different random configurations. For the reason given above, I suspect that this is more important in providing more compressed expansions in these strongly correlated systems, where the previous systems were being 'biased' by starting in general from a Hartree—Fock reference, even where this configuration is not needed. This should be clarified, with explicit comparisons. If so, it begs the question of how many random initial configurations are required as the system grows larger, in order to find appropriately compact determinant expansions? It is clear that the approach is selecting different determinants to SHCI (when restricted to a variational set), and I can only think that it is this random initialization which is driving this.

This leads to key questions: what is the variance of the final energy and expansion length for different random initializations, and how does this scale with system size? Are the initial random set chosen completely randomly? The question of the number of random initializations is rather neglected, and seemingly key to understand more clearly the novel contributions of this method which build on prior art and improve the compactness of the expansion, and its viability going forwards to larger

systems. How many iterations are required, how many seeds and random initial realizations of the core set are chosen? How robust is this, and are the results 'cherry-picked'? What is the variance of realizations? It is mentioned that the 'Best-performing run is chosen', but if the difficulty in finding this set is just moved to the requirement of an exponential number of random initial guesses, then this is just shifting the exponential complexity of the problem to another part. If these random initializations are important, then it perhaps speaks to a non-ergodicity / slow mixing of the SHCI algorithm in these strongly correlated systems, which validates the importance of the random initialization of selected CI methodology in strongly correlated systems, and the algorithmic innovations in this work. From this viewpoint, this strengthens the manuscript rather than weakens it, as it will then impact upon the way we think about initializing determinant expansions and touch many other methods.

My other main critique of the paper, is that the language and discussion is at times a little too 'salesman' and repetitious, while perhaps not representing fairly the position of TrimCI in the context of many-body methods. There is an extended discussion about the fact that there is a vanishing fraction of determinants for a given relative error in the thermodynamic limit. I think this discussion is a little misleading, and should be made more explicit in what this actually means. We always expect selected CI expansions (which TrimCI is) to be exponentially scaling wrt system size, and if doubt wants to be placed on this, then it would require overwhelming evidence. We know the size of the Hilbert spaces, and therefore, we can convert the ratio expression in Eq. 8 into a scaling of number of determinants with respect to N, which would look like an exponential with an exponent given by the ratio of the empirically found value to the Hilbert space dimension exponent. This would more clearly represent the computational effort scaling with system size, and clarify the scaling of the method as exponential, but with a reduced exponent (for this system) – unless the exponents matched. This would be interesting, and be a clearer predictor of the scope of the method, rather than considering the fraction of Hilbert space spanned.

The other thing that crops up frequently through the manuscript is that the method achieves its accuracy in a fully 'unbiased' fashion, and 'without relying on any prior knowledge'. While this doesn't require a 'trial' state, this statement still isn't quite true in my opinion. TrimCI is 'efficient' when the state can be represented by a sparse expansion of CI amplitudes, and this won't always be the case. It is also still relying on choice of basis for a fundamentally sparse representation (I'm not complaining, this is fine, I just would dispute the idea that this is free from any assumptions or biases – no method other than FCI is, and this just biases towards sparse solutions / low-rank Gaussianity). This assumption that this sparse expansion exists for an accurate state is the modelling assumption of the method, and requires a basis in which this is expected to be true, which are prior assumptions and knowledge which bias the result and inform the success of the approximation. This is not a limitation – all approximate methods have modelling assumptions which underpin their approaches, but this is a required assumption. However, I think the repeated stressing of these aspects of the method that it is completely devoid of prior knowledge is not strictly true, and unnecessary, and should be removed/tempered/caveated. Related to this, this choice of orbital basis should be clarified for all models (Hartree—fock basis?), especially for the Hubbard model, which I presume is taking place in the plane wave basis, but could be the site basis. This is critical to assumptions of a sparse solution.

Specific additional questions:

- The method seems to be lacking dynamic correlation where the purely variational approach is taken, without relying on a perturbative correction. Does the advantage of TrimCI over SHCI reduce in cases where there is substantial dynamic correlation?

- Which results with TrimCI are state-of-the-art, against all other approaches? This will require presumably the extrapolations and perturbative corrections, but would be good to clarify where this is the case. In fig. 3, you are comparing to variational DMRG, but have not extrapolated this result with respect to bond dimension, meaning it is not a particularly fair comparison.

- In figures 4a and 4c, there seem to be areas of different power law convergence, with discontinuous jumps between them. Why is this?

- All the discussions around amplitude distributions, algorithmic efficiency and many-body complexity are interesting, but IMO a little too general. The issue is that it is viewed very much from the perspective of a variational linear wave function amplitude complexity, and does not consider e.g. the impact of orbital rotations on the complexity (which can turn a dense wave function into a sparse one), or other efficiently simulatable wave function parameterizations, such as those based around tensor networks or stabilizer states, which can encode exponential complexity efficiently. This section certainly needs more caveats as to the type of many-body complexity it is encoding, and the more limited scope in terms of ramifications for algorithmic complexity.

- The discussion of integration with quantum algorithms section is a little naive, and perhaps should be removed. Just because you have a sparse wave function in terms of number of non-zero amplitudes, it doesn't mean that this can be efficiently encoded on a quantum device in low depth for initializing quantum algorithms. Encoding an arbitrary wave function in general is not a simple task, and without more details of how this would be achieved, or how TrimCI is particularly suited for this, it perhaps is too much of a digression to be a valuable addition to the discussion. Furthermore, use of TrimCI for initializations of tensor networks is also a little hard to envisage. Ultimately though, I don't think the manuscript needs this – the TrimCI can hold up a paper on its own as a state of the art variational method, rather than have to be something that needs further refinement.

(Remarks on code availability)
Code looks to be in a good state, and confident of reproducibility of results.

Reviewer #2

(Remarks to the Author)
Please see attached PDF file.

(Remarks on code availability)
The code is provided as a clean github repository. It is a python based code. The code accessibility is transparent. Documentation is clear. I did not try to download or run tests cases.

Reviewer #3

(Remarks to the Author)
I co-reviewed this manuscript with one of the reviewers who provided the listed reports. This is part of the Nature Communications initiative to facilitate training in peer review and to provide appropriate recognition for Early Career Researchers who co-review manuscripts.

(Remarks on code availability)

Reviewer #4

(Remarks to the Author)
The article by Zhang and Otten proposes a new kind of selected CI methods where one starts from random determinants and then searches through the configuration space following some procedure. The work makes several claims, principal of which is that this `Trim-CI' outperforms other variants of selected CI. There are other claims, namely that the Trim-CI calculations actually produce good results for specific systems.

Selected CI is not a single method, but a family of methods, and there is great flexibility with how the various aspects: initialization, selection, extrapolation can be done. In this sense, the comparison with selected CI methods then depends rather on how the comparison method is actually performed. Although the authors do not clearly specify how the "comparison" selected CI method is actually done (see below), most of the gains versus `SHCI' seem to be because SHCI used a bad starting point (e.g. in the FeS clusters). [The comparison to the quantum selected CI work for the 4Fe-4S cluster is just comparing to a very bad selection procedure, so it's not really fair and I will not discuss it further]. But for these molecular problems it is not hard to choose a better starting point. Trim-CI might be claimed to be a better heuristic for molecular problems to find good starting determinants, but as a heuristic, should one not then compare to a `good faith' use of other heuristics? The text often says that the method is information free, but this also means the method can be fooled, e.g. if one happens to have sparsely connected determinants and starts in the wrong place.

Put another way, many methods rely on initialization: the comparison tensor network methods discussed also rely on initialization, and in fact, even quantum algorithms for ground-state require good initialization. Finding such better initializations is useful in practice for sure, but I suppose the question is whether that is the kind of intellectual advance that justifies publication.

Another dimension by which to assess the paper is whether the results produced are, in an absolute sense, impressive. Here I note that the authors do not compare to the state-of-the-art. For Cr2, they compare to a minimal basis result, which could have been computed exactly ~15 years ago, rather than to the more modern literature which uses a large basis with selected CI. For [4Fe-4S] they produce an extrapolated number that is rather far below their quoted DMRG reference number, and in poor agreement with the very recent [4Fe-4S] `exact' benchmarks in arXiv:2601.04621. In the Hubbard model, the results beyond exact diagonalization in larger clusters require an extraordinary amount of extrapolation, which seems completely unreliable away from half-filling where there are competing orders and one is unlikely to capture the correct order with a small number of variational states. Certainly, a new method requires time for optimization and for one to learn how to apply it to reach the state of the art. However, I am simply pointing out that the method does not yet produce state of the art results, the main claim is that it is state-of-the-art by comparison to other variants of selected CI, when they use other variants perhaps without full care.

A small but important point is that I found the paper to be lacking in details regarding the comparisons. I could not find supplementary information to explain how the SHCI was done, or indeed exactly how the AFQMC was run, what codes were used and so on and so forth. Since the paper hinges on some details, it is important that this is remedied.
Overall, I think the authors have produced a useful additional to the selected CI literature which may, with detailed and careful application, one day lead to us learning new things about physical systems, although that point has clearly not yet been reached. At the same time, I am not sure the method has the level of profundity attributed to it in the text: it is a neat and `automatized' way to initialize a selected CI procedure, but in my reading that is the main contribution.

(Remarks on code availability)

Version 1:

Reviewer comments:

Reviewer #1

(Remarks to the Author)
The authors have taken on board the previous suggestions seriously, and performed additional studies to evidence quite conclusive responses. It is interesting that the main driver of the advantage seems to be the fact that the random local-then-global sampling strategy of TRIM seems to be the primary mechanism of advantage, rather than the anticipated impact of the random initialization. This emphasises the importance of this broader exploration approach compared to sCI methods generally. I have been convinced in the response by the value that the TrimCI procedure provides over (e.g.) SHCI and related selected CI approaches as the SOTA in strongly correlated molecular systems. Whether this surpasses the sufficiency requirement of the journal is a subjective question, but I believe that it can find broad utility and impact, and would be happy to recommend publication.

As a small point, while the reply says that the best-of-64 runs were taken for all results in the ensemble selection procedure, these important simulation details seem to be omitted from the main text. While I appreciate that the details of the simulation procedures are in the SI, enough of the details which impact upon an understanding of the method should be included in the main text to faithfully judge the results and simulation procedure.

(Remarks on code availability)
Code looks documented and reasonable, but haven't run it myself.

Reviewer #2

(Remarks to the Author)
Please see attached PDF file.

(Remarks on code availability)
Refer to my first round of revision.

Reviewer #3

(Remarks to the Author)
I co-reviewed this manuscript with one of the reviewers who provided the listed reports. This is part of the Nature Communications initiative to facilitate training in peer review and to provide appropriate recognition for Early Career Researchers who co-review manuscripts.

(Remarks on code availability)

Reviewer #4

(Remarks to the Author)
The revised version of the paper and the response letter add some additional calculations that help to strengthen the authors' case that TrimCI is a useful method. In general I agree that the additional calculation on Cr2 (at one geometry, one where the HF reference is a very poor starting point) and [4Fe-4S] (not included in the paper) do show that, in practice, TrimCI performs better than SHCI starting from the HF reference. Thus, there is a path towards claiming that, amongst current implementation of SCI, and assuming one doesn't do much work on the initialization, TrimCI does achieve competitive performance, although the case for the paper is weakened by the fact that the [4Fe-4S] example included in the response is not included in the paper.

At the same time, there is still a sense of overclaiming in the writeup of the paper. TrimCI is optimizing over the initial state, something that can be (and is also sometimes) done in SHCI, as a separate procedure called the initialization, but here is defined as part of SCI algorithm itself. While the response letter states that they are in agreement with this criticism, in practice the changes to the paper are quite minimal (e.g. changing "without relying on any prior knowledge" to "without relying on prior knowledge about good initialization" is not really much of a change). For example, if you initialize SHCI by always doing a CAS calculation and picking those determinants - as has been done before - is that really so different? The practical advance is that the random search turns out, for the systems studied, to be quite a good way to find an initial set of states, and in fact may be better than other ways that have been used in the past.

I think the results achieved are now quite encouraging, and in principle, I think it is now more convincing as a publication. However, I would discourage the authors from retaining this language about lack of prior knowledge compared to prior algorithms, because it really depends on what you define to be "inside" the SCI algorithm as opposed to "outside" of the algorithm. At the very least, the other referee reports show that this emphasis is controversial. Rather the authors should focus on the fact that the heuristic TRIM procedure is good at capturing certain patterns of CI coefficients in determinant space that are not solely centered around the HF determinant.

(Remarks on code availability)

Version 2:

Reviewer comments:

Reviewer #4

(Remarks to the Author)
One of my main concerns about the manuscript was related to the emphasis on “no prior knowledge in the initialization”. I had asked the authors to remove this type of wording, a concern shared by the other reviewers.

The authors have essentially rejected this recommendation, and have included some additional numerics which, from my perspective, don’t support their argument at all (which I discuss below). I am not sure why the authors are sticking to their guns about this. What puzzles me is:

- Do the authors *truly* believe their algorithm is *theoretically* efficient without prior knowledge? This must of course be simply false, because even for a purely classical problem of ground-state optimization, the complexity is in NP. Any algorithm that is known to be efficient *must* then rely on prior knowledge. The fact that multiple reviewers have asked the authors to soften their language surely shows that this is not a trivial concern, and insisting on this type of careless language, in my view, simply makes the paper less credible.
- The authors may argue that they aren’t using any *obvious* problem specific information in their algorithm. I agree with this, but one can’t have it both ways - the algorithm can’t be highly efficient in general (as discussed above). Therefore, for the subset of problems where it is efficient, it must be using structure - in fact the structure, is (by definition) the one compatible with the initialization algorithm they have chosen! So, if one claims efficiency, it is because one knows that the structure is compatible (even if it’s not particularly chemically obvious) - this is the prior knowledge.
- From a practical perspective, I pointed out last time that one can initialize selected configuration interaction methods in many different ways, for example using active space wavefunctions or other techniques. The authors then counter that some of these techniques will not be successful in all problems (for practical reasons). But, the same is true of the authors’ method. Any initialization procedure can be defeated, for example, one may consider the work in https://arxiv.org/pdf/2603.03496 which is designed to defeat a certain type of selected CI initialization.

The authors must believe that removing this type of language weakens their paper. My opinion remains that being careful and rigorous in the writing simply prevents them from being attacked and will make for a better paper. For example, “simulated annealing” is a strategy that can be used in NP hard problems - but one wouldn’t write “simulated annealing is an efficient algorithm for NP hard problems with no prior knowledge”- something that would lead to consternation – one either writes “simulated annealing is an algorithm which does not use prior knowledge in NP hard problems” (i.e. no claim of efficiency) or “simulated annealing is an efficient algorithm for NP hard problems when it is known that competing minima are separated by barriers that are not too wide”.

What I think is defensible to state that the authors have an *automated* initialization procedure, which in a variety of problems, they have shown to work quite well. And this is a positive and useful result.

Some other minor points:

For Fe4S4, the authors are still comparing not to the lowest variational energy, but just one of the reported numbers in the cited paper. The reader will presumably assume that the comparison is to the lowest variational energy, so I think the authors have to say that this is not the lowest variational result. At the same time, when reading the main text, I see no indication of this additional result (it still states “Furthermore, extrapolation of variational and perturbatively corrected TrimCI energies yields an ultimate estimate of–327.263 Ha, consistent with the DMRG benchmark result of–327.240 Ha at bond dimension d= 4000”). I don’t know why the SI result is hidden, but since “benchmark” often means high accuracy, I would at the very least remove benchmark from the main text (and presumably cite the SI here).

The new section on comparing CASSCF optimization with DMRG and selected CI is flawed. The CI wavefunction with 100 determinants can *in the worst case* be represented by a DMRG wavefunction with bond dimension 100, so the fact that the CI wavefunction optimizes better than DMRG with bond dimension 4000 is to do with other things – most likely, the fact that the default initialization procedure in the block2 implementation of DMRG simply assumes that one has a dominant determinant, and the authors did not explore other options. This is just one choice in DMRG that is used in quantum chemistry, but not in other applications of DMRG. It highlights once again that all these methods (DMRG, selected CI) are deployed with a variety of heuristics to initialize, and *require* some heuristic information about the type of problem (in other words, prior knowledge) to be successful.

(Remarks on code availability)

Version 3:

Reviewer comments:

Reviewer #4

(Remarks to the Author)
The authors have addressed my concerns.

(Remarks on code availability)

Open Access This Peer Review File is licensed under a Creative Commons Attribution 4.0 International License, which permits use, sharing, adaptation, distribution and reproduction in any medium or format, as long as you give appropriate credit to the original author(s) and the source, provide a link to the Creative Commons license, and indicate if changes were made.
In cases where reviewers are anonymous, credit should be given to 'Anonymous Referee' and the source.
The images or other third party material in this Peer Review File are included in the article's Creative Commons license, unless indicated otherwise in a credit line to the material. If material is not included in the article's Creative Commons license and your intended use is not permitted by statutory regulation or exceeds the permitted use, you will need to obtain permission directly from the copyright holder.
To view a copy of this license, visit https://creativecommons.org/licenses/by/4.0/

Point-by-Point Response

"From Random Determinants to the Ground State"

Manuscript ID: NCOMMS-25-95448

We thank the reviewers for the thorough and constructive evaluation of our manuscript. The reviews have substantially improved both the rigor and the presentation of our work. Below we provide a point-by-point response, with major revisions organized around four themes. Detailed data and analysis are provided in the Supplementary Information (SI) S1–S6.

1. **Comprehensive fair benchmarking**: We design a *randomly generated strong-correlation benchmark* of 10 unknown systems (CAS(36o, 54e), derived from [4Fe-4S]) to ensure a fair comparison on equal footing. TrimCI achieves significantly lower energy than SHCI—with both closed-shell and open-shell initialization strategies—on all 10 systems, tested up to 10 million determinants, at both variational and PT2-corrected levels (SI S1).

2. **PT2-level comparison**: Epstein–Nesbet PT2 corrections are computed for both TrimCI and SHCI across all benchmark systems, ablation study and the Cr_2 large-basis test. TrimCI+PT2 remains consistently lower than SHCI+PT2 (SI S1, S3, S6).

3. **Physical validation**: We verify that TrimCI converges to the correct ground state through leading determinant analysis and 3d bond density visualization on Cr_2 STO-3G (SI S4), and through AFM order parameter comparison with exact FCI on Hubbard 4×4 (SI S5). Multi-seed variance statistics and ablation studies confirm robustness (SI S2, S3).

4. **State-of-the-art Cr_2 large-basis benchmark**: On Cr_2 cc-pVDZ-DK CAS(28e, 76o)—the same system studied by Larsson et al.[1]—TrimCI reaches the best published SHCI variational energy ($\sim 10^9$ determinants) with $\sim 100\times$ fewer determinants. At 10^8 determinants, TrimCI's E_{var} is 17 mHa lower and E_{var}+PT2 is 3.4 mHa lower than the best SHCI, using $\sim 6\times$ fewer core-hours on a single compute node (SI S6).

Summary of Major Revisions

In the revised manuscript, we have carried out several major new experiments to address the reviewers' concerns regarding benchmarking fairness and the inclusion of dynamical correlation. Below we summarize these revisions before proceeding to the point-by-point responses.

[1] Larsson, H. R., Zhai, H., Umrigar, C. J. & Chan, G. K.-L. The Chromium Dimer: Closing a Chapter of Quantum Chemistry. *J. Am. Chem. Soc.* **144**, 15932–15937 (2022).

Table 1: Summary: how major concerns are addressed.

Concern	SI	Key finding
Not fair comparison; SHCI used poorly	S1, S6	10 blind systems, both HF & open-shell inits: TrimCI wins all 10. Average 39× fewer dets for same E_{var}, 113× for $E_{\text{var+PT2}}$.
No var+PT2 comparison	S1, S3, S6	TrimCI+PT2 consistently below SHCI+PT2 across all systems and all initialization strategies.
Excited-state trapping?	S4, S5	SHCI trapped in HF basin for Cr_2 and Hubbard model; TrimCI converges to the correct ground state with wavefunction analysis and AFM order parameter analysis (2% vs 28% error).
What drives compactness?	S3	Ablation isolates the trim procedure in TrimCI as the dominant factor (0.5–1.1 Ha gain); robust across initializations.
Robustness / seed sensitivity	S2	∼20 runs suffice; every single run outperforms SHCI at matched det count.
Not state-of-the-art results	S6	Cr_2 cc-pVDZ-DK CAS(28e,76o): 100× fewer dets for same E_{var}; 6× fewer core-hours than SHCI.

1. Comprehensive benchmark on 10 strong-correlation systems with PT2 correction included (SI S1). To ensure a fair comparison, we designed a benchmark of 10 unknown systems derived from [4Fe-4S] (CAS(36o, 54e)) via random orbital rotations and noise perturbations. On each system, SHCI (with two generic initializations) and TrimCI are compared up to 10^7 determinants at both variational and PT2-corrected levels. As Fig. 1 shows, TrimCI (orange) achieves lower energy than both SHCI variants (blue, green) on **every single system**. The advantage is visible at all scales—from 10^2 to 10^7 determinants—and persists after PT2 corrections (dashed lines):

To match TrimCI's energy at 10^5 determinants, SHCI requires on average 39× (HF init) or 33× (open-shell init) more determinants at the variational level, and 113× or 51× more at the var+PT2 level (geometric mean over 10 systems; SI S1, Table S2). In the worst cases (orbital-rotated systems), the ratio exceeds 1,000× at the PT2 level. Notably, the advantage does not diminish with PT2 corrections—it grows, because SHCI's PT2 quality is also degraded when it is trapped in a poor variational basin.

We also perform ablation studies (SI S2, S3) showing that even when TrimCI starts from the same initial condition as SHCI (HF determinant), TrimCI still outperforms SHCI by a large

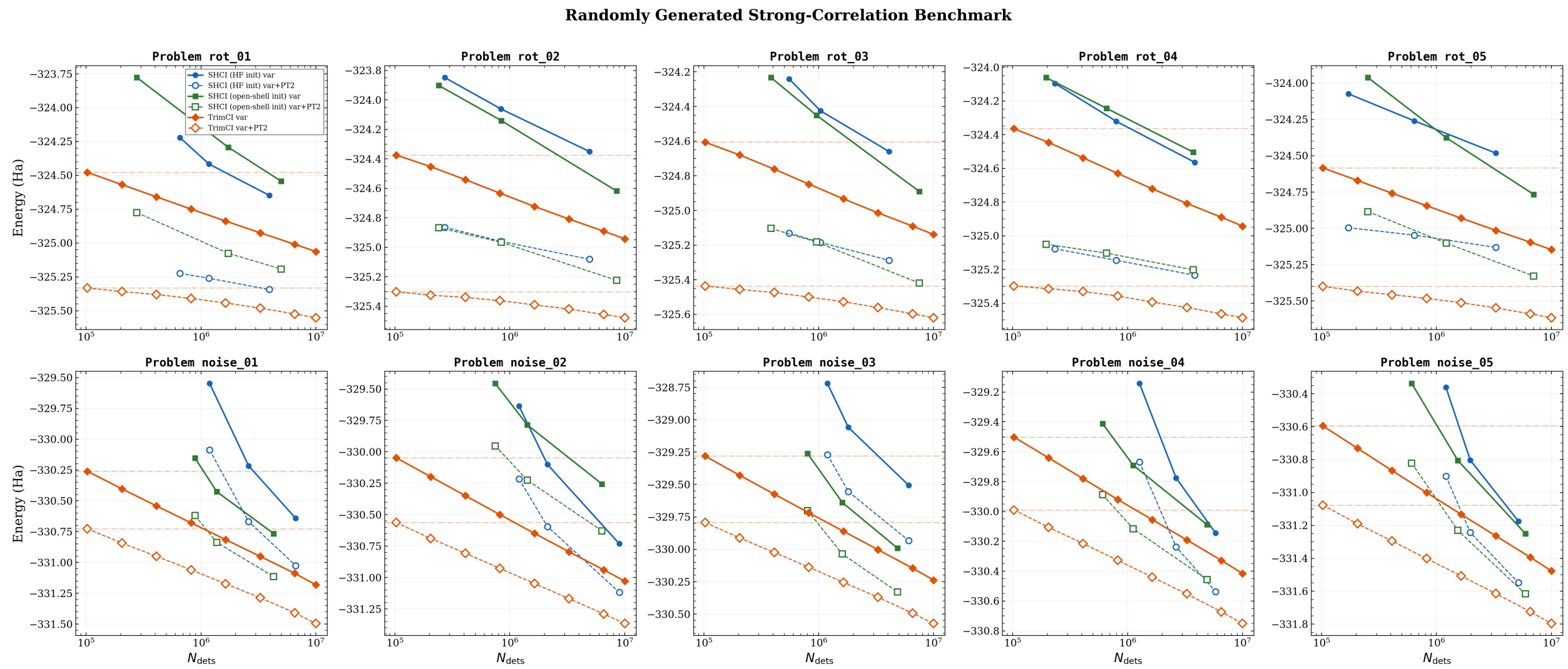

Figure 1: Energy vs. number of determinants for all 10 randomly generated systems. Solid lines: variational energy. Dashed lines with open markers: var+PT2. Blue: SHCI (HF init). Green: SHCI (open-shell init). Orange: TrimCI. Horizontal dash-dot lines mark TrimCI's energy at 10^5 determinants for reference. TrimCI achieves lower energy on every system at both variational and PT2-corrected levels. See SI S1 for more details.

margin—the trimming mechanism, not the initialization, drives the advantage at both the variational and perturbation levels.

2. Cr_2 cc-pVDZ-DK large-basis benchmark (SI S6). To demonstrate that TrimCI is competitive with the best existing methods in the complete workflow on a real, chemically relevant system, we apply it to Cr_2 at $R = 2.50\,\text{Å}$ in the cc-pVDZ-DK basis with CAS(28e, 76o)—the same system used in the landmark study by Larsson et al. (JACS 2022). As shown in Fig. 2, TrimCI reaches the best published SHCI variational energy ($\sim 10^9$ determinants) using **two orders of magnitude fewer determinants** ($\sim 4 \times 10^6$). At 10^8 determinants, TrimCI achieves E_{var} **17 mHa below** the best SHCI, and E_{var}+PT2 **3.4 mHa below** the best SHCI+PT2. TrimCI reaches the best SHCI E_{var}+PT2 at around 6×10^6 determinants. The complete workflow required 40.2 hours on a single 48-core node ($\sim$ 1,930 core-hours), compared to $\sim$ 12,000 core-hours for SHCI—a $\sim 6\times$ reduction.

3. Physical validation (SI S4, S5). Detailed wavefunction analysis on Cr_2 STO-3G and Hubbard 4×4 confirms that TrimCI converges to the correct ground state (leading determinants, orbital occupations, AFM order parameter matching FCI), ruling out the excited-state trapping concern raised by Reviewer 2.

These results establish TrimCI as a state-of-the-art selected CI method: it delivers superior accuracy to SHCI at both variational and PT2-corrected levels, requires significantly fewer deter-

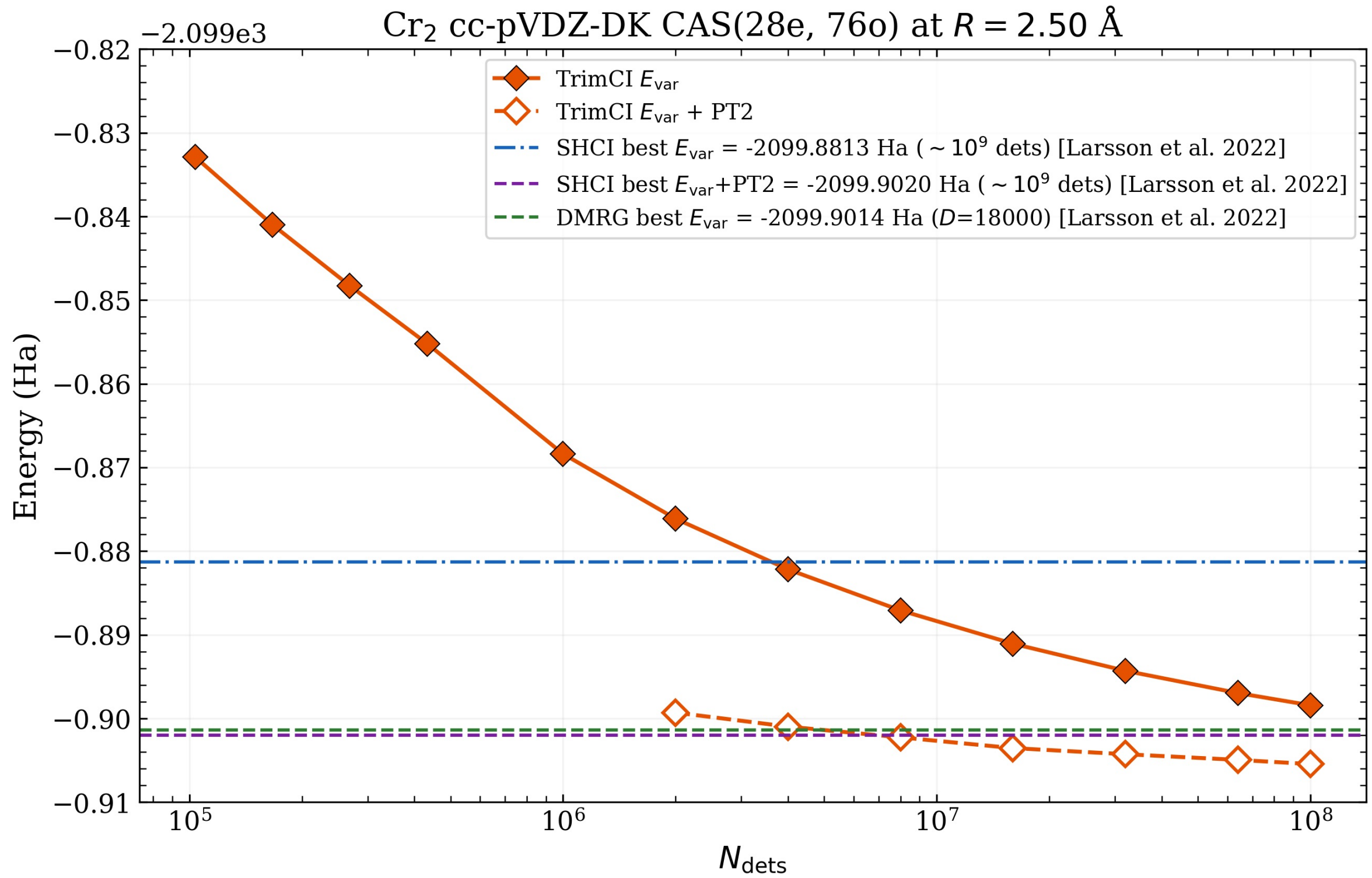

Figure 2: Cr_2 cc-pVDZ-DK CAS(28e, 76o) at $R = 2.50$ Å. TrimCI E_{var} (filled) and E_{var}+PT2 (open) vs. N_{dets}, compared with the best published SHCI and DMRG results from Larsson et al. (JACS 2022). TrimCI reaches the SHCI best E_{var} at $\sim 100\times$ fewer determinants and surpasses both SHCI and DMRG at the PT2 level.

minants and computational resources, and converges to the correct ground-state physics. The fast, efficient implementation also makes it practical for billion-scale determinant calculations.

Response to Reviewer 1

The TrimCI method is proposed and benchmarked, detailing an approach to find sparse variational expansions. It is appropriately benchmarked against state of the art approaches in this field, and achieves some impressively compact CI expansions. These compact CI expansions are important in the field of correlated many-electron systems, and how compact the expansion can be (and how to find it) is an important current research question in the field. It is a shame that the paper didn't explore more the topic of excited states, as this is perhaps more of the frontier at the moment, but the ground state questions it asks are still very relevant. Overall, the method is interesting, and certainly adds to the discussions around CI expansions for strongly correlated systems at the forefront of the field. I would be happy to support publication in principle, after a number of further considerations in the manuscript.

Response: We are deeply grateful to Reviewer 1 for this thoughtful and encouraging assessment, and especially for the support for publication. The Reviewer's incisive questions about what fundamentally drives TrimCI's compactness led to very illuminating new experiments—the ablation study (SI S3) and robustness analysis (SI S2). We also thank the Reviewer for highlighting excited states as an important frontier; we are actively developing excited-state capabilities for TrimCI and plan to release them to the community in the near future. We have addressed all considerations raised, as detailed below.

> *My main feeling on reading the paper, is that I would like more understanding of why the method is achieving more compact expansions than other related methods. The TrimCI method itself proceeds along the many selected CI methods, in that it involves sequences of expansion and trimming/pruning steps to ultimately find compact expansions in an iterative fashion. In my view, there are only relatively minor differences in the core methodology from alternatives (e.g. heat-bath SHCI). This is not a criticism, or something that diminished potential impact. However, it begs the question of what is fundamentally driving these more compact expansions. The expansion step seems identical, while the trimming stage is divided into two, first trimming from the CI vector over randomized blocks, and then globally. This local trimming likely allows a larger pool to be initially kept, but ultimately should reduce to SHCI in the limit of all determinants kept in the local trimming stage. Furthermore, if you can afford to do global trimming only (because the pool is small enough), then presumably this is a more effective subsequent trimming strategy, since you include all the coupling between local blocks in determining the importance of different determinants? Therefore, I fail to see how this local trimming is ultimately driving the lower expansion lengths, unless it is a direct consequence of larger pools (and therefore a limitation in the ability to effectively sample in SHCI). This should be able to be tested easily enough by comparison to SHCI in the limits where they should become effectively equivalent. The paper should clarify more clearly the key differences from SHCI, and therefore focus on how this method connects to others like it, and how this leads to the more compact expansions seen (and perhaps a little less 'selling'!).*
> *The other key difference is that the state is initialized from different random configurations. For the reason given above, I suspect that this is more important in providing more compressed expansions in these strongly correlated systems, where the previous systems were being 'biased' by starting in general from a Hartree—Fock reference, even where this configuration is not needed. This should be clarified, with explicit comparisons. If so, it begs the question of how many random initial configurations are required as the system grows larger, in order to find appropriately compact determinant expansions? It is clear that the approach is selecting different determinants to SHCI (when restricted to a variational set), and I can only think that it is this random initialization which is driving this.*

Response: These are exactly the right questions, and we designed a dedicated ablation study to answer them (SI S3; see Fig. 3). The study uses an unknown problem from S1 (randomized [4Fe-4S], CAS(36o, 54e)) and compares TrimCI with TRIM (the trim procedure in the TrimCI algorithm) enabled against TrimCI with TRIM disabled (pure heat-bath expansion, equivalent to the SHCI expansion strategy), across five initialization conditions (see figure below; here "HF" denotes the Hartree–Fock determinant and "R" denotes a random determinant with the correct

number of up-spin and down-spin electrons).

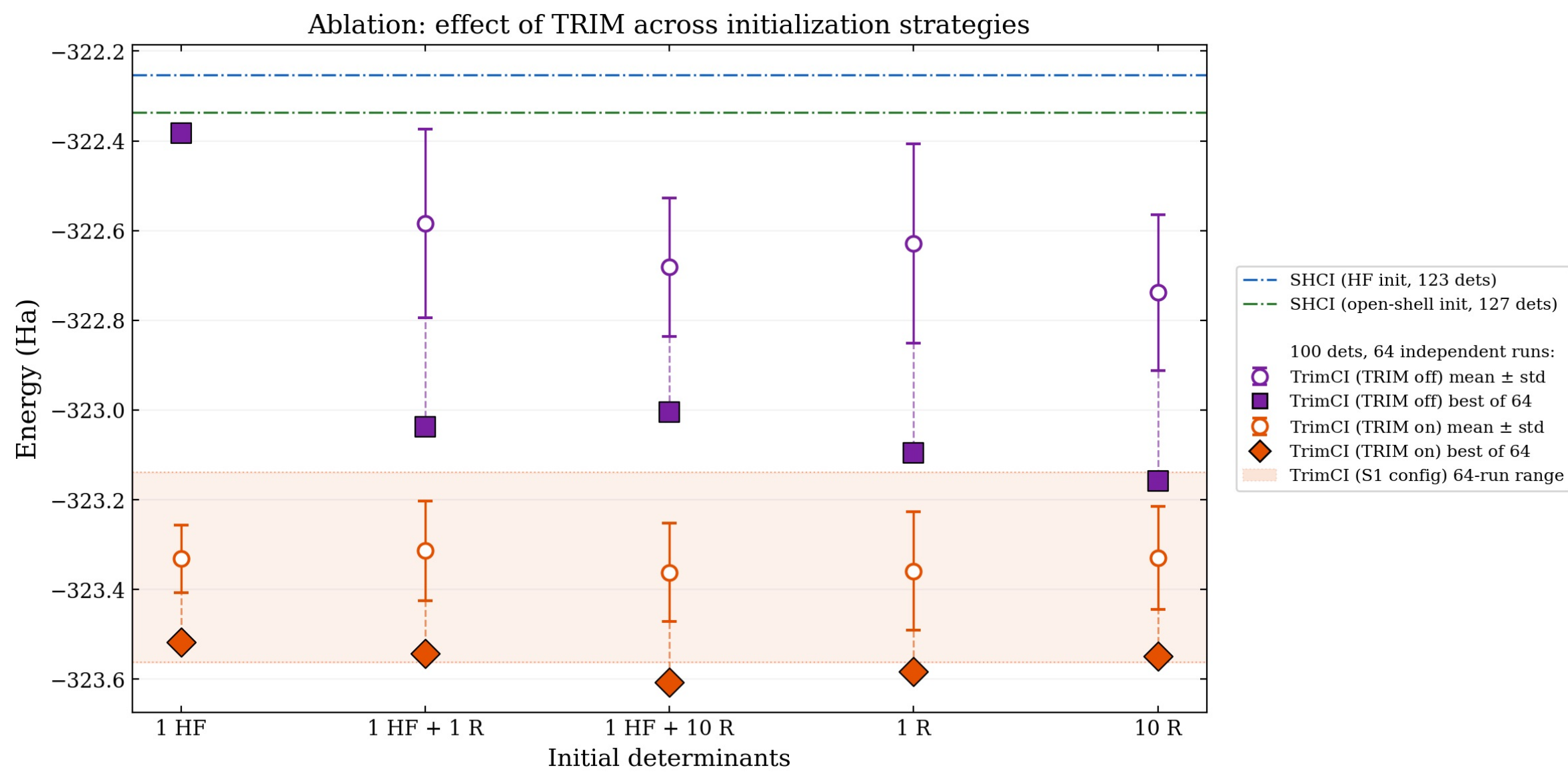

Figure 3: Effect of TRIM at 100 determinants across five initializations (including purely random, no HF). Filled markers: best of 64 runs. Open circles with error bars: mean ± std. Shaded band: 64-run range with the S1 default configuration. Dashed lines: SHCI at comparable size. See SI S3 for details.

The results are clear. **TRIM is the dominant mechanism**, improving the energy by 0.4–1.1 Ha at 100 determinants regardless of initialization:

Init	TRIM on	TRIM off	Δ
1 HF	−323.518	−322.382	−1.14 Ha
1 HF + 1 R	−323.543	−323.037	−0.51 Ha
1 HF + 10 R	−323.607	−323.005	−0.60 Ha
1 R (no HF)	−323.584	−323.096	−0.49 Ha
10 R (no HF)	−323.549	−323.157	−0.39 Ha

A revealing finding is that TRIM-off expansion from a single HF determinant is *fully deterministic*: 64 independent runs produce identical results ($\sigma = 0$). This is precisely the SHCI behavior—the heat-bath expansion follows a single, fixed path through Hilbert space with no mechanism to explore alternatives. TRIM breaks this determinism through its randomized local trim procedure, allowing the method to discover lower-energy determinant subspaces that the monotonic expansion path misses.

Equally importantly, the purely random initializations (1 R and 10 R, no HF determinant) achieve TRIM-on energies of −323.58 and −323.55 Ha—essentially indistinguishable from the HF-seeded results. This confirms that TrimCI is genuinely prior-knowledge-free: with or without the

Hartree–Fock determinant, the iterative TRIM procedure discovers the same low-energy subspace.

We also verify that this advantage is not an artifact of the small (100-det) space. Expanding the TRIM-off baseline to 10^7 determinants, TrimCI (TRIM on) maintains an advantage of 0.44 Ha in variational energy and 0.29 Ha after PT2 correction (SI S3).

The Reviewer's intuition about random initialization is partly correct: adding random determinants does help, particularly when TRIM is off (purple squares in Fig. 3). However, random initialization alone—without TRIM—cannot match the TRIM-on results. The mechanism is synergistic: random initialization provides diverse starting points, and TRIM selects the most important determinants from a large pool through its local-then-global strategy. The local trim also plays a key role in allowing more exploration—a pure global trimming on a small core set may prematurely eliminate determinants that would prove important at larger scales.

This leads to key questions: what is the variance of the final energy and expansion length for different random initializations, and how does this scale with system size? Are the initial random set chosen completely randomly? The question of the number of random initializations is rather neglected, and seemingly key to understand more clearly the novel contributions of this method which build on prior art and improve the compactness of the expansion, and its viability going forwards to larger systems. How many iterations are required, how many seeds and random initial realizations of the core set are chosen? How robust is this, and are the results 'cherry-picked'? What is the variance of realizations? It is mentioned that the 'Best-performing run is chosen', but if the difficulty in finding this set is just moved to the requirement of an exponential number of random initial guesses, then this is just shifting the exponential complexity of the problem to another part.

Response: These are excellent questions. Motivated by these concerns, we designed a dedicated experiment to characterize the variance and seed requirements systematically (SI S2; see Fig. 4), which we summarize here.

We ran 64 independent TrimCI calculations on an unknown problem from S1 (randomized [4Fe-4S], CAS(36o, 54e)), each starting from a completely random set of Slater determinants (uniformly sampled subject to particle number and S_z constraints—no system-specific information whatsoever) and run TrimCI to 100 determinants. All 64 runs used identical hyperparameters (same as in SI S1).

The results are reassuring. The 64 runs yield a mean energy of -323.394 ± 0.089 Ha, with best $= -323.562$ Ha and worst $= -323.138$ Ha—a spread of 0.42 Ha. The variance is real but moderate: the standard deviation (0.089 Ha) is small compared to the advantage over SHCI (see below).

To directly address the concern about cherry-picking: we also ran SHCI on the same system with two generic initializations (HF and open-shell), obtaining -322.253 Ha (123 dets) and -322.336 Ha (127 dets) respectively. Every single TrimCI run—including the worst—outperforms both SHCI results by at least 0.8 Ha. The advantage is therefore not an artifact of selecting the best from many poor runs; it is a systematic feature of the method.

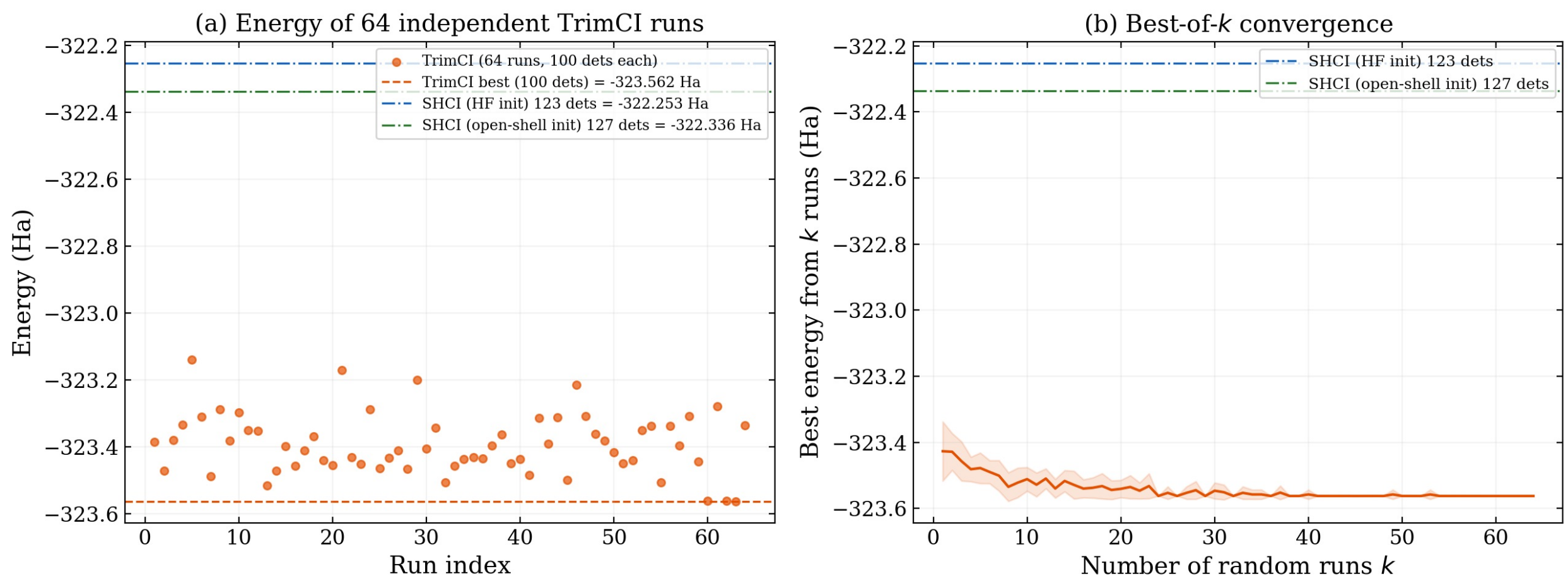

Figure 4: Robustness of TrimCI across 64 independent runs (100 dets each). (a) Energy of each run. Dashed lines show SHCI at comparable size (∼123–127 dets). Every TrimCI run outperforms both SHCI initializations. (b) Best-of-k convergence: ∼20 runs suffice to reach near-optimal energy. See SI S2 for details.

Regarding the number of seeds required: Figure (b) above shows the best-of-k energy as a function of the number of randomly chosen runs. The curve plateaus around $k \approx 20$, meaning that 20 independent runs already capture most of the benefit of the ensemble. We use 64 runs throughout as a conservative choice, but as Fig. 4(b) shows, the best-of-k energy converges rapidly and ∼20 runs already suffice. The difficulty is not shifted to requiring an exponential number of random initial guesses.

If these random initializations are important, then it perhaps speaks to a non-ergodicity / slow mixing of the SHCI algorithm in these strongly correlated systems, which validates the importance of the random initialization of selected CI methodology in strongly correlated systems, and the algorithmic innovations in this work. From this viewpoint, this strengthens the manuscript rather than weakens it, as it will then impact upon the way we think about initializing determinant expansions and touch many other methods.

Response: This is a very insightful perspective on non-ergodicity or slow mixing and determinant expansion, and we fully agree—overcoming non-ergodicity (or slow mixing) is key to making many-body methods efficient in strongly correlated regimes. TrimCI addresses this non-ergodicity through three layers of stochasticity, each serving a distinct role:

1. **Initial determinants.** The initial determinants are randomly generated (satisfying particle number and S_z constraints), so the search begins without bias toward any particular basin. This helps but is not sufficient on its own—the ablation study (SI S3) shows that runs with TRIM off and random init still underperform runs with TRIM on.

2. **Local trimming.** In each TRIM cycle, the pool is randomly partitioned into blocks and each block is diagonalized independently. This introduces stochastic selection *within* each cycle, allowing the method to explore determinant subspaces that a purely deterministic expansion would miss.

3. **Ensemble selection.** We typically run an ensemble of 64 independent searches up to a small core size (10^2–10^4 dets), then select the best-performing run for subsequent large-scale expansion (10^6–10^9 dets). This is inexpensive because the ensemble operates at small N_{dets}, while the costly expansion phase runs only once.

Together, these three mechanisms enable TrimCI to self-correct toward the physically relevant basin even from arbitrary starting points. We believe this algorithmic design for overcoming non-ergodicity in strongly correlated systems may offer insights for other many-body methods facing similar challenges, and may also help deepen our understanding of the physical nature of strong correlation itself.

My other main critique of the paper, is that the language and discussion is at times a little too 'salesman' and repetitious, while perhaps not representing fairly the position of TrimCI in the context of many-body methods. There is an extended discussion about the fact that there is a vanishing fraction of determinants for a given relative error in the thermodynamic limit. I think this discussion is a little misleading, and should be made more explicit in what this actually means. We always expect selected CI expansions (which TrimCI is) to be exponentially scaling wrt system size, and if doubt wants to be placed on this, then it would require overwhelming evidence. We know the size of the Hilbert spaces, and therefore, we can convert the ratio expression in Eq. 8 into a scaling of number of determinants with respect to N, which would look like an exponential with an exponent given by the ratio of the empirically found value to the Hilbert space dimension exponent. This would more clearly represent the computational effort scaling with system size, and clarify the scaling of the method as exponential, but with a reduced exponent (for this system)—unless the exponents matched.

Response: We agree with the Reviewer's critique. We initially found the thermodynamically vanishing fraction to be an interesting observation, but the Reviewer is right that expressing the scaling as a fraction of Hilbert space can be misleading about the actual computational cost. Following the Reviewer's suggestion, we now add a new equation (Eq. 9) that explicitly shows how N_{dets} scales with system size N: $\log_{10} N_{\text{dets}} \approx 0.140\,N$ to leading order. This makes clear that TrimCI scales exponentially, but with an exponent (0.140) that is 23% of the Hilbert space exponent (0.602)—a $\sim 4\times$ compression of the scaling exponent.

The other thing that crops up frequently through the manuscript is that the method achieves its accuracy in a fully 'unbiased' fashion, and 'without relying on any prior knowledge'. While this doesn't require a 'trial' state, this statement still isn't quite true in my opinion. TrimCI is 'efficient' when the state

can be represented by a sparse expansion of CI amplitudes, and this won't always be the case. It is also still relying on choice of basis for a fundamentally sparse representation (I'm not complaining, this is fine, I just would dispute the idea that this is free from any assumptions or biases – no method other than FCI is, and this just biases towards sparse solutions / low-rank Gaussianity). This assumption that this sparse expansion exists for an accurate state is the modelling assumption of the method, and requires a basis in which this is expected to be true, which are prior assumptions and knowledge which bias the result and inform the success of the approximation. This is not a limitation – all approximate methods have modelling assumptions which underpin their approaches, but this is a required assumption. However, I think the repeated stressing of these aspects of the method that it is completely devoid of prior knowledge is not strictly true, and unnecessary, and should be removed/tempered/caveated. Related to this, this choice of orbital basis should be clarified for all models (Hartree—fock basis?), especially for the Hubbard model, which I presume is taking place in the plane wave basis, but could be the site basis. This is critical to assumptions of a sparse solution.

Response: We thank the Reviewer for this detailed and thoughtful explanation. We fully agree: TrimCI relies on the assumption that the ground state admits a sparse expansion in the chosen determinant basis, and the choice of orbital basis is important. By "prior-knowledge-free" we mean that, unlike most many-body methods, TrimCI does not rely on a good starting wavefunction—which is critical in cases where such knowledge is not available. We have revised several statements about prior knowledge in the manuscript to make them more moderate and precise.

Orbital bases are now specified explicitly for all systems: Cr_2 and the Hubbard model use Hartree–Fock canonical orbitals; FeS clusters ([2Fe-2S], [4Fe-4S], P-cluster) use localized orbitals from the literature.

The method seems to be lacking dynamic correlation where the purely variational approach is taken, without relying on a perturbative correction. Does the advantage of TrimCI over SHCI reduce in cases where there is substantial dynamic correlation?

Response: We provide complete PT2 comparisons in SI S1, S3 and S6. In terms of absolute energy differences, yes, the gap narrows at the var+PT2 level compared to the variational level, as expected. However, TrimCI+PT2 remains consistently lower than SHCI+PT2 across all benchmark systems.

Importantly, the advantage measured in *determinant count* does not shrink: SHCI requires on average 33–39× more determinants to match TrimCI's E_{var}, and 51–113× more to match $E_{\text{var+PT2}}$ (Table 2 in SI). This is because near the FCI limit, even small energy differences demand a disproportionately larger number of determinants—so TrimCI's more compact variational space translates into a persistent efficiency advantage at the PT2 level as well.

Which results with TrimCI are state-of-the-art, against all other approaches? This will require presumably the extrapolations and perturbative corrections, but would be good to clarify where this is the case.

Response: The state-of-the-art results are: (1) Cr_2 cc-pVDZ-DK CAS(28e, 76o), where TrimCI+PT2 surpasses the best published SHCI+PT2 by 3.4 mHa, reported in SI S6; (2) [4Fe-4S], where TrimCI at 5.12×10^9 determinants surpasses the state-of-the-art UDMRG($D = 12{,}000$)—see also our response to Reviewer 4; (3) Hubbard model in the main text, where TrimCI+PT2 extrapolation achieves state-of-the-art accuracy comparable to AFQMC; (4) for the molecular benchmarks in the main text, at a fixed number of variational determinants, TrimCI achieves lower energies than other selected CI methods. These energies can be pushed further toward the exact limit by expanding to larger determinant counts if needed.

In fig. 3, you are comparing to variational DMRG, but have not extrapolated this result with respect to bond dimension, meaning it is not a particularly fair comparison.

Response: The variational DMRG energy in Fig. 3 is included as a reference point for the energy scale, not as a direct performance comparison between TrimCI and DMRG.

In figures 4a and 4c, there seem to be areas of different power law convergence, with discontinuous jumps between them. Why is this?

Response: These fluctuations likely arise from fine-grained structure in the Hamiltonian graph—as the variational space grows, newly added determinants may reflect some local structures before the energy resumes smooth convergence. We consider this a secondary effect that does not affect the overall convergence trend.

All the discussions around amplitude distributions, algorithmic efficiency and many-body complexity are interesting, but IMO a little too general. The issue is that it is viewed very much from the perspective of a variational linear wave function amplitude complexity, and does not consider e.g. the impact of orbital rotations on the complexity (which can turn a dense wave function into a sparse one), or other efficiently simulatable wave function parameterizations, such as those based around tensor networks or stabilizer states, which can encode exponential complexity efficiently. This section certainly needs more caveats as to the type of many-body complexity it is encoding, and the more limited scope in terms of ramifications for algorithmic complexity.

Response: We thank the Reviewer for this insightful observation. These caveats are necessary and we have added a dedicated paragraph to the complexity discussion in the revised manuscript

addressing both points. Specifically, we clarify that while the intrinsic complexity σ_0 is defined in the optimal orbital basis, the observed complexity σ_a depends on the basis actually used—a poor basis can significantly inflate σ_a. We also note that alternative parameterizations such as tensor networks and neural quantum states may provide more compact descriptions in terms of number of parameters, and that the relationship between determinantal complexity and the compactness of such parameterized states is an interesting open question.

The discussion of integration with quantum algorithms section is a little naive, and perhaps should be removed. Just because you have a sparse wave function in terms of number of non-zero amplitudes, it doesn't mean that this can be efficiently encoded on a quantum device in low depth for initializing quantum algorithms. Encoding an arbitrary wave function in general is not a simple task, and without more details of how this would be achieved, or how TrimCI is particularly suited for this, it perhaps is too much of a digression to be a valuable addition to the discussion. Furthermore, use of TrimCI for initializations of tensor networks is also a little hard to envisage. Ultimately though, I don't think the manuscript needs this – the TrimCI can hold up a paper on its own as a state of the art variational method, rather than have to be something that needs further refinement.

Response: We sincerely appreciate the Reviewer's recognition that TrimCI stands on its own as a state-of-the-art method, and thank the Reviewer for this valuable and constructive feedback. We fully agree: the quantum algorithms section was a digression that the manuscript does not need. We have removed it from the revised version. In the Discussion, we now emphasize that TrimCI is a complete, efficient workflow that produces compact wavefunctions and state-of-the-art energies without relying on other methods. We retain a brief mention that the compact core may serve as input to other frameworks.

Response to Reviewers 2 & 3

In the manuscript, the authors introduce a selected-CI algorithm, TrimCI, which aims to efficiently identify an important fraction of the Hilbert space through an iterative search and refinement procedure. The method combines heat-bath screening (Eq. 5) with a trimming step applied during the variational stage. The algorithm is clearly described, and its scope is well stated.
According to the authors, the major contributions of the proposed method are that it (1) is prior-knowledge-free, (2) can identify a better variational space than the semistochastic heat-bath CI (SHCI) method, and (3) provides highly accurate results for strongly correlated systems. Numerical results are presented in support of these claims. However, there are several major concerns that seriously undermine the claimed contributions of the proposed method.

Response: The Reviewers provide an accurate and thorough summary of TrimCI's claims and methodology. We thank Reviewers 2&3 for the careful reading and the detailed concerns, which have

significantly strengthened the manuscript. The Reviewers' questions about physical significance, PT2 comparisons, and prior-knowledge-free claims are precisely the right ones to ask, and motivated very substantial new experiments in this revision. We address each major concern below.

1. Physical significance of the results is not sufficiently demonstrated. *For all chemical systems presented,* Cr_2*, Fe4S4 and P-cluster, the authors report that the selected space by TrimCI is much different from that of SHCI, and concentrated near "the physically relevant region" of the Hilbert space. What is the physical relevant region? The authors should discuss more about the one-electron basis used. Did they use HF split-localized (or other forms of localization) or HF canonical orbitals (delocalized)?*

If they used HF canonical orbitals, clearly the closed-shell HF determinant, $|2222222000000000\ldots\rangle$*, will certainly be one of the leading determinants of the ground-state wave function (even though its weight is small). In this scenario, I would actually expect that the SHCI, while having higher energy, is capturing the correct physics (given that the star is part of the selected space), while TrimCI is missing it (given that all dets are far from the star in their figures).*

If they used localized orbitals, then any closed shell determinant, including HF determinant, would have NO contribution to the ground-state wave function. Instead, the $|aaaaaaa, bbbbbbbb, 00000\ldots\rangle$ *might have larger contribution (a and b = alpha and beta electrons) albeit still generally small. The prior knowledge in this aspect is necessary! Initializing SHCI from* $|aaaaaaa, bbbbbbb, 00000\ldots\rangle$ *would be a good choice. I suspect that the authors either have initialized SHCI in a physically poor way, and derailed the SHCI search, or they put little emphasis on the physics/chemistry of the problem, while focusing too much on the numerical aspects.*

If they want to show the strength of TrimCI, they should not rely exclusively on the poor initializationof SHCI. Instead they should guide the SHCI search in a physically meaningful way, and then comparethe results against their TrimCI.

Energy in this context is really not a good measure of quality. It is not to be excluded the following scenario: The ground state wave function is highly multi-determinantal, while the excited state is more compact. A truncated CI would converge faster for the excited state than for the ground state. Both TrimCI and SHCI are far from converged, as demonstrated by the steep lines in Fig. 2 (a, d, g) as a function of the selected space. However, TrimCI might have got stuck to the hypothetical excited state, and the more compact representation is a reflection of the compactness of the excited state. In this scenario the excited state would be artificially stabilized as compared to the ground state, as TrimCI is capturing more correlation for the excited state (whose wave function is more compact) than the SHCI does for the ground state.

To avoid any misinterpretation of the data, I advise the authors to analyze the leading determinantsin both SHCI and TrimCI, and discuss their physical significance. Even better, if authors have accessto observables (expectation values, easily accessible via RDMs), such as spin-spin correlation functions,they should compare those values from both methods. Only by analyzing the physical content of thewave function (leading determinants, observables) one canrule out such a possibility. This becomes moreimportant for the larger clusters (say the Fe4S4 and P-cluster), where the low energy spectrum is denseand as demonstrated by Ref. 62, it is easy to get trapped in an excited state.

And if the SHCI is poorly initialized, the authors should redo the SHCI calculations with a better initial

space, and compare again with TrimCI. Only then it would be clear the superiority of TrimCI over SHCI,not just in the identification of the trial space. The authors could still highlight the advantage of TrimCI being prior-knowledge-free, but at least the energy and convergence between the two methods wouldbe compared on a fair ground.

Response: The Reviewer raises two critically important questions: (1) the role of the orbital basis and SHCI initialization, and (2) whether TrimCI might be trapped in an excited state rather than the true ground state. We address both below with new experiments directly following the Reviewer's suggestions.

1. **Orbital basis and SHCI initialization.** We summarize the orbital basis, initialization, and fairness of comparison for each system:

System	Orbitals	SHCI init	Result
Cr_2 STO-3G & cc-pVDZ-DK	HF canonical	HF det (fair)	TrimCI outperforms SHCI in both basis sets; SHCI trapped in HF basin in (36o, 48e)
Hubbard model	HF canonical	HF det (fair)	TrimCI far outperforms SHCI; SHCI trapped near HF
FeS clusters	Localized (from literature)	HF det & open-shell	Multiple broken-symmetry states exist; finding a good init requires expert knowledge that TrimCI does not need
Randomized Fe_4S_4 (S1)	Unknown (scrambled)	HF & open-shell (fair)	No method can exploit prior knowledge; TrimCI wins on all 10 systems

We elaborate on each case:

i) Cr_2 (HF orbitals, HF init—fair comparison). SHCI starts from the HF determinant in HF canonical orbitals. This is the fair initialization for this system. In the STO-3G basis (36o, 48e), SHCI remains trapped in the HF basin for the first 68,454 determinants before a sudden 0.29 Ha energy drop and escape the HF basin (SI S4 and see below); TrimCI finds the multi-reference region immediately. At 3×10^7 dets, both converge to similar energy (TrimCI lower by 1.0 mHa). In the cc-pVDZ-DK basis (28e, 76o), TrimCI surpasses the best published SHCI by 17 mHa in E_{var} and 3.4 mHa in $E_{\text{var+PT2}}$ (SI S6).

ii) Hubbard model (HF orbitals, HF init—fair comparison). SHCI in HF orbitals with HF initialization is also a fair comparison for this system. On the 4×4 lattice ($U/t = 2$), TrimCI far outperforms SHCI at all determinant counts (see SI S5 for detailed results). SHCI's expansion from HF remains trapped near the mean-field solution. Not only does SHCI yield higher variational energies, but it also fails to capture the correct ground-state

physics: the antiferromagnetic (AFM) spin structure factor $S_{zz}(\pi, \pi)$ computed from the SHCI wavefunction shows a flat 28% error relative to exact FCI, whereas TrimCI's AFM error decreases steadily to 2.3% at 1.8×10^5 dets. This demonstrates that TrimCI converges to the correct ground-state physics, not just a lower energy.

iii) FeS clusters (localized orbitals). These systems have multiple broken-symmetry (BS) states with complex antiferromagnetic coupling patterns. Finding a good SHCI initialization requires expert knowledge of the spin structure—one must know which orbitals are localized on which iron center and align the spins accordingly. This is precisely the motivation of this work: such accurate prior knowledge is unavailable to most users and requires detailed understanding of each orbital's character. TrimCI, starting from random determinants, discovers all relevant BS configurations without this expertise.

Following the Reviewer's suggestion, we test SHCI with several different open-shell initializations on [4Fe-4S]. As shown in Fig. 5, a randomly scrambled open-shell determinant performs even *worse* than HF closed-shell. As the Reviewer suggests, we also test an "up-down" initialization where α electrons fill the orbitals from left to right, then β electrons fill the remaining orbitals, and since there are more electrons than orbitals, the excess β electrons are placed randomly. This improves somewhat, but still falls far behind TrimCI at all determinant counts. In summary, without expert knowledge of the precise spin pattern, all SHCI initializations fall far behind TrimCI.

iv) Randomized Fe_4S_4 (S1 benchmark—fair comparison). The orbital structure is destroyed by random rotations or noise, so no method can exploit prior knowledge. SHCI is tested with both closed-shell (HF) and open-shell initializations. TrimCI wins on all 10 systems, requiring 33–39× fewer determinants at the E_{var} level and 51–113× at the $E_{\text{var+PT2}}$ level (geometric mean; Table S2). Furthermore, in the ablation study (SI S3), we also test TrimCI starting from only the HF determinant; thanks to the trimming mechanism, TrimCI still outperforms SHCI by a large margin, demonstrating that the advantage is insensitive to the initial state.

2. **Leading determinant and energy analysis on Cr_2 (SI S4; Fig. 6).** Following the Reviewer's suggestion, we perform a detailed wavefunction analysis of Cr_2 STO-3G (48e, 36o) at $R = 2.5$ Å. SHCI initialized from HF remains trapped in the "HF basin" for the first 68,454 determinants, where the leading determinants are single/double excitations. At $\varepsilon_1 = 9 \times 10^{-4}$ (293,364 dets), SHCI undergoes a sudden basin escape and the energy drops by 0.29 Ha, with the wavefunction reorganizing into quadruple/sextuple excitations characteristic of multi-reference correlation. TrimCI (100 random determinants, no HF) finds this multi-reference region immediately—its leading determinants match the structure SHCI discovers only after 3×10^5 dets. At $\sim 10^8$ dets, both methods converge to similar energy (TrimCI lower by 1.0 mHa). Notably, even after SHCI escapes the HF basin, TrimCI continues to converge faster toward the ground state, as visible in the inset of the figure below—suggesting that

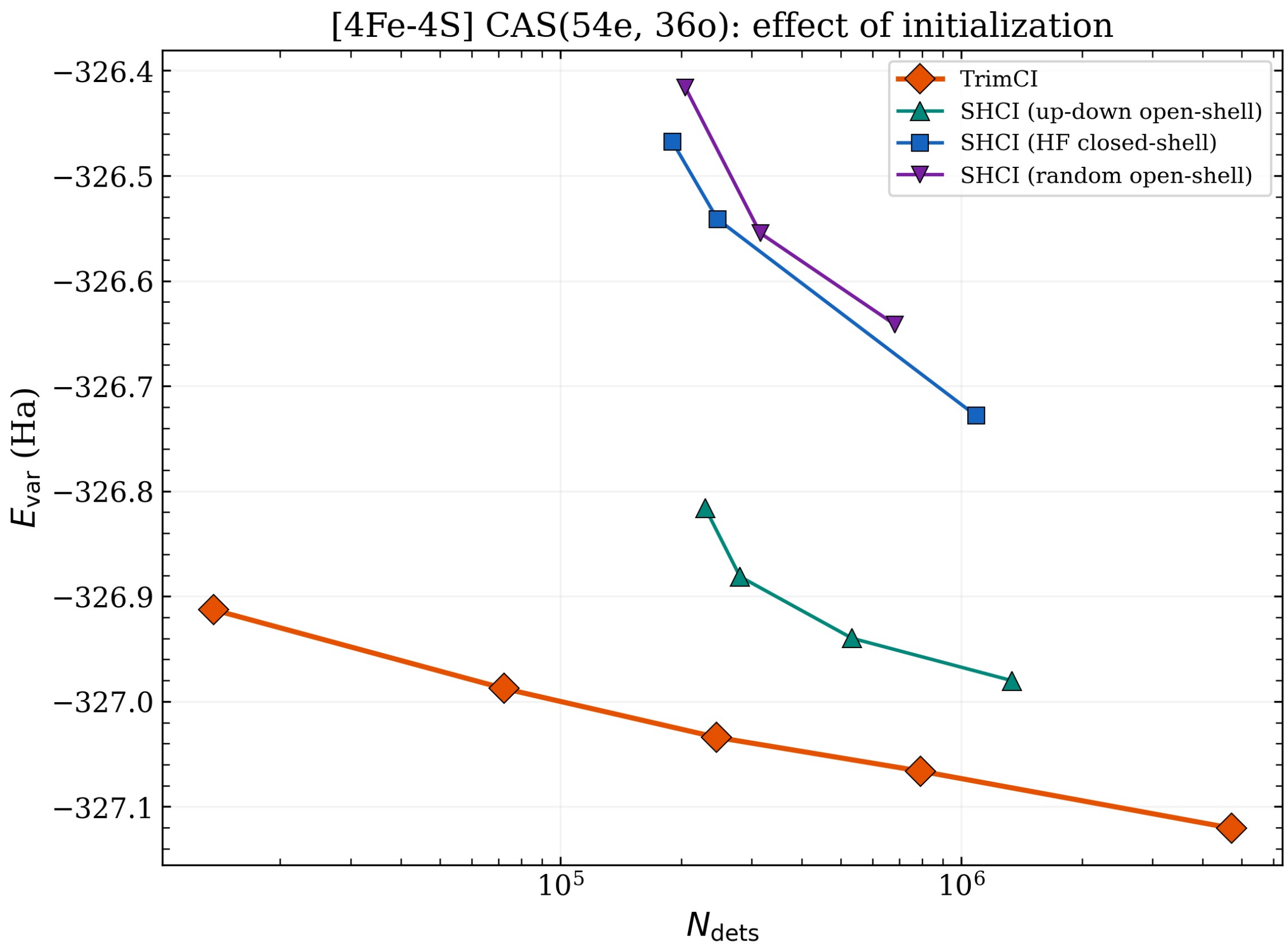

Figure 5: [4Fe-4S] CAS(54e, 36o): TrimCI outperforms SHCI with all three generic initializations at every determinant count.

SHCI might still be partially trapped in a near-ground-state excited configuration.

3. **Physical observable validation on Hubbard (SI S5; Fig. 7).** Following the Reviewer's suggestion, we compute the AFM spin structure factor $S_{zz}(\pi, \pi)$ from the 2-RDM on the Hubbard 4×4 model ($U/t = 2$, 16e, 16o), where exact FCI is available. SHCI's AFM error remains at $\sim 28\%$ across all N_{dets}—it never captures the correct antiferromagnetic correlations. TrimCI's AFM error decreases to 2.3% at 1.8×10^5 dets, confirming convergence to the correct ground-state physics, not just a lower energy. This directly addresses the excited-state concern: an excited state would exhibit qualitatively different spin correlations.

In summary, the comparison between TrimCI and SHCI is fair, particularly with the extensive new experiments in this revision. The most direct evidence is the comparison with the best published SHCI results in the literature (SI S6), where TrimCI surpasses SHCI at both the variational and PT2 levels. Leading determinant analysis, energy convergence behavior, and AFM order parameter measurements all rule out the hypothesis that TrimCI is trapped in an excited state. On the contrary, the evidence suggests that it is SHCI that can become trapped in excited-state basins

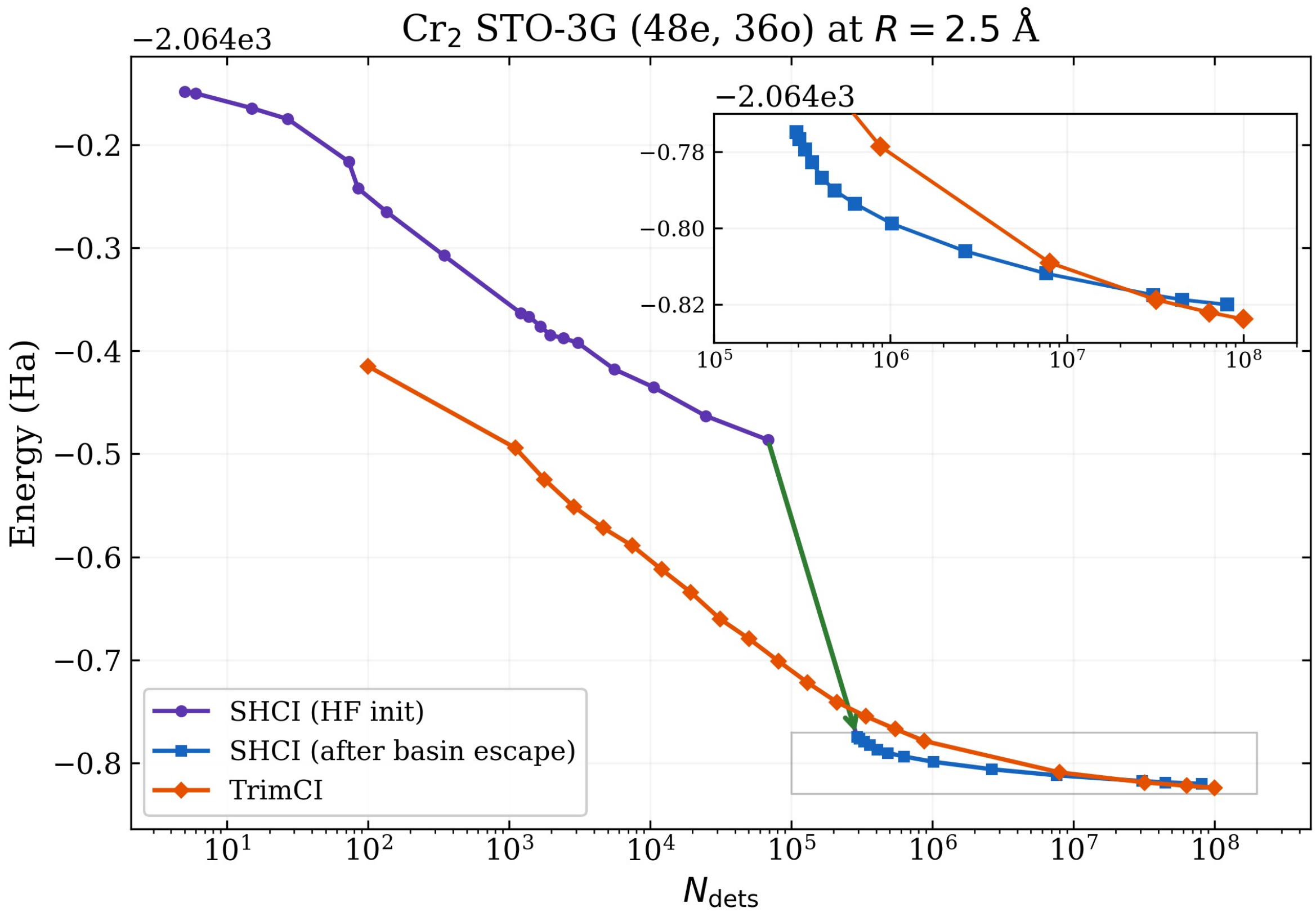

Figure 6: Cr_2 STO-3G (48e, 36o): SHCI trapped in HF basin until $\varepsilon_1 = 9 \times 10^{-4}$ (green arrow, 0.29 Ha drop). TrimCI converges smoothly from the multi-reference region. See SI S4.

when initialized from HF.

2. Only the variational stage of SHCI and TrimCI is compared. *The comparison presented in the manuscript is restricted to the variational stage of the two methods. However, a key strength of SHCI lies in its semistochastic perturbative stage, which largely alleviates the memory bottleneck and is essential to its overall performance. While I understand that the distinguishing feature of TrimCI is the trimming step applied during the variational stage—and that, in principle, this strategy could be combined with the perturbative stage of SHCI—the way the comparison is currently presented gives the impression that TrimCI outperforms SHCI as a complete method.*

Such a conclusion cannot be drawn without explicitly comparing the fully extrapolated PT2 energies obtained from the original SHCI protocol and the TrimCI with PT2 extrapolation. Without this comparison, it is difficult to assess whether TrimCI provides a genuine advantage over existing methods.

In addition, it would be valuable to compare TrimCI with other selected CI methods specifically designed for highly multireference systems, such as TPSCI (https://dx.doi.org/10.1021/acs.jctc.0c00141). In the referenced work, the authors compare TPSCI and HCI across all stages of the calculation and demonstrate that TPSCI achieves lower variational energies with smaller variational spaces, particularly for highly multireference systems. Notably, the PT2 energy differences between HCI and TPSCI are much smaller

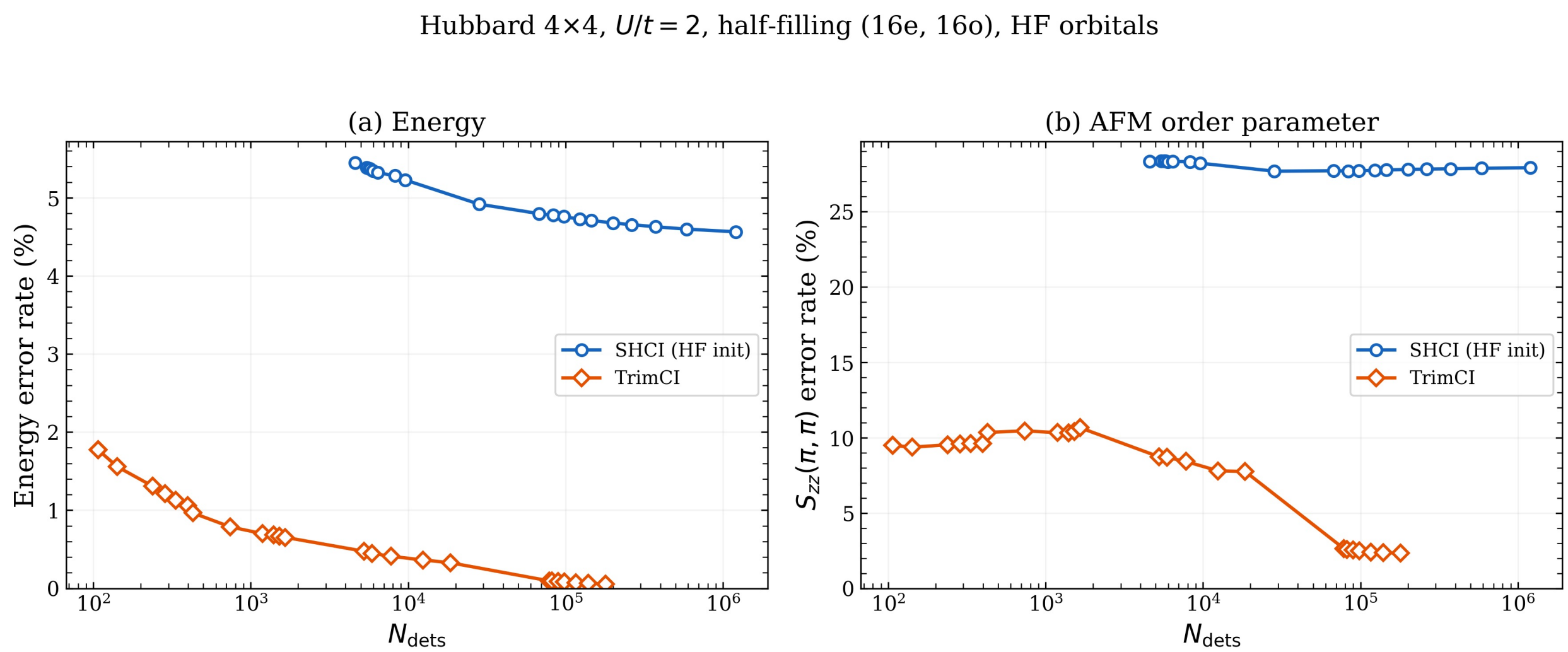

Figure 7: Hubbard 4×4: relative error vs. FCI in energy (a) and AFM order parameter (b). SHCI's AFM error stays at $\sim 28\%$; TrimCI converges to 2%. See SI S5.

> *than the corresponding differences at the variational level, highlighting the importance of comparing complete workflows rather than only the variational stage.*

Response: The Reviewer makes an important and well-informed point: the perturbative correction is essential to the practical performance of selected CI methods, and comparing only variational energies is not enough. We fully agree, and we thank the Reviewer for emphasizing this—the revised manuscript now includes detailed var+PT2 comparisons throughout.

We have added complete var+PT2 comparisons in two key settings:

1. **Randomly generated benchmark (SI S1).** All 10 systems now include PT2 corrections for both TrimCI and SHCI. At the variational level, SHCI requires 33–39× more determinants to match TrimCI's E_{var} at 10^5 dets. At the var+PT2 level, the ratio increases to 51–113× (geometric mean; Table S2), because when SHCI is trapped in a poor variational basin, the PT2 correction is also degraded. The gap narrows in absolute energy compared to the variational level—consistent with the Reviewer's expectation—but the efficiency advantage in determinant count persists or even increases.

2. **Cr_2 cc-pVDZ-DK CAS(28e, 76o) (SI S6).** We directly benchmark TrimCI against the best published SHCI ($\sim 10^9$ determinants) results from Larsson et al. (JACS 2022). At the $\sim 6\times10^6$ determinant scale, TrimCI already matches the best SHCI var+PT2 energy. At 10^8 determinants, TrimCI achieves $E_{\text{var}} = -2099.898$ Ha (17 mHa below SHCI best) and E_{var}+PT2 $= -2099.905$ Ha (3.4 mHa below SHCI best). The complete workflow required $\sim 1{,}930$ core-hours vs. $\sim 12{,}000$ for SHCI. This demonstrates that TrimCI provides a genuine advantage as a *complete method*, including at the PT2-corrected level.

Regarding the comparison with TPSCI (Abraham & Mayhall, JCTC 2020): we note that TPSCI and TrimCI operate in complementary representation spaces. TPSCI achieves compact wavefunctions by working in a *tensor product state* (TPS) basis, where orbital clustering absorbs intra-cluster correlation into the basis vectors themselves. In contrast, TrimCI and SHCI both work in the Slater determinant basis and seek the most compact determinantal representation of the ground state. A wavefunction that is compact in the TPS basis is generally *not* compact in the determinant basis. The two approaches are therefore complementary rather than competing: TrimCI's trimming strategy could, in principle, be applied within a TPS basis to combine the advantages of both representations.

3. The computational parameters used in the calculations are not sufficiently specified or tested. *In Fig. 2, the authors compare the variational energies of TrimCI and HCI as a function of variational space size, showing that TrimCI can reach similar variational energies with substantially smaller variational spaces. However, this apparent advantage is meaningful only if the computational cost and memory requirements required to reach the final variational space are not substantially higher than those of HCI. For example, in an extreme scenario where the parameter "pool_core_ratio" is chosen so large that the TrimCI calculation already requires memory comparable to a full CI calculation, the reduction in variational space size would not constitute a practical advantage. Moreover, since TrimCI introduces overhead due to multiple subspace diagonalizations, claims of computational efficiency based solely on Fig. 2 are not convincing without providing the relevant trimming and expansion parameters. I also assume that the HCI calculations are initiated from the Hartree–Fock (HF) determinant, while the TrimCI calculations employ the ensemble strategy. This information should be explicitly stated in the manuscript. If this assumption is correct, the ensemble strategy should also be applied to the HCI calculations to ensure a fair comparison. In my view, the choice of the initial determinant set is a user-defined input rather than a method-specific feature. For strongly correlated systems in which the HF determinant lacks clear physical meaning, initializing the calculation from a single HF determinant is not a reasonable option.*

Response: Complete TrimCI workflow parameters are provided in SI, including all hyperparameters (Table 1 in SI, held fixed across all 10 benchmark systems), SHCI configuration, and hardware specifications. In particular, for the Cr_2 cc-pVDZ-DK benchmark (SI S6), we provide full details of hardware, runtime. The complete TrimCI workflow required 40.2 hours on a single 48-core node ($\sim$ 1,930 core-hours), compared to $\sim$ 12,000 core-hours for SHCI—a $\sim 6\times$ reduction.

To address the concern about computational cost: the TrimCI trim-and-expand phase (Phase 0) operates on a small variational space (typically 10^2–10^4 determinants), so it is computationally inexpensive. For Fe_4S_4 CAS(54e, 36o), the entire Phase 0 completes in 10–20 minutes on a laptop; the subsequent expansion to millions of determinants dominates the total cost. The early-stage investment in trimming represents a small fraction of the total cost but yields a substantially better variational space.

Regarding the ensemble strategy: in the ablation study (SI S3), we find that the ensemble can

improve SHCI somewhat, but the improvement is modest and SHCI still falls far behind TrimCI—because without the trim mechanism, SHCI's monotonic expansion cannot escape poor basins. TrimCI's dependence on the ensemble is also moderate: even a single random starting point or the HF determinant yields competitive results (SI S3, "1 HF" initialization), because the trim mechanism itself is the dominant driver of compactness.

4. The prior-knowledge-free feature is not sufficiently demonstrated. *A central selling point of TrimCI emphasized throughout the manuscript is its prior-knowledge-free property. Two aspects contribute to this feature: (i) the ensemble strategy, in which several simulations are initiated from different random variational spaces and the best-performing one is selected after a few iterations, and (ii) the local trimming step, which removes determinants based on local information within each variational subspace. However, this claim is not sufficiently supported by the presented results.*
Regarding the first aspect, the sensitivity of the method to the initial composition of the variational space needs to be systematically investigated. If the performance is strongly dependent on the starting space, this could limit the applicability of the method to large systems, where identifying a favorable random initial space becomes increasingly unlikely.
As the ensemble strategy appears more like a user choice than an intrinsic feature of the method, additional evidence is required to demonstrate that the prior-knowledge-free behavior is primarily attributable to the trimming step itself. Although Fig. 5 partially illustrates this point, using 10,000 random determinants for the 4×4 real-space Hubbard model—where Néel states are clearly defined and easily reachable—does not seem to constitute a rigorous test. While Figs. 5(a) and 5(b) are interesting in that the correct time-reversal symmetry is eventually recovered once the variational space reaches approximately 1.7e5 determinants, the number of iterations and the expansion ratios required to achieve this convergence should be explicitly reported.

Response: The ablation study (SI S3) directly addresses both concerns. For convenience, we reproduce the key result here:

As shown, TrimCI is largely insensitive to the initial composition of the variational space: using a single random starting point ("1R") or the HF determinant ("1 HF") yields results comparable to other compositions, e.g. "10R". Thus, the trimming mechanism is the primary driver of performance. Furthermore, TrimCI initialized from the HF determinant successfully escapes the HF basin and converges to the multi-reference region—demonstrating that the method does not require a favorable random initial space. In SI S3, we also compare TrimCI with TRIM enabled vs. disabled (pure heat-bath expansion): disabling TRIM degrades the energy by 0.4–1.1 Ha at 100 determinants, confirming that the trim procedure is the essential ingredient.

5. PT2 extrapolation results are not clearly presented. *The PT2-extrapolated energies are benchmarked against FCI and AFQMC results, primarily in Table 2. However, the table does not clearly indicate the range of variational space sizes used for the extrapolation. In Fig. 4(b), the energies of*

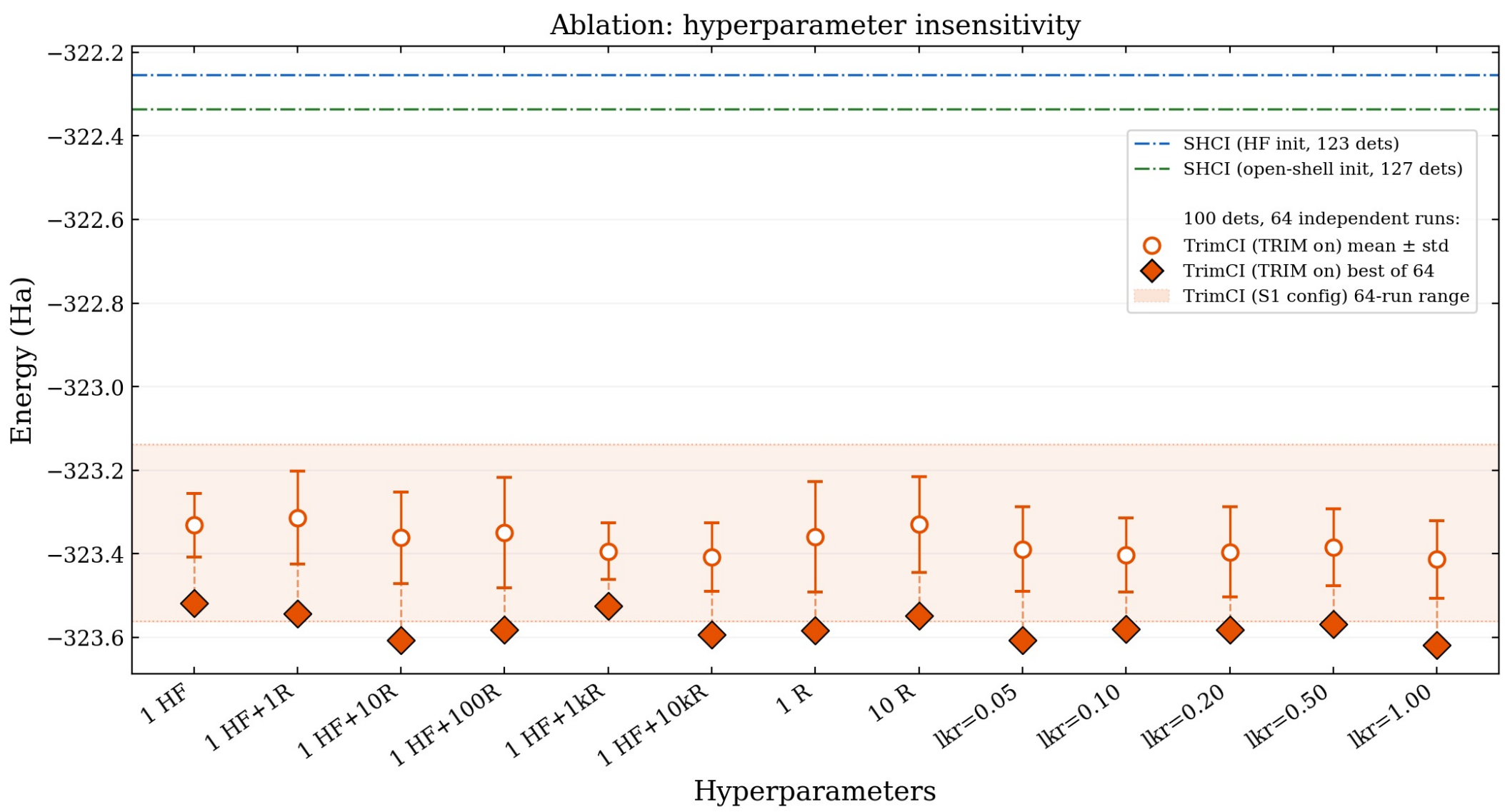

Figure 8: Ablation study (SI S3): sensitivity to initialization. TrimCI is insensitive to the number of initial random determinants (1 vs. 10 random starts) and to the choice of starting point (random vs. HF). SHCI does not benefit from ensemble initialization.

the data points used for extrapolation are all lower than −18.014, whereas the corresponding energies reported in Table 2 are −18 or higher. This presentation is potentially misleading, as it may give readers the impression that highly accurate extrapolated energies can be obtained from very low-cost calculations. In addition, some variational space sizes reported in Table 2 are estimated rather than explicitly given. Since the determinants in the variational space are presumably stored in memory, it is unclear why the exact number of determinants cannot be reported. Clarification is needed regarding how these estimates are obtained and how accurate they are.

Response: We thank the Reviewer for this important reminder. In the revised Table 2, we now include a column $n_{\max}$ that explicitly shows the largest variational space used for each system's extrapolation (ranging from 10^7 to 3×10^8 determinants). Regarding the estimated N_{dets} values: for some accuracy levels (marked "est." in the table), the target accuracy was not directly reached in our calculations, so N_{dets} is estimated from a power-law fit of the variational convergence curve.

6. *In the intro the authors cite ref 62 as a method that requires prior-knowledge. In what aspect that work used prior-knowledge? I might understand that some selected-CI rely on the initial trial wave function, a reference determinant. However, in DMRG work, I would exclude such situations. The authors might want to clarify that point.*

Response: We respectfully note that DMRG can in fact require careful initialization for strongly correlated systems. Ref. 62 describes this challenge explicitly:

> "*the non-linear optimization of the DMRG wavefunction is easily trapped in local minima. To address this, we have designed a new theoretical procedure that combines the simple enumerability of BS-DFT solutions with the representational power of MPS. This involves seeding DMRG calculations from a large number of spin-projected BS-DFT Slater determinants that sample the relevant electronic basins...*"

This illustrates that DMRG can get stuck in local minima and critically depends on a good starting point in strongly correlated systems—precisely the regime where TrimCI's ability to identify the physically relevant region of Hilbert space becomes most valuable. TrimCI's compact variational wavefunction could, in principle, serve as an effective initial state for subsequent DMRG calculation.

> ***7.*** *Very recently the following work has come out: https://doi.org/10.1021/acs.jctc.5c01264. The authors might want to follow that line of research and relate their findings to that. Clearly, a compressed wave function would also favor the TrimCI algorithm (once implemented into a spin-adapted basis).*

Response: This is a good suggestion. We have cited Song & Li Manni (JCTC 2025) in the revised manuscript and plan to explore their Quantum Anamorphosis strategy within TrimCI with spin-adapted basis in future work.

> ***Minor comments:***
>
> 1. *Typo: Right above Eq.5 the authors write "pool set P. by including". We suppose the period after P is a typo.*
> 2. *Authors use the short notation dets for determinants in several places. I would avoid such abbreviations in a formal article and use the extended form or the well accepted SD acronym.*
> 3. *The authors state that the initial space "satisfy the required particle number and spin constraints." They cannot enforce spin symmetry as they are using SDs. They can only enforce the correct S_z value (spin projection). Please correct this statement.*
> 4. *Symbols appearing in Eq. (4) are not defined. For completeness, these should be explicitly introduced.*
> 5. *The selection criterion ($|H_{ij}c_j| > \theta$) does not include an explicit sum over j and allows inexpensive expansion, as noted by the authors. Since this criterion is a key ingredient of the heat-bath CI algorithm (https://doi.org/10.1021/acs.jctc.6b00407), I recommend explicitly referencing this work when describing the expansion step.*
> 6. *The bond length of the chromium dimer is missing from the caption of Fig. 2, although it is mentioned in the main text.*
> 7. *It is unclear which variational spaces are used for the MDS plots in Fig. 2. Are these spaces chosen to have the same size or the same variational energy?*

> 8. *Is the term "Hamming distance" equivalent to the excitation level relative to a reference determinant? If so, using the latter term would provide a more direct physical interpretation.*
>
> 9. *Several figures would benefit from improved quality. In Fig. 1(c), N is not rendered as a superscript and some numbers are obscured by the black circle. In addition, the small markers in the MDS plots appear as white dots in my PDF viewer unless zoomed in, likely due to marker boundary rendering.*

Response: We are grateful for the Reviewer's exceptionally careful reading and for pointing out so many detailed improvements. All those comments have been addressed:

1. The period after "pool set P" has been removed.
2. All occurrences of "dets" have been replaced with "determinants" throughout the manuscript.
3. Corrected to "S_z value."
4. Symbols in Eq. (4) now defined.
5. Holmes et al. (JCTC 2016) reference added.
6. The bond length ($r = 2.5$ Å) has been added to the caption of Fig. 2.
7. The MDS plots use variational spaces at the largest point in Fig. 2 (a,d,g); this is now stated explicitly in the caption.
8. We prefer "Hamming distance" as it is more precise (counting the number of differing orbital occupations), but we now clarify its relation to the excitation level in the text: Hamming distance = 2× excitation level.
9. In Fig. 1(c), the superscript in P^N has been corrected and the number labels repositioned for clarity. The white marker boundaries in the MDS plots are intentional: they ensure that individual determinants remain visually distinguishable.

> *At first glance the method appears capable of generating improved variational spaces for subsequent perturbative corrections. However, a more fair and comprehensive comparison with existing methods is necessary to properly assess the performance of the proposed approach. Details regarding computational parameters and the data used for PT2 extrapolation are also required, along with a more systematic investigation of their impact on convergence.*
> *Even aside from the concerns discussed above, the scope and significance of the results currently presented do not appear sufficient for publication in Nature Communications. I therefore recommend that the manuscript would be more appropriate for more technically oriented journals, such as JCP or JCTC, after the above concerns have been adequately addressed.*

Response: We appreciate the Reviewer's thorough assessment and take the recommendation seriously. We believe the substantially revised manuscript, detailed in six sections in the Supplementary Information, fully addresses the concerns raised:

The revised benchmarks demonstrate that TrimCI systematically outperforms SHCI as a *complete method* at the var+PT2 level. On the Cr_2 cc-pVDZ-DK CAS(28e, 76o) benchmark (SI S6)—one of the most challenging systems from Larsson et al. (JACS 2022)—TrimCI achieves state-of-the-art accuracy with $\sim 10^7$ determinants that previously required $\sim 10^9$ determinants with SHCI, using 6× fewer core-hours. This is not a moderate improvement: it substantially expands the frontier of what selected CI methods can tackle in practice.

More broadly, TrimCI bridges quantum chemistry and condensed matter physics by enabling accurate treatment of strongly correlated systems—such as transition metal clusters and Hubbard models—that have traditionally been the domain of QMC, DMRG, or other methods. As a general-purpose quantum many-body algorithm of interest to many communities, the method reaches beyond the quantum chemistry community. We sincerely hope the Reviewer will re-evaluate the contribution in light of the substantially expanded evidence, and we believe Nature Communications, with its interdisciplinary readership, is the appropriate venue for this work.

Response to Reviewer 4

> *The article by Zhang and Otten proposes a new kind of selected CI methods where one starts from random determinants and then searches through the configuration space following some procedure. The work makes several claims, principal of which is that this 'Trim-CI' outperforms other variants of selected CI. There are other claims, namely that the Trim-CI calculations actually produce good results for specific systems.*
> *Selected CI is not a single method, but a family of methods, and there is great flexibility with how the various aspects: initialization, selection, extrapolation can be done. In this sense, the comparison with selected CI methods then depends rather on how the comparison method is actually performed.*

Response: We thank Reviewer 4 for the thoughtful and challenging critique, which has significantly improved the manuscript. The feedback from the Reviewer directly motivated our new randomly generated benchmark (SI S1), where the systems are hard to solve and new to all methods, and the Cr_2 large-basis benchmark (SI S6), where TrimCI is compared against the best published SHCI results. We address each point below.

> *Although the authors do not clearly specify how the "comparison" selected CI method is actually done (see below), most of the gains versus 'SHCI' seem to be because SHCI used a bad starting point (e.g. in the FeS clusters). [The comparison to the quantum selected CI work for the 4Fe-4S cluster is just comparing to a very bad selection procedure, so it's not really fair and I will not discuss it further]. But*

for these molecular problems it is not hard to choose a better starting point. Trim-CI might be claimed to be a better heuristic for molecular problems to find good starting determinants, but as a heuristic, should one not then compare to a 'good faith' use of other heuristics? The text often says that the method is information free, but this also means the method can be fooled, e.g. if one happens to have sparsely connected determinants and starts in the wrong place.

Response: The concern about whether SHCI was used fairly is entirely reasonable, and we have explicitly clarified orbitals and initial states for SHCI and conducted comprehensive new experiments to address it—Reviewers 2&3 raised the same question, and we provide detailed responses above. Here we summarize the key evidence. For Cr_2 and the Hubbard model, the comparison is fair: SHCI starts from the HF determinant in HF canonical orbitals. For [4Fe-4S], we tested SHCI with both HF closed-shell and several open-shell initializations (Fig. 5)—unless the initial state precisely matches the correct broken-symmetry spin pattern, SHCI falls far behind TrimCI. On the 10 randomly generated strong-correlation systems (SI S1), where no method can exploit prior knowledge, TrimCI outperforms SHCI on every system, requiring 33–113× fewer determinants. Finally, on Cr_2 cc-pVDZ-DK CAS(28e, 76o) (SI S6), we directly compare against the best published SHCI results in the literature.

SQD comparison. Our motivation is that SQD represents the current state of the art for practical quantum computing on chemistry problems, and the comparison provides a valuable snapshot.

TrimCI is robust to poor initialization—traditional CI-like methods are not. Being "fooled" by a poor starting point is precisely the failure mode of SHCI: for example, it gets trapped in the HF basin for Cr_2 (SI S4) and fails to capture the correct antiferromagnetic correlations on the Hubbard model (SI S5). TrimCI addresses this through its two-level trimming, which actively prunes wrong paths. The ablation study (SI S3) confirms this across multiple initialization strategies—TrimCI consistently outperforms SHCI regardless of starting point. This advantage extends to large-scale calculations (Fig. 9), and holds across all initialization strategies (Fig. 10).

Put another way, many methods rely on initialization: the comparison tensor network methods discussed also rely on initialization, and in fact, even quantum algorithms for ground-state require good initialization. Finding such better initializations is useful in practice for sure, but I suppose the question is whether that is the kind of intellectual advance that justifies publication.

Response: We appreciate the Reviewer's recognition that good initialization is broadly important. We would like to emphasize just how critical this problem is: in almost all strong-correlation problems we have tested, SHCI initialized from HF in HF orbitals, or HF and open-shell in localized orbitals, becomes completely trapped in poor basins, and neither increasing computational resources nor using multiple random starting points resolves this—the variational energy remains far from

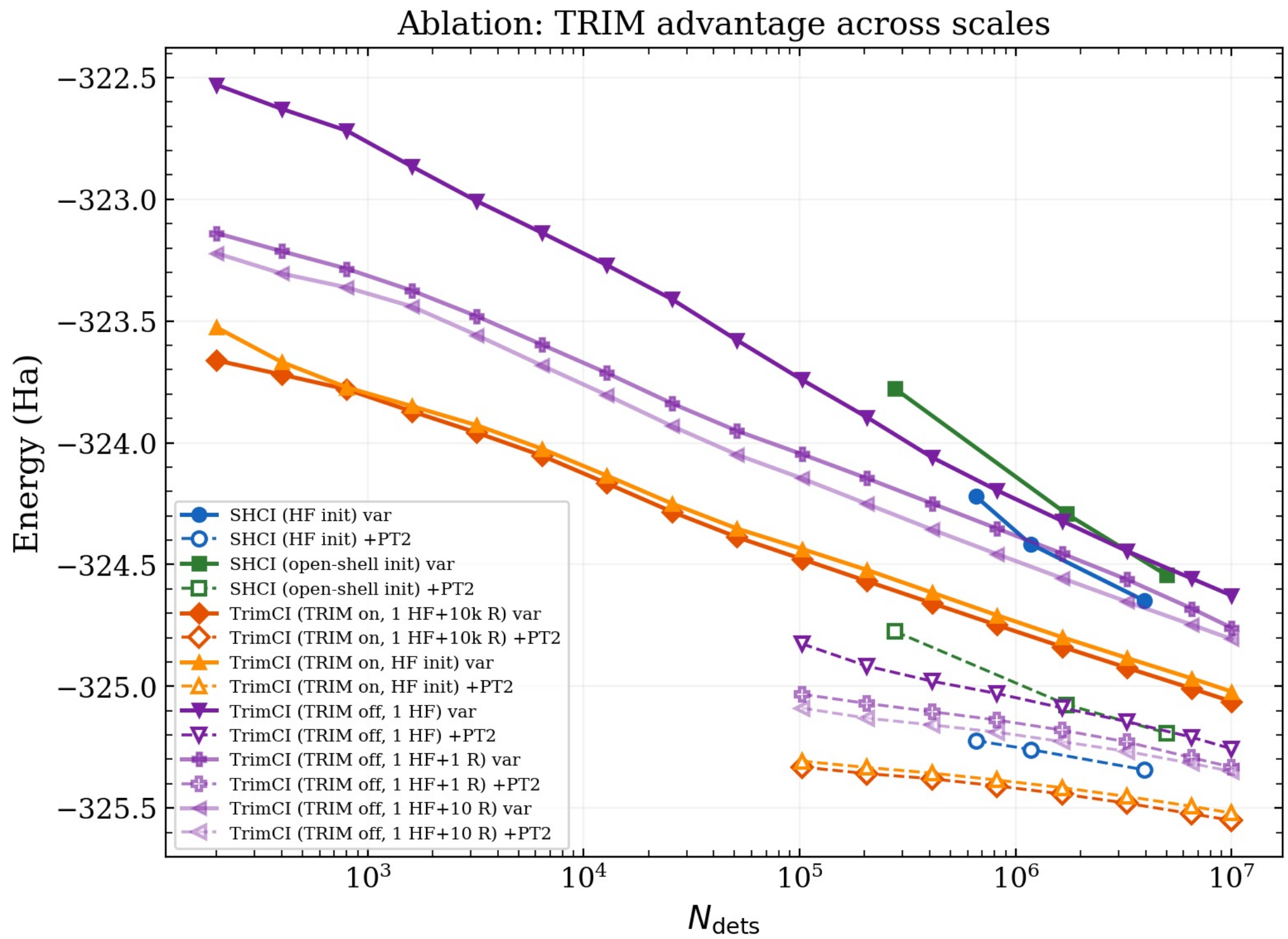

Figure 9: Ablation (SI S3): TrimCI advantage persists from 10^2 to 10^7 determinants. Solid: variational; dashed: var+PT2. TrimCI (orange) outperforms SHCI (blue/green) at all scales.

the ground state.

In contrast, TrimCI possesses a *self-correcting* mechanism: the iterative trim-and-expand cycle actively prunes unproductive determinants and redirects the search toward the physically relevant region. This works even from varied initializations, as demonstrated in the ablation study (SI S3). This self-correcting property is not merely an algorithmic convenience—it provides new insight into how quantum many-body ground states can be systematically discovered through local refinement on the Hamiltonian coupling graph.

That TrimCI's compact core set can serve as a high-quality initial state for other methods—AFQMC, DMRG, VQE, QPE—is a valuable byproduct, but the primary contribution is the search mechanism itself.

We believe TrimCI opens the door to *high-throughput* accurate quantum many-body calculations, where large numbers of systems must be solved (e.g., scanning over geometries, coupling constants, or active spaces) and manual selection of good starting points is infeasible. Before TrimCI, no selected CI method could reliably handle such scenarios without human intervention. This initialization barrier makes it extremely difficult for the broader community to adopt selected CI methods, and renders large-scale screening of strongly correlated systems essentially impossible. This capability is of broad interest across quantum chemistry, condensed matter physics, and

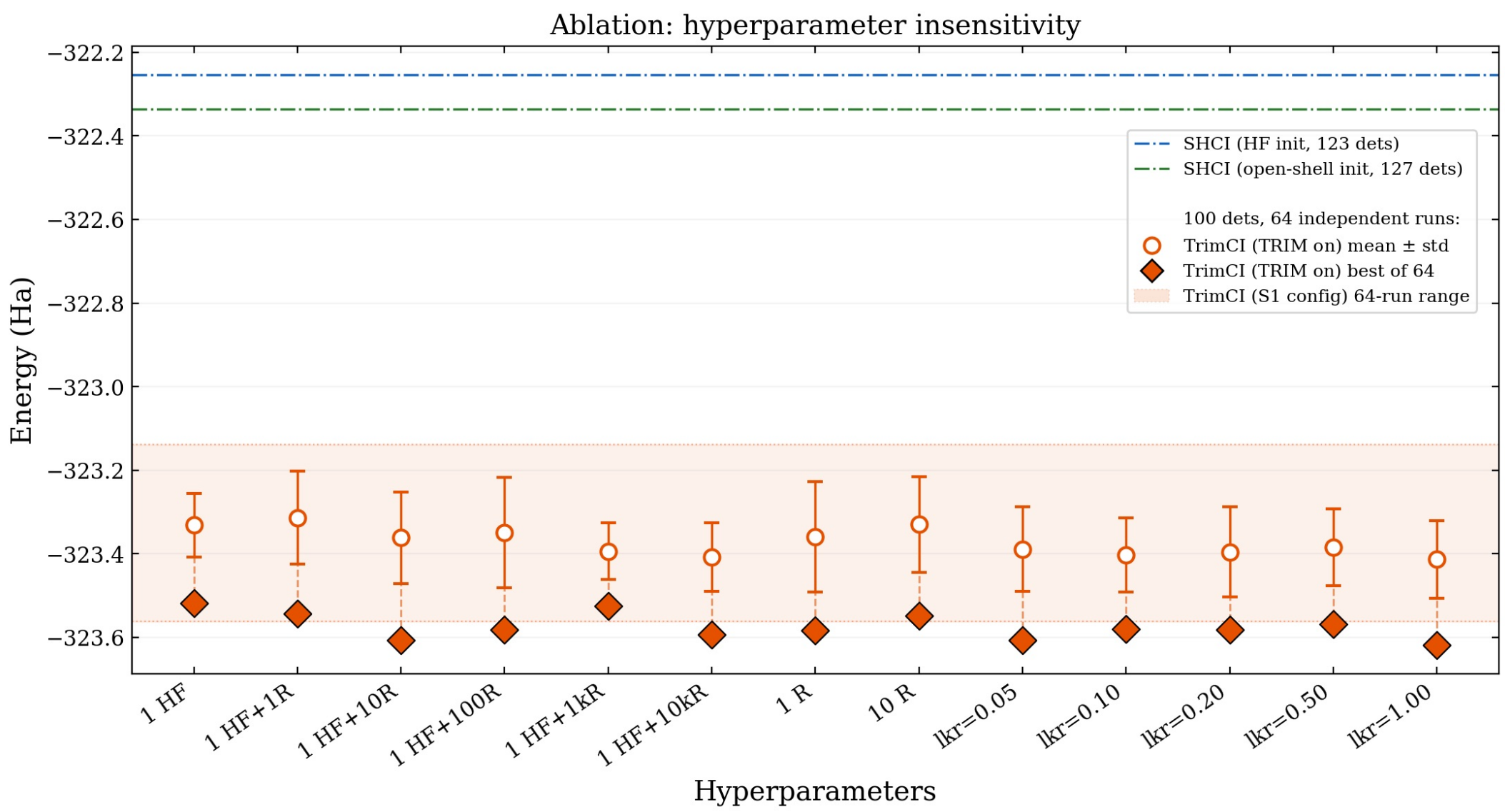

Figure 10: Ablation (SI S3): TrimCI outperforms SHCI across all initialization strategies. TrimCI initialized from HF ("1 HF") achieves comparable results to random initialization.

quantum algorithms.

Another dimension by which to assess the paper is whether the results produced are, in an absolute sense, impressive. Here I note that the authors do not compare to the state-of-the-art. For Cr_2, they compare to a minimal basis result, which could have been computed exactly ∼15 years ago, rather than to the more modern literature which uses a large basis with selected CI. For [4Fe-4S] they produce an extrapolated number that is rather far below their quoted DMRG reference number, and in poor agreement with the very recent [4Fe-4S] 'exact' benchmarks in arXiv:2601.04621. In the Hubbard model, the results beyond exact diagonalization in larger clusters require an extraordinary amount of extrapolation, which seems completely unreliable away from half-filling where there are competing orders and one is unlikely to capture the correct order with a small number of variational states. Certainly, a new method requires time for optimization and for one to learn how to apply it to reach the state of the art. However, I am simply pointing out that the method does not yet produce state of the art results, the main claim is that it is state-of-the-art by comparison to other variants of selected CI, when they use other variants perhaps without full care.

Response: This is an excellent critique that motivated one of the most important additions to the revision. We address each system in turn.

Cr_2 (large basis). In SI S6, we now apply TrimCI to Cr_2 cc-pVDZ-DK CAS(28e, 76o) at $R = 2.50$ Å—the same system and basis used in the state-of-the-art study by Larsson et al. (JACS 2022). TrimCI achieves $E_{\text{var}} = -2099.898$ Ha (17 mHa below the best SHCI E_{var}) and E_{var}+PT2 $= -2099.905$ Ha (3.4 mHa below the best SHCI+PT2). Extrapolation yields −2099.9081 Ha, within 1.5 mHa of the SHCI/DMRG reference (−2099.9096 Ha). The full computation (all variational

and PT2 points) required 40.2 hours on a single 48-core node ($\sim$ 1,930 core-hours), compared to $\sim$ 12,000 core-hours for SHCI.

[4Fe-4S]. We read arXiv:2601.04621 with great interest and used it to validate TrimCI at full scale. We found that TrimCI matches the largest UDMRG calculations ($D = 12{,}000$) at 2.56 billion determinants: for example, in the BS1 state, UDMRG($D = 12{,}000$) yields $E = -327.241697\,\text{Ha}$ while TrimCI achieves $-327.241618\,\text{Ha}$ at 2.56×10^9 determinants and $-327.242051\,\text{Ha}$ at 5.12×10^9 determinants. With PT2 corrections, TrimCI matches UDMRG($D = 12{,}000$) at 700 million determinants. Extrapolation yields $E \approx -327.244\,\text{Ha}$, in excellent agreement with the UDMRG extrapolated value. A detailed report will be presented elsewhere.

Hubbard model. At half-filling, TrimCI shows excellent agreement with AFQMC—already beyond previous selected CI performance. For the doped Hubbard model, where competing orders make convergence challenging for many methods, we plan to address this in future work.

These results demonstrate that TrimCI can produce state-of-the-art results, across molecular and lattice benchmarks.

A small but important point is that I found the paper to be lacking in details regarding the comparisons. I could not find supplementary information to explain how the SHCI was done, or indeed exactly how the AFQMC was run, what codes were used and so on and so forth. Since the paper hinges on some details, it is important that this is remedied.

Response: We thank the Reviewer for this important reminder. The revised manuscript now provides full computational details:

1. SHCI calculations use the original Arrow code; details of running are provided in SI.
2. AFQMC reference energies for the Hubbard model are cited from Qin et al. (PRB 2016); this is now stated explicitly in the main text.
3. TrimCI hyperparameters, run details, and hardware information are provided in the Supplementary Information.

Overall, I think the authors have produced a useful additional to the selected CI literature which may, with detailed and careful application, one day lead to us learning new things about physical systems, although that point has clearly not yet been reached. At the same time, I am not sure the method has the level of profundity attributed to it in the text: it is a neat and 'automatized' way to initialize a selected CI procedure, but in my reading that is the main contribution.

Response: We thank the Reviewer for recognizing TrimCI's potential and for the forward-looking perspective that it may help us learn new things about physical systems. We acknowledge that the

original submission did not sufficiently demonstrate this. In the revision, we have addressed this through extensive new experiments showing that TrimCI, as a complete workflow, is insensitive to initial conditions, requires no prior knowledge of the system, and produces state-of-the-art energies on challenging benchmarks. We believe this opens the door to tackling many new problems in strongly correlated quantum many-body physics. We sincerely hope the Reviewer will re-evaluate the contribution in light of the substantially expanded evidence, and that TrimCI will prove to be a useful tool for the community.

Closing Remarks

In response to the Reviewers' detailed feedback, we have conducted extensive new experiments to substantiate the claims of the manuscript. The revised version includes: (1) a randomly generated benchmark of 10 unknown strongly correlated systems demonstrating TrimCI's systematic advantage over SHCI under identical, prior-knowledge-free conditions (SI S1); (2) complete var+PT2 comparisons across all systems, confirming that TrimCI's advantage holds at the level of the full computational workflow (SI S1, S3, S6); (3) a state-of-the-art Cr_2 cc-pVDZ-DK CAS(28e, 76o) benchmark where TrimCI surpasses the best published SHCI results using 6× fewer computational resources (SI S6); (4) physical validation through wavefunction analysis and spin-correlation observables (SI S4, S5); and (5) robustness analysis and ablation study isolating the contributions of each algorithmic component (SI S2, S3).

Through these substantial experiments and our detailed responses to each Reviewer's concerns, we have demonstrated that TrimCI systematically outperforms SHCI at both the variational and var+PT2 levels across all benchmarks tested. The revised manuscript shows that TrimCI offers a qualitatively new approach to quantum many-body computation—enabling accurate ground-state solutions without prior knowledge, substantially expanding what selected CI methods can achieve in practice, and opening the door to high-throughput studies of strongly correlated systems. We believe the interdisciplinary nature of this contribution, bridging quantum chemistry, condensed matter physics, and quantum algorithms, makes Nature Communications the appropriate venue, and we hope that TrimCI will prove to be a valuable tool for researchers across these communities.

Sincerely,

Hao Zhang
Matthew Otten

Point-by-Point Response to Reviewers (Round 2)

"From Random Determinants to the Ground State"

Manuscript ID: NCOMMS-25-95448

We thank the reviewers again for their time in reviewing the manuscript and for their thoughtful comments and questions. The point-by-point response to each reviewer follows separately below.

Response to Reviewer 1

The authors have taken on board the previous suggestions seriously, and performed additional studies to evidence quite conclusive responses. It is interesting that the main driver of the advantage seems to be the fact that the random local-then-global sampling strategy of TRIM seems to be the primary mechanism of advantage, rather than the anticipated impact of the random initialization. This emphasises the importance of this broader exploration approach compared to sCI methods generally. I have been convinced in the response by the value that the TrimCI procedure provides over (e.g.) SHCI and related selected CI approaches as the SOTA in strongly correlated molecular systems. Whether this surpasses the sufficiency requirement of the journal is a subjective question, but I believe that it can find broad utility and impact, and would be happy to recommend publication.

Response: We are very grateful to the Reviewer for the recommendation for publication, for the recognition that TrimCI "can find broad utility and impact," and for recognizing the value TrimCI provides over selected CI approaches as the SOTA in strongly correlated molecular systems.

We also want to thank the Reviewer for the illuminating questions, which were very helpful. In particular, the framing question on what fundamentally drives the compactness leads to a deeper and sharper understanding of the TrimCI method.

As a small point, while the reply says that the best-of-64 runs were taken for all results in the ensemble selection procedure, these important simulation details seem to be omitted from the main text. While I appreciate that the details of the simulation procedures are in the SI, enough of the details which impact upon an understanding of the method should be included in the main text to faithfully judge the results and simulation procedure.

Response: Agreed. Thanks for the suggestion. We have added a sentence to the Algorithm section in the main text stating that 64 independent initializations running up to 100 determinants is a good default, and pointing readers to SI S1, S2, and Table 1 for details.

Response to Reviewer 2

The Reviewer raises eight points. Points 3 and 5–8 are about detail-level refinements (data presentation, linking SI and main text, writing, the Cr_2 binding curve, and the practical scale of determinants), and we address each below. The core of the review sits in points 1, 2, and 4, and all three reduce to a single question about the orbital basis. Before going point-by-point we want to address that question head-on.

At the center sits the reading of the $\sim$0.1 Ha spread in Fig. SI.1 of the Supplementary Information (top five panels) at $N_{\text{det}} = 10^7$ as evidence that TrimCI "is far from convergence with respect to the orbital representation". This single reading drives the "not a qualitative advance beyond the SOTA in treating strongly correlated systems" assessment of point 1, the "orbital basis is implicit prior knowledge" argument of point 2, the "the extrapolation procedure cannot recover a unique and reliable FCI limit" concern of point 4, and the concluding remarks that hold "convergence, robustness with respect to orbital representation" concerns as unresolved.

This reading misplaces the focus. The subject of Fig. SI.1 is not whether TrimCI can be driven all the way to the FCI limit on these scrambled Hamiltonians within a fixed determinant budget; it is, given a problem (the FCIDUMP), whether TrimCI finds a more compact core wavefunction than other methods do. The five scrambled-basis curves in Fig. SI.1 were generated by applying an independent random $O(36)$ rotation $V^{(r)}$ to the one- and two-electron integrals of the $[Fe_4S_4]$ Hamiltonian; the orbitals in each rotated basis are uniform mixtures of the original localized molecular orbitals, with no localization, no good ordering, and no resemblance to a physically motivated basis. Thus it is not expected to converge to FCI within the typical number of determinants used in the community, since the orbital basis is fully random. The purpose of this stress-test benchmark is to ask whether, in this deliberately hard problem, TrimCI still finds a more compact core wavefunction; we find that it does. Fig. 1 visualizes how random the orbitals are for one of the scrambled problems (`rot_01`): each row i is one new-basis orbital and each column j is one of the original 36 LMOs, with the cell colored by the squared rotation-matrix element $|V_{ji}|^2$. On top of that starting point, TrimCI's orbital optimization was *deliberately turned off* so that TrimCI and SHCI would see exactly the same one-electron basis in the variational expansion. The 0.1 Ha is a property of that adversarial setup, not of TrimCI's production run.

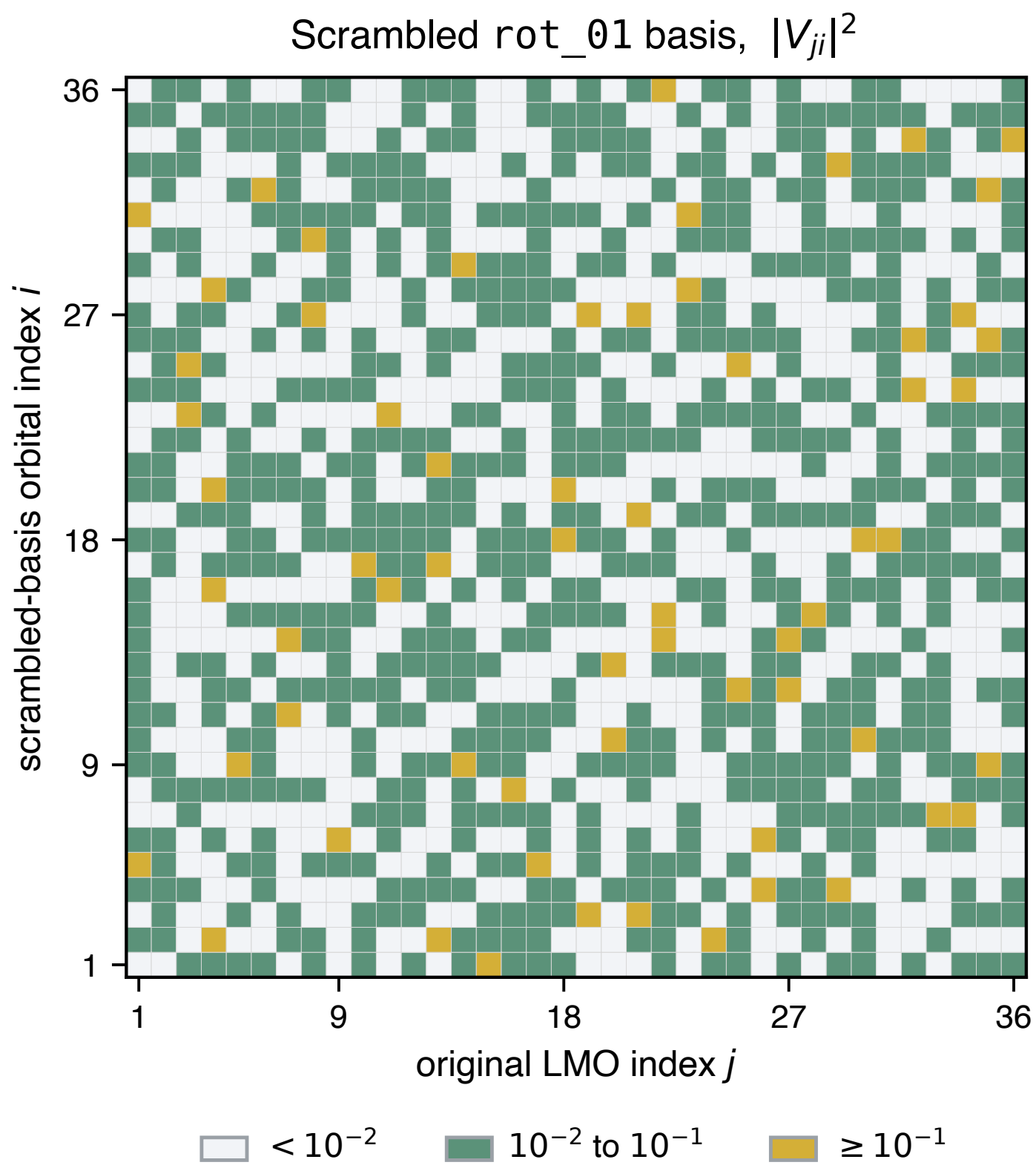

Figure 1: The scrambled `rot_01` basis used in SI S1. Rows are the 36 orbitals of the new basis; columns are the 36 original [Fe_4S_4] localized molecular orbitals. Each cell is colored by $|V_{ji}^{(\mathtt{rot\_01})}|^2$ in three tiers (white $< 10^{-2}$, green $10^{-2} - 10^{-1}$, gold $\geq 10^{-1}$). The five scrambled orbitals look qualitatively similar (see SI S7.2 for the corresponding plots). This is the basis TrimCI and SHCI were asked to work on.

That being said, the Reviewer also recognizes that this basis-dependence is not specific to TrimCI but a well-known challenge across the field:

> *"Importantly, the results reinforce a well-known limitation shared across approximate FCI methods (including SCI, DMRG, and FCIQMC), namely the strong dependence of convergence on the choice of one-electron basis. This directly challenges the interpretation of the method as 'prior-knowledge-free'."*

Motivated by this observation and to address the Reviewer's concern about orbital-basis dependence head-on, we ask:

> *starting from a fully scrambled basis, can TrimCI rotate the orbitals back to one in which the variational expansion supports an FCI extrapolation? And do the standard orbital-optimization workflows do the same?*

A clean answer to this question speaks directly to the basis-dependence concern (whether TrimCI relies on good orbitals as input), to the convergence behavior towards FCI extrapolation (whether TrimCI can rotate the initial orbitals to a basis on which the variational expansion supports an FCI extrapolation), and to the practical determinants scale at which the production workflow needs to operate. Beyond TrimCI, the same question is a useful diagnostic for other many-body methods (e.g., SCI, DMRG), since the basis-dependence challenge is shared across the field.

We designed new experiments to answer this question and report the results in SI S7. For TrimCI, the answer is yes: despite fully scrambled orbitals, for all five scrambled Hamiltonians, the TrimCI workflow with *orbital optimization turned on* (alternately optimizing orbitals and the 100-determinant wavefunction for 10 iterations) converges to a narrow spread of 0.008 Ha across the five rotations at $N_{\text{det}} = 10^7$, compared to ∼0.1 Ha previously with orbital optimization off. Linear and quadratic $E_{\text{var}}+E_{\text{PT2}}$ vs $|E_{\text{PT2}}|$ extrapolations of the five trajectories place the TrimCI FCI estimate in the range −327.240 to −327.249 Ha; for comparison, UDMRG (Zhai et al., arXiv:2601.04621) reports the three broken-symmetry FCI states of $[Fe_4S_4]$ in the range −327.244 to −327.250 Ha, and the two ranges overlap closely. We also show that at $\sim 10^9$-determinant scales the TrimCI FCI extrapolation agrees with the UDMRG extrapolation to within 0.2 mHa on the BS1 production run (see SI S7 and S8).

In contrast, the standard SCI and DMRG orbital-optimization workflows both fail to rotate the scrambled basis back: the orbitals they arrive at are far from ideal, leaving a residual variational error of 0.4–1.9 Ha from FCI and no extrapolation to the FCI limit. Specifically, despite using a much larger inner-CI wavefunction than TrimCI does (roughly 600,000 determinants per orbital-optimization cycle, compared to TrimCI's 100), SHCI's orbital optimization oscillates across all five scrambled Hamiltonians `rot_01`–`rot_05` and on `rot_03`–`rot_05` shows almost no descent from the initial energy; the subsequent expansion at $N_{\text{det}} = 7$–30 million determinants lands at −325 to −327 Ha. We also tested DMRG-CASSCF using PySCF and Block2, with a comparably large inner-CI Hilbert space (∼36 million parameters at $M = 500$); it rotates the basis substantially over twelve macro iterations ($\|U - I\|_F = 1.99$ at macro 12) but its energy oscillates in the band $[-325.80, -325.52]$ Ha without net descent. More details are in the point-by-point below and in SI S7.1–S7.4.

Taken together, the main text and the SI, including both the previous sections and the new SI Sections S7–S8, show that TrimCI consistently identifies a much more compact ground-state-relevant subspace across a broad range of systems, orbital choices, and even fully scrambled bases. This compact subspace provides the orbital gradient needed to rotate toward a favorable basis, on which the variational expansion converges rapidly to the FCI limit. This answers the Reviewer's core question.

1. Noteworthy results are primarily technical in nature. *I would like to thank the authors for performing additional calculations, which provide additional benchmarks and technical refinements over the original submission, particularly in the numerical assessment of the TrimCI method and in clarifying aspects of its implementation. The additional tests in the Supporting Information help to better contextualize the behavior of the method under orbital transformations. With the new results, the authors demonstrate that TrimCI is capable of providing a variational space that consistently yields lower energies than the HCI algorithm, both before and after the perturbative correction.*
However, they do not convincingly establish a qualitative or conceptual advance beyond the current state of the art in treating strongly correlated systems. The manuscript does not provide clear evidence that the method enables new physical insights or qualitatively improved predictions for chemically relevant observables.

Response: We are grateful for the Reviewer's recognition that TrimCI yields a variational space which consistently produces lower energies than HCI, both before and after the perturbative correction. We disagree, however, that the new results are only technical. We sketch the conceptual picture below.

TrimCI is qualitatively different from how prior CI methods search the Hilbert space. The classical CI approaches either solve the problem in the full Hilbert space (FCI) or restrict it to the subspace spanned by selected orbitals (CASCI); both become infeasible once the orbital and electron counts grow. Selected CI grows the variational space outward from a reference determinant through perturbative or heat-bath selection rules. This selected-CI family works in regimes with a good known reference, typically the HF determinant. It fails outright for strongly correlated systems where we lack such a known reference: in iron–sulfur clusters and in the Hubbard model, the HF reference is qualitatively wrong, and the determinants that the heat-bath / PT2 rules add at each step are heavily biased toward that wrong reference, so the variational space grows in the wrong direction. A genuinely new search strategy is needed.

The trim mechanism is exactly such a strategy. It views the problem as a search in a large graph, where each node is a determinant and the edge between two nodes encodes the corresponding Hamiltonian matrix element H_{ij}. The expansion step enlarges the candidate pool through local moves on this graph; the trimming step then prunes the unimportant determinants and keeps the ones carrying the dominant ground-state weight. Iterated together, expansion and trimming locate a highly compact, ground-state-relevant subspace directly, without giving any node a privileged role as a reference.

The mechanism extends further: it remains effective under arbitrary, even fully scrambled, orbital bases (SI S1, S7). On a fully scrambled basis, where the localized structure or any other physically relevant structure that other methods rely on is destroyed, TrimCI still finds a highly accurate, compact 100-determinant core wavefunction. The reduced density matrices computed from this wavefunction then carry the orbital gradient needed to rotate the bad orbitals back to a good basis, on which the subsequent variational expansion converges rapidly and extrapolates

to the FCI limit. TrimCI therefore relies on neither a good initial orbital basis nor a good initial reference.

The trim mechanism gives a way to identify the ground-state-relevant subspace starting from random determinants, in fully scrambled orbital bases, in an exponentially large Hilbert space, without prior knowledge of good references or good orbitals. We think this is a physical insight, not just a numerical refinement, and it may push the current state of the art for strongly correlated quantum many-body systems forward.

2. Limited significance with respect to the broader field. *In its current form, the work does not reach the level of general significance expected for publication in this journal. The central claims of improved efficiency and robustness of a selected CI strategy are of interest within a small sub-community in the electronic structure theory area (the Selected-CI community) but remain within the scope of ongoing methodological developments.*

Importantly, the results reinforce a well-known limitation shared across approximate FCI methods (including SCI, DMRG, and FCIQMC), namely the strong dependence of convergence on the choice of one-electron basis. This directly challenges the interpretation of the method as "prior-knowledge-free".

The authors show that the trimming step, which is the the key component of the proposed algorithm, indeed plays a crucial role in optimizing the variational space (Fig. 4 in the SI). Also, from the many-body standpoint it is true that no trial wavefunction is required in TrimCI as compared to other SCI strategies. At the same time, however, the results indicate a non-negligible dependence on the initial random composition of the variational space, as the final energy differences between different initial conditions significantly exceed chemical accuracy. Furthermore, the choice of orbital shapes constitutes an implicit form of prior knowledge that significantly affects convergence. This is what for example we also do in DMRG (localizations, reordering via Fiedler), and those I am afraid belong to what people define technical aspects that are tightly bound to the methodological framework.

The results in Fig. SI.1 illustrate that different orbital rotations lead to substantially different convergence behavior, consistent with this well-established issue. While the authors use the figure to argue the superiority of their method against other SCI, they do not sufficiently acknowledge that TrimCI exhibits large differences even at large determinant numbers, which is a significant issue in the context of strongly correlated systems. For example, energies obtained for different orbital rotations of the same Hamiltonian differ at the TrimCI level by approximately 0.1 Ha even at 10^7 determinants (the highest value presented). This indicates that the method is far from convergence with respect to the orbital representation. What would it take to converge them? Would it be possible with their current algorithm to go say to 10^{10} determinant? Would extrapolation of those 5 curves yield a consistent FCI limit?

With the available data, TrimCI does not appear to overcome key bottlenecks in the field, but rather exhibits similar limitations with somewhat improved numerical performance.

Given that chemically relevant energy differences are typically on the order of milli-Hartree, such discrepancies are too large to support reliable predictions of energy gaps, spin-state ordering, or other observables. This assessment supports my view that the manuscript is more appropriate for a specialized journal in quantum chemistry or electronic structure theory (JCTC or JCP) rather than a broad-scope venue.

Response: We are grateful to the Reviewer for the perspective shared in this point on the family of approximate FCI methods and the role of one-electron basis dependence. While we understand how important the orbital basis is, we find the observation that basis-dependence is a well-known challenge across SCI, DMRG, and FCIQMC both insightful and informative; this motivates the orbital-recovery experiment in SI S7.

As summarized in the beginning, with TrimCI's orbital optimization on, the five scrambled-basis trajectories converge to a 0.008 Ha spread at $N_{\text{det}} = 10^7$ and extrapolate into the FCI limit; SHCI's and DMRG-CASSCF's orbital optimizations, run with much larger inner-CI subspaces or variational parameters on the same scrambled basis, do not. Below we discuss in detail; more details in SI S7.

Starting from a 100-determinant core wavefunction on each scrambled Hamiltonian, ten cycles of TrimCI orbital optimization drop the energy by ∼3.5 Ha (Fig. 2a). The basis those ten cycles produce is good enough that the subsequent expansion converges to a narrow band: variational energies between −327.228 and −327.236 Ha, and PT2-corrected energies between −327.232 and −327.239 Ha, at $N_{\text{det}} = 10^7$ (Fig. 2b, filled markers). For reference, the hollow markers and dashed lines are results from SI S1, with orbital optimization *disabled* – the setup the reviewer referred to, which gave the ∼0.1 Ha spread. Linear and quadratic $E_{\text{var}} + E_{\text{PT2}}$ vs $|E_{\text{PT2}}|$ extrapolations of the five trajectories agree well with UDMRG FCI extrapolation results in the literature.

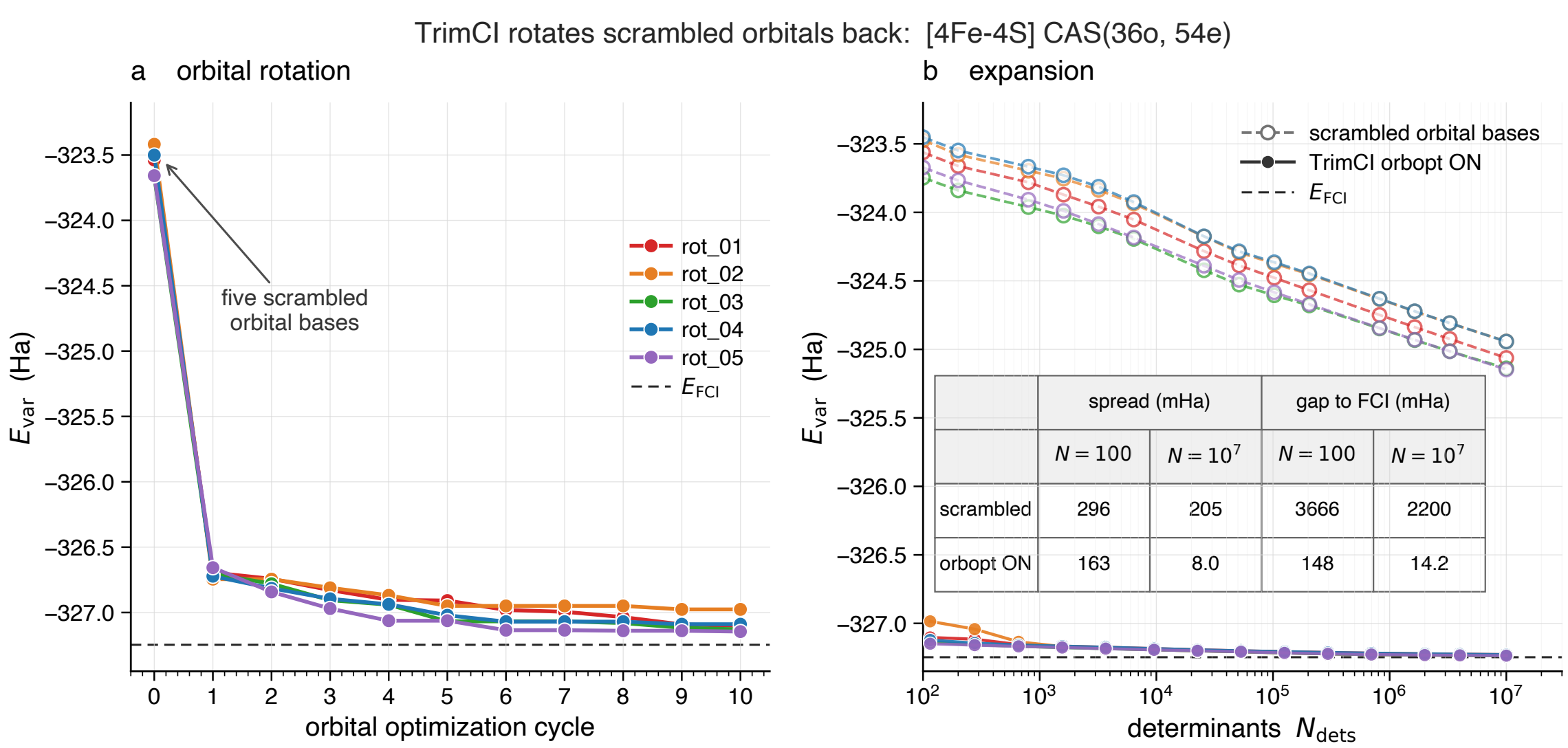

Figure 2: From SI S7.1. **(a)** The 10-cycle TrimCI orbital optimization on each of the five randomly rotated $[Fe_4S_4]$ Hamiltonians descends by ∼3.5 Ha from a 100-determinant core wavefunction. **(b)** The five Phase 1+2 trajectories, starting from each optimized basis, converge to a 0.008 Ha spread at $N_{\text{det}} = 10^7$. Hollow markers / dashed lines are from SI S1 for comparison.

To see what the recovered basis actually looks like, we plot $|C_{ji}|^2$, the squared weight of each new-basis orbital on each original LMO (Fig. 3), where $|i\rangle_{\text{new}} = \sum_j |j\rangle_{\text{LMO}}\, C_{ji}$. At cycle 0, the panel

shows the fully scrambled orbitals of SI S1: every new orbital is a uniformly random mixture of the LMOs. By cycle 10 the picture is mostly white background with a sparse set of gold landmarks: each recovered orbital has localized onto a small handful of LMOs. This pattern of basis recovery holds independently across all five scrambled orbitals. Notably, the energy convergence curves of Fig. 2b indicate that the orbitals produced by TrimCI orbital optimization are even better than the original localized orbitals shipped with the FCIDUMP from the literature, which is consistent with the cycle-10 visualization showing that the recovered orbitals have acquired a structure of their own.

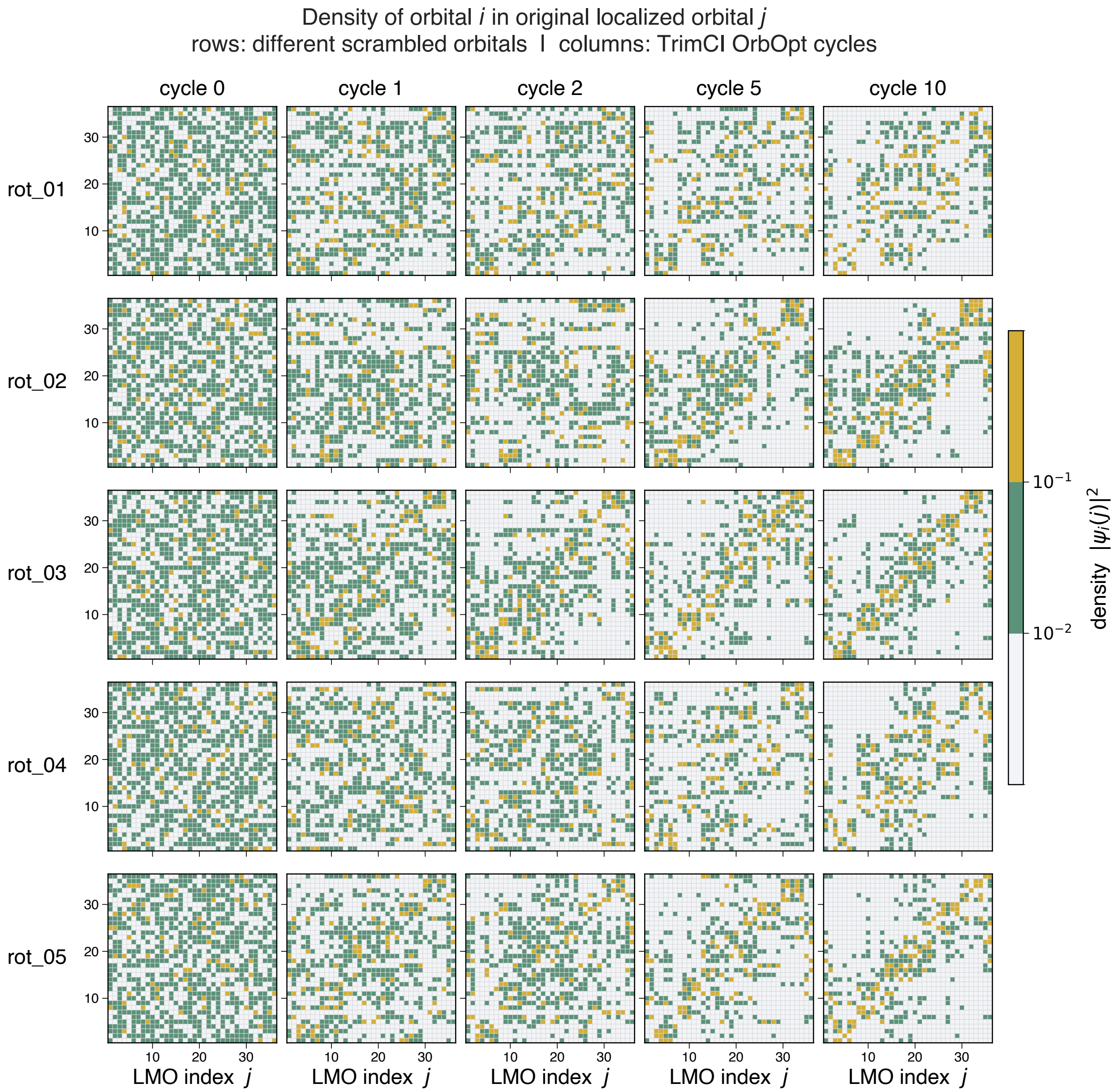

Figure 3: From SI S7.2. Density of orbital i in original localized orbital j as a function of TrimCI Phase 0 cycle, for each of the five scrambled orbitals (rows). Columns are snapshots at cycle 0, 1, 2, 5, and 10. Cells are coloured by $|C_{ji}|^2$ in three tiers (white $< 10^{-2}$, green 10^{-2}–10^{-1}, gold $\geq 10^{-1}$). The same pattern of basis recovery holds across all five scrambled orbitals.

For comparison, we run SHCI's orbital optimization on the same five scrambled Hamiltonians with $\sim 600{,}000$ inner-CI determinants per cycle, much larger than TrimCI's 100. SHCI's energy oscillates without monotonic descent on every panel and on `rot_03`–`rot_05` barely moves from the initial scrambled energy (Fig. 4a). The subsequent variational expansion on the SHCI-optimized basis (Fig. 4b) lands at -325 to -327 Ha, with 0.4–1.9 Ha residual error to FCI – two orders of magnitude worse than what TrimCI achieves on the same starting basis. In panel (a), each blue

curve traces one problem's per-cycle SHCI inner-CI energy and the matching red curve shows TrimCI's; in panel (b), the subsequent variational expansions on each method's optimized basis are shown side by side, with the TrimCI-basis curves reaching the FCI limit while the SHCI-basis curves are well above it.

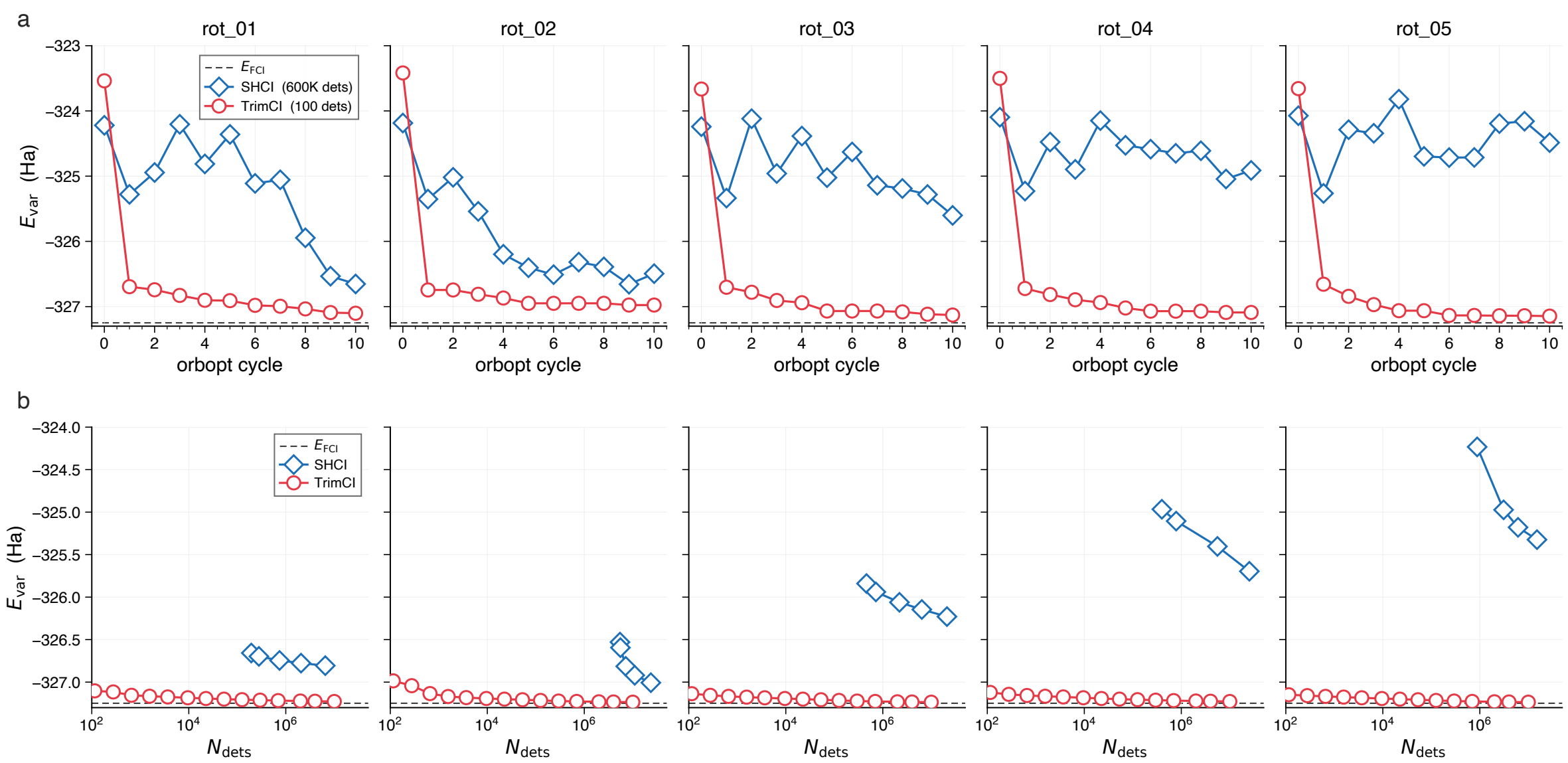

Figure 4: From SI S7.3. SHCI orbital optimization on the same five scrambled $[Fe_4S_4]$ Hamiltonians (blue) vs. TrimCI Phase 0 orbital optimization (red). **(a)** Per-cycle inner-CI energy during 10 orbital-optimization cycles. SHCI oscillates without monotonic descent on every panel; `rot_03`–`rot_05` barely move from the initial scrambled energy. TrimCI quickly converges to a good orbital basis. **(b)** Subsequent variational expansion on each method's optimized basis: TrimCI converges to the FCI limit on every panel; SHCI's expansion lands at −325 to −327 Ha with 0.4–1.9 Ha residual error.

DMRG-CASSCF exhibits a similar problem. On the same scrambled `rot_01` Hamiltonian we ran DMRG-CASSCF using PySCF and Block2 with intra-active rotation enabled, ∼36 million inner DMRG parameters at $M = 500$, and twelve macro iterations. The optimizer is continually adjusting the orbitals: $\|U - I\|_F$ grows from 0.73 at macro 1 to 1.99 at macro 12 (Fig. 5b). The energy, however, does not come along – it oscillates in $[-325.80, -325.52]$ Ha across all twelve macros and never improves on the initial energy at macro 0 (Fig. 5a). The orbital gradient computed from the inner DMRG 1/2-RDMs on a scrambled basis simply does not point usefully toward the ground-state basis. DMRG appears more severely sensitive to orbital choice than SCI on this benchmark, which we think attributes to the MPS ansatz's sensitivity to orbital ordering and localized structure.

On the final basis we further ran a DMRG sweep up to $M = 4000$ (Fig. 5c); E_{var} has only descended to −326.363 Ha, still ∼0.9 Ha above the FCI limit, which shows that DMRG-CASSCF cannot rotate the scrambled orbitals back to one that supports an efficient variational expansion.

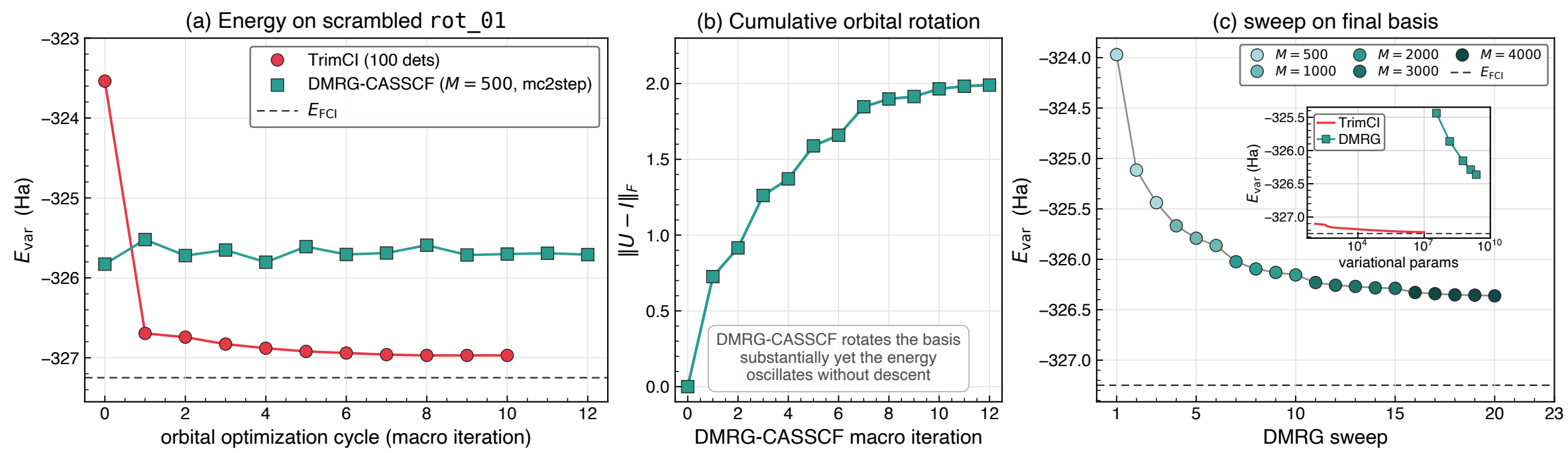

Figure 5: From SI S7.4. DMRG-CASSCF using PySCF and Block2 (twelve macros, intra-active rotation enabled, green) vs. TrimCI Phase 0 (red) on the same scrambled `rot_01` Hamiltonian. **(a)** Energy vs. iteration. DMRG-CASSCF oscillates in the band $[-325.80, -325.52]$ Ha across all twelve macros, never improving on the initial DMRG energy at macro 0; TrimCI descends to ~ -327 Ha within three cycles. **(b)** The orbital rotation $\|U - I\|_F$ for the DMRG-CASSCF trajectory grows to 1.99, so the failure is not that the optimizer stalls; the orbital gradient computed from the inner DMRG 1/2-RDMs on a scrambled basis simply does not point usefully toward the ground-state basis. **(c)** DMRG sweep on the final orbital basis with M grown from 500 to 4000 over 20 sweeps. Even at $M = 4000$, E_{var} remains $\sim$0.9 Ha above the FCI limit. Inset: E_{var} vs. variational parameters (one CI coefficient per determinant for TrimCI; $4\, N_{\text{site}}\, M^2$ for DMRG).

In summary, we showed that TrimCI rotates a maximally bad orbital basis back to a good one, while the standard SCI and DMRG orbital-optimization workflows, with much larger inner-CI subspaces or variational parameters on the same starting basis, do not.

3. Significant concerns in data interpretation and presentation. *Several aspects of the data analysis and presentation raise concerns:*
(i) The comparison between Fig. 2(d) in the main text and Fig. SI.1 reveals inconsistencies. The main text reports an energy of approximately -326.8 *Ha for* 10^3 *determinants, whereas the SI data only reach about* -325.6 *Ha even at* 10^7 *determinants. This discrepancy suggests a strong dependence on orbital choice (e.g., localized vs. delocalized orbitals), which is not sufficiently emphasized in the main conclusions.*
(ii) The use of phrases such as "in the narrow range of -323.5 *to* -323.6 *Ha" is potentially misleading. In the context of strongly correlated quantum chemistry, a spread of 0.1 Ha is extremely large and cannot be considered a small variation. I want to repeat and stress that in quantum chemistry, especially for strongly correlated systems, such large value is not acceptable for any valuble prediction/validation. Most of the magnetic, optical and catalytic properties occur within a few milli-Ha.*
(iii) The manuscript would benefit from a more transparent discussion of variance reduction as a function of the number of determinants, which is currently not clearly addressed.
These issues collectively weaken the reliability of the interpretation and should be addressed in a more rigorous and transparent manner.

Response: On (i): we are aware of the effect of orbital choice on the energy. When we compare TrimCI to other selected-CI methods, we deliberately fix the orbital basis so that both methods see exactly the same problem (FCIDUMP). This is a controlled experiment that isolates the orbital effect from the method effect, so that the comparison is a fair test of whether TrimCI can find a more compact wavefunction than the others on the same Hamiltonian.

In Fig. 2(d), the orbital basis is the original localized orbitals from the FCIDUMP in the literature, as stated in the first paragraph of the Main Results. In SI S1 (Fig. SI.1), the purpose is to ask whether TrimCI can still find a more compact wavefunction on a maximally uninformative (scrambled) basis, so we apply a random $O(36)$ rotation to the FCIDUMP file and feed the same scrambled FCIDUMP to both TrimCI and SHCI. Within each figure the orbital basis is held fixed across methods by design; the difference between the two figures is that the basis itself is different, which is what produces the energy gap the Reviewer noted. To make this controlled-experiment intent explicit, we have added one sentence to the first paragraph of the Main Results (after the benchmark systems introduction): "In the following, we use the same orbitals to compare with selected CI, to isolate the effect of the orbital basis." We have also stated the construction more explicitly in the SI S1 introduction and the Fig. SI.1 caption.

On (ii): To avoid confusion, we have removed "narrow". We have also added a clarifying paragraph at the end of the SI S1 Discussion: "We emphasize that the subject of study in this section and the sections below is algorithmic behavior, which we probe by controlled experiments on scrambled orbital bases. The absolute energies reported here should therefore not be confused with TrimCI's production accuracy: in the production setting, where orbital optimization is enabled, the residual energy is much smaller and lies in the milli-Hartree range relevant for chemical and physical prediction."

On (iii): We have added a table in SI S7.1 reporting the mean and standard deviation of E_{var} across the five scrambled orbitals at representative N_{det} values: the mean descends from -323.556 to -327.233 Ha and the standard deviation across the five scrambled problems shrinks from 105.5 to 4.4 mHa as N_{det} grows from 100 (before orbital optimization) to 10^7.

__4. Uncertainty in extrapolation to the FCI limit.__ As in other selected CI approaches, the authors rely on extrapolation schemes to estimate the FCI limit. In Fig. SI.1 (top five subplots), the same Hamiltonian is considered under different orbital rotations, yet the energies at 10^7 determinants still differ by approximately 0.1 Ha. In an ideal scenario, extrapolation of these curves should yield consistent estimates of the FCI limit, independently of the orbital representation. Given the substantial spread at the largest determinant space explored, it is unclear whether the extrapolation procedure can recover a unique and reliable limit. I encourage the authors to explicitly report extrapolated values for these cases and assess their consistency across different orbital choices. Without such analysis, the reliability of the extrapolation remains uncertain.

Response: We replied above and detailed in SI S7. For reference, we repeat here: linear and

quadratic $E_{\mathrm{var}}+E_{\mathrm{PT2}}$ vs $|E_{\mathrm{PT2}}|$ extrapolations of the five trajectories place the TrimCI FCI estimate in the range −327.240 to −327.249 Ha; for comparison, UDMRG (Zhai et al., arXiv:2601.04621) reports the three broken-symmetry FCI states of $[Fe_4S_4]$ in the range −327.244 to −327.250 Ha, and the two ranges overlap closely. We also show that at $\sim 10^9$-determinant scales the TrimCI FCI extrapolation agrees with the UDMRG extrapolation to within 0.2 mHa on the BS1 production run.

> ***5. Reproducibility is partially supported but presentation of the Supporting Information is unclear.*** *The revised manuscript provides a substantial amount of technical detail, particularly in the new Supporting Information file. However, the organization and integration of the SI with the main text are insufficient.*
> *Individual SI sections are not clearly referenced in the main text, making it difficult to follow how specific datasets support particular claims. Improving cross-referencing and explicitly linking SI figures to the corresponding discussion in the main text would significantly enhance clarity and reproducibility.*

Response: Thanks for the suggestion. We have added explicit SI cross-references for every section in the main text: SI S1 (controlled-experiment design with scrambled-orbital benchmarks), SI S2 (multi-run robustness), SI S3 (trim-mechanism study), SI S4 (Cr_2 wavefunction analysis), SI S5 (Hubbard order-parameter analysis), SI S6 (Cr_2 cc-pVDZ-DK large-basis benchmark), SI S7 (orbital recovery from a fully scrambled basis), and SI S8 (empirical GPU scaling law). Each reference is placed at an appropriate place to improve the clarity and reproducibility of the manuscript.

> ***6. Concerns regarding writing style and clarity.*** *The revised material exhibits issues in clarity and consistency of presentation. Certain passages (e.g., in Section S1) introduce concepts such as " High–throughput studies – scanning phase diagrams across coupling parameters, screening candidate materials, or exploring libraries of drug–like molecules – generate large numbers of distinct electronic structure problems, making it impractical to hand–craft initialization strategies for each one ", that are not clearly connected to the core results of the paper. In addition, there is an eccessive use of hyphen symbols (see sentence above) and repeated sentences (for example, " The scenarios motivating this benchmark – high–throughput screening, phase–diagram sweeps, and novel materials with unknown electronic structure – share a common feature ").*
> *While the use of language assistance tools is not inherently problematic, the current writing occasionally prioritizes broad or generic phrasing over precise scientific communication. This reduces readability and, in some cases, obscures the connection between motivation and results.*
> *A careful revision aimed at improving clarity, removing redundancies, and ensuring that all statements are directly relevant to the presented work would strengthen the manuscript.*

Response: Thanks for the suggestion. Both quoted sentences have been rewritten. The opening motivation paragraph in SI S1 now lists examples more relevant for TrimCI applications (phase-diagram scans of strongly correlated lattice models, bond-distance curves of multi-reference

molecules, and families of transition-metal complexes), and the closing "Implications" paragraph no longer repeats them. We have also gone through the text to address language issues, with the aim of improving clarity, precision, and relevance throughout.

7. Thorough comparison to referenced work. *The Cr_2 benchmark result referenced by the authors originates from Larsson et al. (2022). Since the cited work's main contribution is its theoretical prediction of an accurate Cr_2 binding curve that closely matches the experimental results, it would be valuable to also examine the Cr_2 binding curve obtained using TrimCI.*

Response: Thanks for the suggestion. At the $R = 2.5$ Å shelf point (one of the hardest points on the curve), we show in SI S6 that TrimCI achieves E_{var} 17 mHa lower and $E_{\text{var}} + E_{\text{PT2}}$ 3.4 mHa lower than the largest SHCI calculation in Larsson et al. using 10^8 determinants ($\sim 10\times$ fewer determinants), with E_{extrap} within 1.5 mHa of the SHCI/DMRG reference. The binding-curve comparison of Cr_2 is interesting but beyond the scope of the current manuscript, and we will leave it as future work.

8. Minor Revision. *The authors generally use a maximum of 10^7 determinants. In few cases they go beyond that. I would suggest to add a sentence referring to the actual practical limits of TrimCI in terms of the number of determinants that can be handled, and whether this limits could be pushed further with algorithmic advances (parallelization, GPU acceleration, etc.).*

Response: In the past six months, we have implemented a GPU version of TrimCI. We report the GPU results in SI S8. The largest single-node calculation, on a 4×H200 node, reaches $N_{\text{det}} = 2.56 \times 10^9$ with a 16.7 h Davidson loop; this matches the latest state-of-the-art UDMRG calculation for $[Fe_4S_4]$ with $D = 12{,}000$. The current implementation can already handle around 10^{10} determinants on a single 8×H200 node. We then performed extensive GPU performance tests across six GPU models and determinant counts spanning 10^6 to 2.56×10^9, and extracted an empirical scaling law $t \propto N_{\text{det}}^{1.2}$ (see SI S8.3). Combining the two, the implementation can be pushed toward 10^{11}–10^{12} determinants within reasonable time and resources (roughly a day on several thousand GPUs; SI S8.4).

Concluding remarks on accuracy and physical relevance.
I would like to reiterate a central concern regarding the physical reliability of the reported results. When specific orbital representations are employed, it is typically because such prior-knowledge-based choices are expected to capture the essential physics and lead to consistent results, independently of the details of numerical convergence.
In the present case, however, the reported energies exhibit variations on the order of 0.1 Ha, which is substantial in the context of quantum chemistry. Numerical values alone are not meaningful if derived quantities, such as energy gaps between spin states, inherit uncertainties of this magnitude. A discrep-

ancy of ~ 0.1 Ha in relative energies fundamentally limits the interpretability of the results, particularly for strongly correlated systems such as Fe_4S_4, where chemically relevant energy scales are typically in the milli-Hartree range. In the case of the Cr_2 potential energy curve, if I recall correctly the dissociation energy is within a few tens of milli-Hartree, so a 0.1 Ha error would be completely unacceptable.
Furthermore, the dependence of the results on the number of determinants remains significant: increasing the variational space from 10^5 to 10^7 determinants leads to energy changes of approximately 0.2 Ha. This indicates that the method is not converged with respect to the size of the determinant space. I emphasize that this concern is not specific to TrimCI alone, but applies more broadly to the class of selected CI approaches considered here, with and without perturbative corrections. The authors have the opportunity to show whether extrapolation techniques can mitigate this issue for example applying extrapolations to the sub-figures of Fig SI.1.

Final recommendation. *In light of the points detailed above, I regret that I still cannot recommend this work for publication in its current form in Nature Communications. While the authors have made a clear effort to address the technical comments raised in the previous round, the fundamental concerns regarding convergence, robustness with respect to orbital representation, and the overall significance of the results remain unresolved.*
Given that these issues are intrinsic to the current formulation and assessment of the method, I am not convinced that further revision would sufficiently address them within the scope of this journal. I would therefore suggest that the manuscript may be more appropriately considered for a specialized venue.

Response: We thank the Reviewer for the question on the orbital basis, which has pushed us to think more deeply about what TrimCI does and motivated the new experiments in SI S7.

In SI S1, our purpose was a controlled experiment: given a fixed FCIDUMP with scrambled orbitals, does TrimCI find a more compact ground-state-relevant subspace than SHCI on the same one-electron basis? Across all ten benchmark systems, the answer is yes; the comparison is about subspace compactness at a fixed orbital input, not about absolute energy convergence to the FCI limit.

The Reviewer's deeper concern is that TrimCI might still rely on a good orbital basis being provided, in which case the result would encode prior knowledge through the inputs. We address this directly in SI S7 by asking: starting from a fully scrambled orbital basis, can TrimCI rotate it back to one on which the variational expansion supports a fast FCI convergence? The answer is yes. Across the five scrambled rotations of $[Fe_4S_4]$, TrimCI's orbital optimization converges to a 0.008 Ha spread at $N_{\mathrm{det}} = 10^7$, and linear and quadratic $E_{\mathrm{var}} + E_{\mathrm{PT2}}$ vs $|E_{\mathrm{PT2}}|$ extrapolations place the TrimCI FCI estimate in −327.240 to −327.249 Ha, overlapping with the UDMRG broken-symmetry FCI range −327.244 to −327.250 Ha (Zhai et al., arXiv:2601.04621). The standard SHCI and DMRG-CASSCF orbital-optimization workflows, run on the same scrambled basis with much larger inner-CI subspaces or variational parameters, do not recover such a basis.

Therefore TrimCI does not rely on good orbitals being provided as input: starting from a maximally uninformative orbital basis, the trim mechanism produces an orbital gradient that drives the

basis to one supporting an FCI extrapolation, where the standard orbital-optimization workflows on the same starting point do not.

TrimCI offers a qualitatively new way to search the Hilbert space, achieving state-of-the-art results without relying on good reference determinants or good initial orbitals. We believe the method and the conceptual insights it provides will be of broad interest across quantum chemistry, condensed matter, materials science, and quantum information. We hope the Reviewer will reconsider in light of these new experiments and findings.

We thank the Reviewer again for helping us sharpen the story and presentation.

Response to Reviewer 4

The revised version of the paper and the response letter add some additional calculations that help to strengthen the authors' case that TrimCI is a useful method. In general I agree that the additional calculation on Cr_2 *(at one geometry, one where the HF reference is a very poor starting point) and [4Fe-4S] (not included in the paper) do show that, in practice, TrimCI performs better than SHCI starting from the HF reference. Thus, there is a path towards claiming that, amongst current implementation of SCI, and assuming one doesn't do much work on the initialization, TrimCI does achieve competitive performance, although the case for the paper is weakened by the fact that the [4Fe-4S] example included in the response is not included in the paper.*

Response: We are grateful to the reviewer for the positive evaluation on this round, in particular for the acknowledgment that TrimCI achieves competitive performance among current SCI implementations. For [Fe_4S_4], we have added detailed information in SI S8 about our largest single-node calculation: a 16.7 h Davidson run on the [Fe_4S_4] BS1 problem at $N_{\text{det}} = 2.56 \times 10^9$ using a 4×H200 node. This matches the largest published UDMRG calculation for the same system at $D = 12{,}000$ (Zhai et al., arXiv:2601.04621).

At the same time, there is still a sense of overclaiming in the writeup of the paper. TrimCI is optimizing over the initial state, something that can be (and is also sometimes) done in SHCI, as a separate procedure called the initialization, but here is defined as part of SCI algorithm itself. While the response letter states that they are in agreement with this criticism, in practice the changes to the paper are quite minimal (e.g. changing "without relying on any prior knowledge" to "without relying on prior knowledge about good initialization" is not really much of a change). For example, if you initialize SHCI by always doing a CAS calculation and picking those determinants – as has been done before – is that really so different? The practical advance is that the random search turns out, for the systems studied, to be quite a good way to find an initial set of states, and in fact may be better than other ways that have been used in the past.

I think the results achieved are now quite encouraging, and in principle, I think it is now more convincing as a publication. However, I would discourage the authors from retaining this language about lack of prior knowledge compared to prior algorithms, because it really depends on what you define to be "inside" the SCI algorithm as opposed to "outside" of the algorithm. At the very least, the other referee reports show that this emphasis is controversial. Rather the authors should focus on the fact that the heuristic TRIM procedure is good at capturing certain patterns of CI coefficients in determinant space that are not solely centered around the HF determinant.

Response: We are grateful to the reviewer for the recognition that the results are "now quite encouraging" and "more convincing as a publication," and especially for the remark that the TRIM procedure "turns out, for the systems studied, to be quite a good way to find an initial set of states, and in fact may be better than other ways that have been used in the past." This is a very high evaluation of our work, and we deeply appreciate it.

Now we address the reviewer's remaining concern regarding the language of "lack of prior knowledge." The concern rests on the following reasoning: SHCI (and related methods) can in principle be initialized in various ways, including by doing a CAS calculation and picking the leading determinants from it.

We partially agree with this view. For problems with a clearly identified active space, a CAS-based initialization for SHCI is a viable strategy.

However, the above CAS-initialization route is limited. It requires a meaningful small active space within which a full CI can be solved to identify the leading determinants. On [Fe_4S_4] CAS(36o, 54e), it is unclear to find such a meaningful smaller active space.

One may argue that DMRG-CASSCF can be used to handle larger active spaces. However, DMRG itself also requires good initial states and good orbitals for problems like iron-sulfur systems. We demonstrate this with the following experiment.

DMRG-CASSCF on a scrambled [Fe_4S_4] basis (SI S7.4). The test problem is one of the [Fe_4S_4] Hamiltonians studied in SI S1, whose one- and two-electron integrals have been scrambled by a random $O(36)$ rotation. We run DMRG-CASSCF on this scrambled basis using PySCF's `mc2step` with SU(2)-DMRG at $M = 500$ as the inner ground-state solver (∼36 million inner parameters) and intra-active rotation enabled. The orbitals do rotate substantially ($\|U - I\|_F = 1.99$ at macro 12), but the energy oscillates without net descent and never improves on the initial DMRG energy; pushing M to 4000 on the final basis still leaves $\sim 0.9\,\text{Ha}$ above the FCI limit. TrimCI on the same scrambled starting point, with a 100-determinant inner wavefunction, drives the basis to one supporting fast variational convergence. With data up to only $N_{\text{det}} = 10^7$ (a quick test), the FCI extrapolation already lies in -327.240 to $-327.249\,\text{Ha}$, overlapping with the UDMRG broken-symmetry FCI range -327.244 to $-327.250\,\text{Ha}$ (Zhai et al., arXiv:2601.04621). This shows that TrimCI's robustness here goes beyond what CAS-style initialization provides.

For these reasons we believe "without relying on prior knowledge about good initialization" remains an appropriate description of what TrimCI does.

Different methods have different strengths. Selected CI and coupled cluster are excellent for systems with a large HF weight, where the important determinants are concentrated near a known reference; there is no need to search for it and TrimCI is not the target method. What TrimCI adds to the toolbox is a method for the converse regime: where no such good reference is known a priori, and where conventional initialization routes (CAS in the relevant active space, broken-symmetry single determinants on localized orbitals) are themselves unavailable or inefficient to obtain. We have added the following caveat in the Discussion to de-emphasize the lack-of-prior-knowledge framing: "*We note that when good prior knowledge about the system is available (for example, a large weight on the HF determinant), other methods such as coupled cluster or selected CI perform well and there is no need to use TrimCI; the trim mechanism would still find the correct reference, but with some extra cost relative to methods initialized from the correct reference. The setting in which TrimCI is most valuable is the converse: when no such good reference is known a priori, or*

when obtaining one is inefficient."

Closing Remarks

In response to the Reviewers' detailed feedback in this round, we have conducted several new experiments, sharpened the framing of the manuscript, and improved the writing throughout. The revised version includes: (1) a new orbital-recovery experiment showing that, even from a fully scrambled basis on the $[Fe_4S_4]$ CAS(36o, 54e) Hamiltonian, TrimCI rotates the orbitals back to a basis that allows fast convergence to the FCI limit, while the standard SHCI and DMRG-CASSCF orbital-optimization workflows on the same starting point do not (SI S7); (2) a detailed characterization of TrimCI's practical scale and GPU scaling, with a single-node 2.56×10^9-determinant calculation matching the largest published UDMRG calculation for $[Fe_4S_4]$ at $D = 12{,}000$, and an empirical scaling law $t \propto N_{\text{det}}^{1.2}$ measured across six GPU models that projects to 10^{10}–10^{12} determinants on multi-node setups (SI S8); (3) a sharpened framing in the Discussion that distinguishes the regime in which TrimCI is most valuable (when no good reference is known a priori, or when obtaining one is inefficient) from regimes where conventional methods already work well; and (4) explicit cross-references between the main text and SI sections, and language polish throughout to improve clarity and precision.

Through these new experiments and the responses to each Reviewer's concerns, we have demonstrated that TrimCI offers a qualitatively new way to identify a compact ground-state-relevant subspace in the strongly correlated regions where no good initial reference is known or obtaining it is inefficient, or even no good orbitals are provided. We believe the method and the conceptual insights it provides will shed light on how we understand strongly correlated problems and be of broad interest across quantum chemistry, condensed matter, materials science, and quantum information, and we hope that TrimCI will prove to be a useful tool for researchers across these communities. We are confident that Nature Communications, given its broad interdisciplinary scope, is the right venue for this work.

Sincerely,

Hao Zhang
Matthew Otten

Department of Physics
University of Wisconsin – Madison

Point-by-Point Response to Reviewers (Round 3)

"From Random Determinants to the Ground State"

Manuscript ID: NCOMMS-25-95448B

We thank the Reviewers again for their time and for their thoughtful comments across all three rounds. Reviewer 4's comments are addressed point by point below.

Response to Reviewer 4

Main point: concern about "no prior knowledge"

One of my main concerns about the manuscript was related to the emphasis on "no prior knowledge in the initialization". I had asked the authors to remove this type of wording, a concern shared by the other reviewers.
The authors have essentially rejected this recommendation, and have included some additional numerics which, from my perspective, don't support their argument at all (which I discuss below). I am not sure why the authors are sticking to their guns about this. What puzzles me is:

- *Do the authors* truly *believe their algorithm is* theoretically *efficient without prior knowledge? This must of course be simply false, because even for a purely classical problem of ground-state optimization, the complexity is in NP. Any algorithm that is known to be efficient* must *then rely on prior knowledge. The fact that multiple reviewers have asked the authors to soften their language surely shows that this is not a trivial concern, and insisting on this type of careless language, in my view, simply makes the paper less credible.*
- *The authors may argue that they aren't using any* obvious *problem specific information in their algorithm. I agree with this, but one can't have it both ways - the algorithm can't be highly efficient in general (as discussed above). Therefore, for the subset of problems where it is efficient, it must be using structure - in fact the structure, is (by definition) the one compatible with the initialization algorithm they have chosen! So, if one claims efficiency, it is because one knows that the structure is compatible (even if it's not particularly chemically obvious) - this is the prior knowledge.*
- *From a practical perspective, I pointed out last time that one can initialize selected configuration interaction methods in many different ways, for example using active space wavefunctions or other techniques. The authors then counter that some of these techniques will not be successful in all problems (for practical reasons). But, the same is true of the authors' method. Any initialization procedure can be defeated, for example, one may consider the work in https://arxiv.org/pdf/2603.03496 which is designed to defeat a certain type of selected CI initialization.*

The authors must believe that removing this type of language weakens their paper. My opinion remains that being careful and rigorous in the writing simply prevents them from being attacked and will make for a better paper. For example, "simulated annealing" is a strategy that can be used in NP hard problems - but one wouldn't write "simulated annealing is an efficient algorithm for NP hard problems with no prior knowledge"- something that would lead to consternation – one either writes "simulated annealing is an algorithm which does not use prior knowledge in NP hard problems" (i.e. no claim of efficiency) or "simulated annealing is an efficient algorithm for NP hard problems when it is known that competing minima are separated by barriers that are not too wide".
What I think is defensible to state that the authors have an automated *initialization procedure, which in a variety of problems, they have shown to work quite well. And this is a positive and useful result.*

Response: We sincerely thank the Reviewer for these considerate, insightful comments. We have benefited greatly from this discussion and have learned a lot from it: the Reviewer's argument

sharpened our own understanding of what our method does and does not claim. We now fully accept the recommendation: "automated initialization" is indeed the better description of TrimCI. We have replaced "prior-knowledge-free" with "auto-initializing" throughout the revised manuscript (see the table below), and we have removed the phrase "prior knowledge" in favor of "explicit knowledge" wherever the text describes the problem-specific input that established methods rely on. After this revision, the phrase "prior knowledge" no longer appears in the manuscript or the SI, and the emphasis on "no prior knowledge in the initialization" is gone.

Our original intention with "prior-knowledge-free" was simply to distinguish TrimCI from methods that require an explicitly supplied, problem-specific reference, such as a good reference state, a correct broken-symmetry determinant, or a carefully constructed trial wavefunction. We agree that "prior-knowledge-free" is not rigorous and leads to confusion, and that "auto-initializing" is the better expression. We fully agree with the Reviewer on the tension between "no prior knowledge" and efficiency, and we thank the Reviewer for the excellent simulated-annealing example. Indeed, we cannot claim that the algorithm is efficient for all problems: ground-state optimization is computationally hard in the worst case. What we do observe is that, for the many problems we have studied, the algorithm is empirically very effective: across weakly correlated to strongly correlated systems, starting from random determinants, it efficiently finds a good core wavefunction, from which the expansion converges rapidly to the exact ground state. Examples include weakly correlated molecules, the chromium dimer, iron-sulfur clusters, and the Hubbard model. Regarding the construction the Reviewer pointed us to (Kirby *et al.*, arXiv:2603.03496), we agree that in principle any initialization procedure can be defeated; for this specific construction, however, we have found a way to solve the problem efficiently starting from random determinants, and we will report the results in follow-up work. We also have unpublished results on very strongly correlated systems, such as fractional Chern insulators and the SYK model, where, to our surprise, the method also works well. Yet we agree, as the Reviewer says, that these empirical successes reflect underlying structure of the problems, shared by the typical problems we encounter.

Specifically, we have made the following changes throughout the manuscript and the SI:

**Location**	**Original**	**Revised**
Abstract	"a prior-knowledge-free algorithm"	"an auto-initializing algorithm"
Abstract	"a prior-knowledge-free, accurate and highly efficient framework"	"an auto-initializing, accurate and highly efficient framework"
Introduction	"we explore an prior-knowledge-free approach"	"we explore an auto-initializing approach"
Introduction	"It requires no human-provided knowledge about the system, no pre-designed ..."	"It requires no explicitly provided problem-specific knowledge, no pre-designed ..."

Location	Original	Revised
Introduction	"prior knowledge" (three instances in the survey of established methods)	"explicit knowledge" (all three instances)
Main idea (Motivation)	"identifying a compact core set without prior knowledge of the ground state (e.g., Hartree-Fock)"	"identifying a compact core set without a reference state (e.g., Hartree-Fock)"
Main Results ([4Fe-4S])	"This prior-knowledge-free, trimming-driven process ... with unprecedented efficiency"	"This automated, trimming-driven process ... with such efficiency"
Discussion	"without relying on prior knowledge about good initialization, human-designed ansätze, or guiding reference states"	"without requiring user-supplied initializations, human-designed ansätze, or guiding reference states"
Discussion	"when good prior knowledge about the system is available"	"when good explicit knowledge about the system is available"
SI (benchmark)	"prior knowledge" (all five instances)	"explicit knowledge"

Minor point 1: the [4Fe-4S] energy comparison

For Fe4S4, the authors are still comparing not to the lowest variational energy, but just one of the reported numbers in the cited paper. The reader will presumably assume that the comparison is to the lowest variational energy, so I think the authors have to say that this is not the lowest variational result. At the same time, when reading the main text, I see no indication of this additional result (it still states "Furthermore, extrapolation of variational and perturbatively corrected TrimCI energies yields an ultimate estimate of −327.263 Ha, consistent with the DMRG benchmark result of −327.240 Ha at bond dimension $d = 4000$"). I don't know why the SI result is hidden, but since "benchmark" often means high accuracy, I would at the very least remove benchmark from the main text (and presumably cite the SI here).

Response: We thank the Reviewer for pointing out this outdated statement. We have removed this comparison from the main text, together with the corresponding extrapolation estimate in the caption of Fig. 3. The main text now reports our latest result, obtained with the full workflow including orbital optimization, refers the reader to the SI (S7 and S8) for details, and cites the more recent and more accurate literature results on this system. The revised passage reads:

> "As shown in Fig. 3, TrimCI surpasses this result with dramatically fewer determinants. It reaches −326.810 Ha using only 10^3 determinants and matches the SQD energy of −326.9127 Ha with merely 1.3×10^4 determinants. This is six orders of magnitude fewer

than SQD. Furthermore, with TrimCI's orbital optimization enabled, the full workflow reaches $N_{\text{det}} = 5.12 \times 10^9$ determinants and an extrapolated energy of -327.2441 Ha, within 0.2 mHa of the large-scale UDMRG extrapolation on the same broken-symmetry state (see SI S7 and S8). We also refer the reader to Refs. [77, 78] for more accurate and detailed results on this system."

Minor point 2: the DMRG-CASSCF comparison section

> *The new section on comparing CASSCF optimization with DMRG and selected CI is flawed. The CI wavefunction with 100 determinants can* in the worst case *be represented by a DMRG wavefunction with bond dimension 100, so the fact that the CI wavefunction optimizes better than DMRG with bond dimension 4000 is to do with other things – most likely, the fact that the default initialization procedure in the block2 implementation of DMRG simply assumes that one has a dominant determinant, and the authors did not explore other options. This is just one choice in DMRG that is used in quantum chemistry, but not in other applications of DMRG. It highlights once again that all these methods (DMRG, selected CI) are deployed with a variety of heuristics to initialize, and* require *some heuristic information about the type of problem (in other words, prior knowledge) to be successful.*

Response: We thank the Reviewer for this analysis and have added a caveat at the end of the new section:

"We note a caveat on the interpretation of this comparison. A compact CI wavefunction can in principle be represented by an MPS of modest bond dimension, so the difficulty observed here is not one of expressive power. Moreover, we did not explore alternative MPS initializations for the inner DMRG solver. This comparison should therefore be read as characterizing this particular workflow within the current implementation of Block2, rather than the intrinsic capability of DMRG."

In summary, we have removed the "prior knowledge" language throughout the manuscript and the SI, adopting the "auto-initializing" formulation, and revised the [4Fe-4S] comparison and the DMRG-CASSCF section as described above. We would like to thank Reviewer 4 once more for the insightful and constructive comments across all three rounds, which we believe have indeed helped a lot in making this a better paper.

In the manuscript, the authors introduce a selected-CI algorithm, TrimCI, which aims to efficiently identify an important fraction of the Hilbert space through an iterative search and refinement procedure. The method combines heat-bath screening (Eq. 5) with a trimming step applied during the variational stage. The algorithm is clearly described, and its scope is well stated.

According to the authors, the major contributions of the proposed method are that it (1) is prior-knowledge-free, (2) can identify a better variational space than the semistochastic heat-bath CI (SHCI) method, and (3) provides highly accurate results for strongly correlated systems. Numerical results are presented in support of these claims. However, there are several major concerns that seriously undermine the claimed contributions of the proposed method. These concern are detailed below, and must be clarified before the manuscript can be considered for publication.

Major revision:

1. **Physical significance of the results is not sufficiently demonstrated.** For all chemical systems presented, Cr2, Fe4S4 and P-cluster, the authors report that the selected space by TrimCI is much different from that of SHCI, and concentrated near "the physically relevant region" of the Hilbert space. What is the physical relevant region? The authors should discuss more about the one-electron basis used. Did they use HF split-localized (or other forms of localization) or HF canonical orbitals (delocalized)?
If they used HF canonical orbitals, clearly the closed-shell HF determinant, |2222222000000000...>, will certainly be one of the leading determinants of the ground-state wave function (even though its weight is small). In this scenario, I would actually expect that the SHCI, while having higher energy, is capturing the correct physics (given that the star is part of the selected space), while TrimCI is missing it (given that all dets are far from the star in their figures).
If they used localized orbitals, then any closed shell determinant, including HF determinant, would have NO contribution to the ground-state wave function. Instead, the |aaaaaaa,bbbbbbbb, 00000...> might have larger contribution (a and b = alpha and beta electrons) albeit still generally small. The prior knowledge in this aspect is necessary! Initializing SHCI from |aaaaaaa,bbbbbbbb, 00000...> would be a good choice.
I suspect that the authors either have initialized SHCI in a physically poor way, and derailed the SHCI search, or they put little emphasis on the physics/chemistry of the problem, while focusing too much on the numerical aspects.
If they want to show the strength of TrimCI, they should not rely exclusively on the poor initialization of SHCI. Instead they should guide the SHCI search in a physically meaningful way, and then compare the results against their TrimCI.
Energy in this context is really not a good measure of quality. It is not to be excluded the following scenario: The ground state wave function is highly multi-determinantal, while the excited state is more compact. A truncated CI would converge faster for the excited state than for the ground state. Both TrimCI and SHCI are far from converged, as demonstrated by the steep lines in Fig.2 (a, d, g) as a function of the selected space. However, TrimCI might have got stuck to the hypothetical excited state, and the more compact representation is a reflection of the compactness of the excited state. In this scenario the excited state would be artificially stabilized as compared to the ground state, as TrimCI is capturing more correlation for the excited state (whose wave function is more compact) than the SHCI does for the ground state.
To avoid any misinterpretation of the data, I advise the authors to analyze the leading determinants in both SHCI and TrimCI, and discuss their physical significance. Even better, if authors have access to observables (expectation values, easily accessible via RDMs), such as spin-spin correlation functions, they should compare those values from both methods. Only by analyzing the physical content of the wave function (leading determinants, observables) one can rule out such a possibility. This becomes more important for the larger clusters (say the Fe4S4 and P-cluster), where the low energy spectrum is dense and as demonstrated by Ref. 62, it is easy to get trapped in an excited state.
And if the SHCI is poorly initialized, the authors should redo the SHCI calculations with a better initial space, and compare again with TrimCI. Only then it would be clear the superiority of TrimCI over SHCI, not just in the identification of the trial space. The authors could still highlight the advantage of TrimCI in being prior-knowledge-free, but at least the energy and convergence between the two methods would be compared on a fair ground.

2. **Only the variational stage of SHCI and TrimCI is compared.** The comparison presented in the manuscript is restricted to the variational stage of the two methods. However, a key strength of SHCI lies in its semistochastic perturbative stage, which largely alleviates the memory bottleneck and is essential to its overall performance. While I understand that the distinguishing feature of TrimCI is the trimming step applied during the variational stage—and that, in principle, this strategy could be combined with the perturbative stage of SHCI—the way the comparison is currently presented gives the impression that

TrimCI outperforms SHCI as a complete method.
Such a conclusion cannot be drawn without explicitly comparing the fully extrapolated PT2 energies obtained from the original SHCI protocol and the TrimCI with PT2 extrapolation. Without this comparison, it is difficult to assess whether TrimCI provides a genuine advantage over existing methods.
In addition, it would be valuable to compare TrimCI with other selected CI methods specifically designed for highly multireference systems, such as TPSCI (https://dx.doi.org/10.1021/acs.jctc.0c00141). In the referenced work, the authors compare TPSCI and HCI across all stages of the calculation and demonstrate that TPSCI achieves lower variational energies with smaller variational spaces, particularly for highly multireference systems (e.g., stretched diatomic molecules in Table 1). Notably, the PT2 energy differences between HCI and TPSCI are much smaller than the corresponding differences at the variational level, highlighting the importance of comparing complete workflows rather than only the variational stage.

3. **The computational parameters used in the calculations are not sufficiently specified or tested.**
In Fig. 2, the authors compare the variational energies of TrimCI and HCI as a function of variational space size, showing that TrimCI can reach similar variational energies with substantially smaller variational spaces. However, this apparent advantage is meaningful only if the computational cost and memory requirements required to reach the final variational space are not substantially higher than those of HCI. For example, in an extreme scenario where the parameter "pool_core_ratio" is chosen so large that the TrimCI calculation already requires memory comparable to a full CI calculation, the reduction in variational space size would not constitute a practical advantage. Moreover, since TrimCI introduces overhead due to multiple subspace diagonalizations, claims of computational efficiency based solely on Fig. 2 are not convincing without providing the relevant trimming and expansion parameters.
I also assume that the HCI calculations are initiated from the Hartree–Fock (HF) determinant, while the TrimCI calculations employ the ensemble strategy. This information should be explicitly stated in the manuscript. If this assumption is correct, the ensemble strategy should also be applied to the HCI calculations to ensure a fair comparison. In my view, the choice of the initial determinant set is a user-defined input rather than a method-specific feature. For strongly correlated systems in which the HF determinant lacks clear physical meaning, initializing the calculation from a single HF determinant is not a reasonable option.

4. **The prior-knowledge-free feature is not sufficiently demonstrated.**
A central selling point of TrimCI emphasized throughout the manuscript is its prior-knowledge-free property. Two aspects contribute to this feature: (i) the ensemble strategy, in which several simulations are initiated from different random variational spaces and the best-performing one is selected after a few iterations, and (ii) the local trimming step, which removes determinants based on local information within each variational subspace. However, this claim is not sufficiently supported by the presented results.
Regarding the first aspect, the sensitivity of the method to the initial composition of the variational space needs to be systematically investigated. If the performance is strongly dependent on the starting space, this could limit the applicability of the method to large systems, where identifying a favorable random initial space becomes increasingly unlikely.
As the ensemble strategy appears more like a user choice than an intrinsic feature of the method, additional evidence is required to demonstrate that the prior-knowledge-free behavior is primarily attributable to the trimming step itself. Although Fig. 5 partially illustrates this point, using 10,000 random determinants for the 4×4 real-space Hubbard model—where Néel states are clearly defined and easily reachable—does not seem to constitute a rigorous test. While Figs. 5(a) and 5(b) are interesting in that the correct time-reversal symmetry is eventually recovered once the variational space reaches approximately 1.7e5 determinants, the number of iterations and the expansion ratios required to achieve this convergence should be explicitly reported.

5. **PT2 extrapolation results are not clearly presented.**
The PT2-extrapolated energies are benchmarked against FCI and AFQMC results, primarily in Table 2. However, the table does not clearly indicate the range of variational space sizes used for the extrapolation. In Fig. 4(b), the energies of the data points used for extrapolation are all lower than -18.014, whereas the corresponding energies reported in Table 2 are -18 or higher. This presentation is potentially misleading, as it may give readers the impression that highly accurate extrapolated energies can be obtained from very low-cost calculations.
In addition, some variational space sizes reported in Table 2 are estimated rather than explicitly given. Since the determinants in the variational space are presumably stored in memory, it is unclear why the exact number of determinants cannot be reported. Clarification is needed regarding how these estimates are obtained and how accurate they are.

6. In the intro the authors cite ref 62 as a method that requires prior-knowledge. In what aspect that work used prior-knowledge? I might understand that some selected-CI rely on the initial trial wave function, a reference determinant. However, in DMRG work, I would exclude such situations. The authors might want to clarify that point.

7. Very recently the following work has come out: https://doi.org/10.1021/acs.jctc.5c01264. The authors might want to follow that line of research and relate their findings to that. Clearly, a compressed wave function would also favor the TrimCI algorithm (once implemented into a spin-adapted basis).

Minor comments:

1. Typo: Right above Eq.5 the authors write "pool set P. by including". We suppose the period after P is a typo.
2. Authors use the short notation dets for determinants in several places. I would avoid such abbreviations in a formal article and use the extended form or the well accepted SD acronym.
3. The authors state that the initial space "satisfy the required particle number and spin constraints." They cannot enforce spin symmetry as they are using SDs. They can only enforce the correct S_z value (spin projection). Please correct this statement.
4. Symbols appearing in Eq. (4) are not defined. For completeness, these should be explicitly introduced.
5. The selection criterion ($|H_{ij}c_j| > \theta$) does not include an explicit sum over j and allows inexpensive expansion, as noted by the authors. Since this criterion is a key ingredient of the heat-bath CI algorithm (https://doi.org/10.1021/acs.jctc.6b00407), I recommend explicitly referencing this work when describing the expansion step.
6. The bond length of the chromium dimer is missing from the caption of Fig. 2, although it is mentioned in the main text.
7. It is unclear which variational spaces are used for the MDS plots in Fig. 2. Are these spaces chosen to have the same size or the same variational energy?
8. Is the term "Hamming distance" equivalent to the excitation level relative to a reference determinant? If so, using the latter term would provide a more direct physical interpretation.
9. Several figures would benefit from improved quality. In Fig. 1(c), N is not rendered as a superscript and some numbers are obscured by the black circle. In addition, the small markers in the MDS plots appear as white dots in my PDF viewer unless zoomed in, likely due to marker boundary rendering.

At first glance the method appears capable of generating improved variational spaces for subsequent perturbative corrections. However, a more fair and comprehensive comparison with existing methods is necessary to properly assess the performance of the proposed approach. Details regarding computational parameters and the data used for PT2 extrapolation are also required, along with a more systematic investigation of their impact on convergence.

Even aside from the concerns discussed above, the scope and significance of the results currently presented do not appear sufficient for publication in Nature Communications. I therefore recommend that the manuscript would be more appropriate for more technically oriented journals, such as JCP or JCTC, after the above concerns have been adequately addressed.
